# Phylogenomic analysis of Apoidea sheds new light on the sister group of bees

**DOI:** 10.1186/s12862-018-1155-8

**Published:** 2018-05-18

**Authors:** Manuela Sann, Oliver Niehuis, Ralph S. Peters, Christoph Mayer, Alexey Kozlov, Lars Podsiadlowski, Sarah Bank, Karen Meusemann, Bernhard Misof, Christoph Bleidorn, Michael Ohl

**Affiliations:** 1 Museum für Naturkunde, Leibniz Institute for Evolution and Biodiversity Science, Invalidenstraße 43, 10115 Berlin, Germany; 2 Center for Molecular Biodiversity Research, Zoological Research Museum Alexander Koenig, Adenauerallee 160, 53113 Bonn, Germany; 3 University of Freiburg, Institute of Biology I (Zoology), Evolutionary Biology and Animal Ecology, Hauptstr. 1, 79104 Freiburg, Germany; 4 Center of Taxonomy and Evolutionary Research, Arthropoda Department, Zoological Research Museum Alexander Koenig, Adenauerallee 160, 53113 Bonn, Germany; 5 HITS gGmbH, Heidelberg Institute for Theoretical Studies, Schloss-Wolfsbrunnenweg 35, 69118 Heidelberg, Germany; 6 Georg-August-Universität Göttingen, Animal Evolution and Biodiversity, Untere Karspüle 2, 37073 Göttingen, Germany; 7 German Centre for Integrative Biodiversity Research (iDiv) Halle-Jena-Leipzig, Deutscher Platz 5e, 04103 Leipzig, Germany

**Keywords:** Hymenoptera, Aculeata, Apoidea, Apoid wasps, Bees, Anthophila, Target enrichment, Phylogeny, Evolution

## Abstract

**Background:**

Apoid wasps and bees (Apoidea) are an ecologically and morphologically diverse group of Hymenoptera, with some species of bees having evolved eusocial societies. Major problems for our understanding of the evolutionary history of Apoidea have been the difficulty to trace the phylogenetic origin and to reliably estimate the geological age of bees. To address these issues, we compiled a comprehensive phylogenomic dataset by simultaneously analyzing target DNA enrichment and transcriptomic sequence data, comprising 195 single-copy protein-coding genes and covering all major lineages of apoid wasps and bee families.

**Results:**

Our compiled data matrix comprised 284,607 nucleotide sites that we phylogenetically analyzed by applying a combination of domain- and codon-based partitioning schemes. The inferred results confirm the polyphyletic status of the former family “Crabronidae”, which comprises nine major monophyletic lineages. We found the former subfamily Pemphredoninae to be polyphyletic, comprising three distantly related clades. One of them, Ammoplanina, constituted the sister group of bees in all our analyses. We estimate the origin of bees to be in the Early Cretaceous (ca. 128 million years ago), a time period during which angiosperms rapidly radiated. Finally, our phylogenetic analyses revealed that within the Apoidea, (eu)social societies evolved exclusively in a single clade that comprises pemphredonine and philanthine wasps as well as bees.

**Conclusion:**

By combining transcriptomic sequences with those obtained via target DNA enrichment, we were able to include an unprecedented large number of apoid wasps in a phylogenetic study for tracing the phylogenetic origin of bees. Our results confirm the polyphyletic nature of the former wasp family Crabonidae, which we here suggest splitting into eight families. Of these, the family Ammoplanidae possibly represents the extant sister lineage of bees. Species of Ammoplanidae are known to hunt thrips, of which some aggregate on flowers and feed on pollen. The specific biology of Ammoplanidae as predators indicates how the transition from a predatory to pollen-collecting life style could have taken place in the evolution of bees. This insight plus the finding that (eu)social societies evolved exclusively in a single subordinated lineage of apoid wasps provides new perspectives for future comparative studies.

**Electronic supplementary material:**

The online version of this article (10.1186/s12862-018-1155-8) contains supplementary material, which is available to authorized users.

## Background

The Apoidea (apoid wasps and bees) comprise approximately 30,000 known species [1, 2] and represent the most species-rich superfamily within Aculeata (stinging wasps, ants, and bees). Apoidea are remarkable for their evolution of many different life styles (e.g.*,* cleptoparasitism, solitary predatory life, eusociality) and corresponding morphological adaptations [3–5]. Within Apoidea, the morphologically and behaviorally heterogeneous apoid wasps comprise approximately 10,000 described extant species, which are currently assigned to one of four families each: Ampulicidae, Crabronidae, Heterogynaidae, Sphecidae sensu stricto (*s. str.*) [2]. Most apoid wasp species have a predatory life style: they typically provide paralyzed prey from a wide range of terrestrial arthropods (e.g.*,* Anthophila, Diptera, Lepidoptera, Orthoptera) as food source for the developing larvae [6, 7]. The vast majority of apoid wasps are solitary, but social life histories ranging from communal nesting to eusociality are known from species of the genera *Microstigmus*, *Cerceris,* and *Spilomena* [8–10].

With approximately 20,000 described species, the monophyletic bees (Anthophila) harbor about two-thirds of all apoid species [1]. Bees primarily feed on pollen and nectar, both as larvae and adults, and serve as important pollinators of crop and wild plants [11]. The comparatively well-studied bees have been used as models for studying the evolution of sociality, intra- and interspecific communication, and physiological adaptations [4, 12–16]. While most bee species are solitary, bees are well-known for having evolved various levels of social life styles, ranging from communal nesting to life in highly sophisticated eusocial societies [17, 18]. Our understanding of both, the evolution of social life-histories within Apoidea and the transition from entomophagous hunting (apoid wasps) to pollen-collecting (bees), relies on a solid knowledge of the phylogenetic tree of Apoidea with adequate taxon sampling. Previous phylogenetic analyses suggested that Apoidea as well as Anthophila are well-supported clades, whereas apoid wasps represent a paraphyletic group with respect to Anthophila [12, 19–23]. The closest extant relatives of bees among apoid wasps, however, have remained unclear [12, 23–26]. Knowledge of the group of apoid wasps that bees are particularly closely related to has the potential to shed light on morphological and behavioral traits in the last common ancestor of bees that might enable better understanding of the tremendous diversification of Anthophila. During the last decade, it has been proposed that bees are either the closest relatives or a subordinated lineage of the family “Crabronidae” [12]. In a recent study on the phylogeny of Hymenoptera, Peters and colleagues (2017) found the genus *Psenulus* (“Crabronidae”: Pemphredoninae) as closest relative of bees. While the taxonomic sampling of apoid wasps analyzed by Peters and colleagues (2017) was already comprehensive, it did not include possible alternative sister group candidates, like the tribes/subtribes Ammoplanina, Stigmina, Odontosphecini of the subfamily Pemphredoninae or the apoid wasp family Heterogynaidae, which is monotypic and whose phylogenetic position within the apoid wasps has been controversially discussed [24, 27]. A study on the phylogeny of the stinging wasps (Aculeata) [28], simultaneously published to that by Peters et al. (2017), included a species of the enigmatic Heterogynaidae (i.e.*, Heterogyna nocticola*) in their taxonomic sampling and inferred a sister group relationship of Heterogynaidae to “Crabronidae” (*partim*) + Sphecidae. Branstetter et al. (2017) also considered one representative of Ammoplanina, *Pulverro boharti*, which together with *Pluto argentifrons* clustered as sister group of Philanthinae. However, the taxon sampling of “Crabronidae”, and especially of Pemphredoninae and Philanthinae, in the latter study was scarce and prevented drawing further conclusions on specific phylogenetic origin of bees.

Here we present results from inferring phylogenetic relationships of Apoidea by studying a dataset including 195 molecular (sequence) markers with the most comprehensive taxonomic sampling of Apoidea so far. Our taxonomic sampling comprises a total of 174 species, representing all described extant apoid wasp subfamilies (except for Eremiaspheciinae), almost all major lineages of Pemphredoninae and Philanthinae, and all currently recognized bee families (i.e., Andrenidae, Apidae, Colletidae, Halictidae, Megachilidae, Melittidae, Stenotritidae). We made use of a custom set of RNA baits to enrich 195 single-copy protein-coding genes of apoid wasps and bees [29]. The inferred phylogenetic relationships and divergence time estimates (**1**) present a novel hypothesis on the evolutionary history of apoid wasps and bees, (**2**) are used to study the phylogenetic origin and to estimate the geological age of bees, and (**3**) shed light on the switch from a predatory to herbivorous life style. Beyond, we propose a new classification of major lineages of apoid wasps, splitting polyphyletic groups into monophyletic taxonomic units.

## Results and discussion

### Sequencing, orthology assessment, and data processing

We collected between 0.06 and 3.4 M paired-end quality-trimmed raw reads per species. These reads assembled on average into 17,551 contigs. After having compared all contigs sequenced on the same lane against each other, we removed on average ~ 7.1% potential cross-contamined contigs per species (Additional file 1: Table S1). We successfully enriched on average 71% of the target DNA across all species. The base coverage depth of the on-target contigs (*C*_*t*_) was on average 967× (Additional file 1: Table S1). When searching for the 195 target genes in the sequenced and assembled enriched DNA libraries, we found on average 139 target genes per species. In comparison, when searching the available transcript libraries [27|, we found on average 187 target genes per species (Additional file 1: Table S1 and S2). Additional sequencing information on, for example, length of contigs referring to target genes and the number of identified orthologs per species is given in Additional file 1: Table S1 (enrichment data set) and in Additional file 1: Table S2 (transcriptomic data set). We furthermore provide additional supplementary results of all conducted processing steps of the aligned multiple sequence alignments (MSAs) on nucleotide and amino acid level on, for example, alignment reliability and masking and protein domain identification is given in the Supplementary information.

### Phylogenetic analyses

Our phylogenetic inferences are based on enriched nucleotide sequence data of 95 and on transcriptomic sequence data of 79 apoid wasp and bee species. We added corresponding sequence data of nine outgroup species for rooting the inferred tree topology. The analyzed dataset comprises 94,869 amino acid and 284,607 corresponding nucleotide sites (representing all codon positions), encoding a total of 195 single-copy protein-coding genes. For estimating divergence times, we applied an independent-rate molecular clock approach considering ten validated fossil calibration points [30, 31] (Additional file 2: Figure S1 and Additional file 1: Table S9). Information on taxa with unstable phylogenetic position (rogue taxa) can be found in the Supplementary information and in Additional file 1: Table S3.

**Fig. 1:**
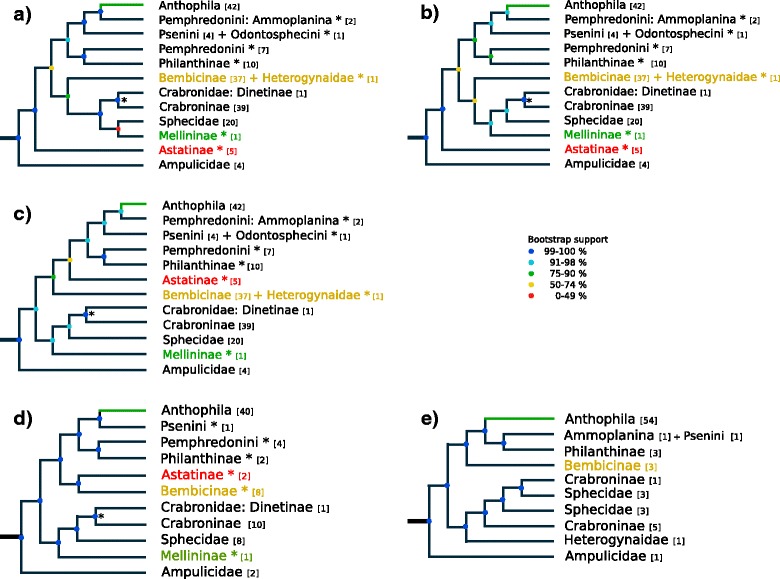
Possible phylogenetic relationships of the major apoid wasp lineages and of bees (Anthophila) as inferred in the present investigation and by Peters et al. (2017). Members of the apoid wasp family “Crabronidae” are scattered across eight major clades, whereby we combine Crabronidae: Dinetinae and Crabroninae to one clade: Crabronidae (marked by an asterisk). Numbers in brackets represent the number of taxa inluded in the analyses. Highlighted group names of Astatinae (red), Bembicinae (yellow) and Mellininae (green) show unambiguous sister group relationships, resulting in a total of three alternative tree topologies: (**a**) inferred from analyzing 284,607 nucleotide sites and applying a combination of protein domain – and codonbased partitioning scheme by modeling 1st, 2nd and 3rd codon positions separately, (**b**) inferred from analyzing 284,607 nucleotide sites and applying a combination of protein domain – and codon-based partitioning scheme by modeling 1st and 2nd codon position separately - 3rd codon position excluded, (**c**) inferred from analyzing 94,869 amino acid sites and applying a protein domain-based partitioning scheme, (**d**) inferred by Peters et al. (2017) and (**e**) inferred, including bootstrap support values by Branstetter et al. (2017)

**Fig. 2:**
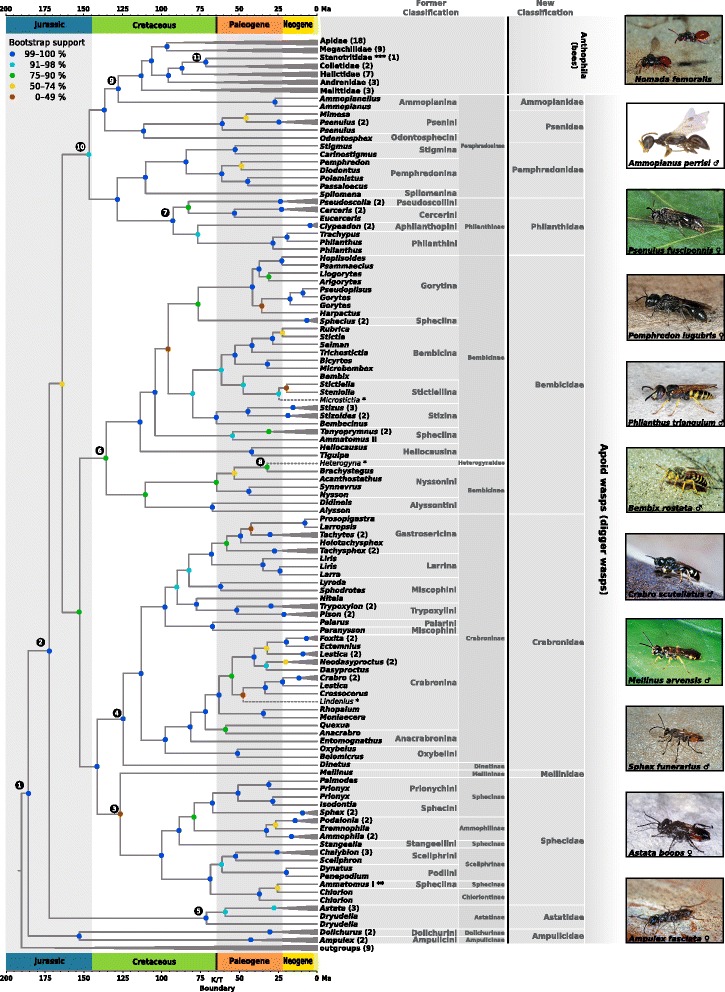
Maximum likelihood phylogenetic tree inferred from analyzing 284,607 nucleotide sites and applying a combination of protein domain – and codon-based partitioning scheme by modeling 1st, 2nd and 3rd codon positions separately. Support values are obtained from 100 bootstrap replicates. Species marked by an asterisk (*) indicate rogue taxa. Two asterisks (**) point to the misplaced species Ammatomus sp. I and (***) to the position of the Stenotritidae. Circled numbers (nodes) indicate taxonomic groups of special interest described in the main text. Former classification according to W. J. Pulawski’s Catalog of Sphecidae “sensu lato”

We inferred largely congruent topologies, irrespective of whether we analyzed the amino acid or nucleotide sequence data (1st and 2nd codon positions only as well as all three codon positions) under the maximum likelihood optimality criterion (Additional file 2: Figures S2, S3 and S4), and almost all clades received high bootstrap support (Fig. 1). Our analyses confirm the monophyly of Apoidea, as previously suggested by analyzing morphological characters and molecular sequence data [24, 27, 32, 33] (Fig. 2; node **1**). We estimate the origin of Apoidea to have been in the late Jurassic, ca. 185 million years ago [Mya] (95% confidence interval [CI] 220–165; node **1**), indicating that Apoidea are likely older than previously thought [27, 34–36]. We confirm Ampulicidae as closest extant relatives of all remaining Apoidea. Our results show the family Crabronidae to be polyphyletic, a result consistent with earlier studies [12, 23–25, 27] (Fig. **2**; node **2**). Our study confirms the monophyly of each of the species-rich crabronid wasp subfamilies Astatinae, Bembicinae + Heterogynaidae, Crabroninae + Dinetinae**,** Philanthinae, and of the apoid wasp family Sphecidae (classification according to Pulawski 2016). The phylogenetic placement of the species-poor crabronid wasp subfamily Mellininae, currently included in the Crabronidae, differs between topologies inferred from analyzing different datasets: in the topology inferred from analyzing all codon positions on the nucleotide sequence level (Fig. **2**; node **3**) Mellininae are suggested as the sister group of Sphecidae, although with low bootstrap support. In contrast, in the topology inferred from analyzing the amino acid sequence data (Fig. 1c) and in the topology inferred from analyzing only 1st and 2nd codon positions (Fig. 1b), Mellininae are the sister lineage of (Sphecidae + (Craboninae + Dinetinae). The latter placement of the Mellininae was also inferred by Peters et al. (2017), although with higher bootstrap support than what we found in our study (Fig. 1d). We inferred Craboninae and Dinetinae as sister groups, irrespective of the analyzed datasets (Fig. 1a–1c and Fig. 2; node **4**). In contrast to Branstetter et al. (2017), we find the apoid wasp family Heterogynaidae to be a subordinated lineage of Nyssonini, a tribe of the crabronid wasp subfamily Bembicinae (Fig. 2; node **8**), but with poor bootstrap support. Finally, we confirm the polyphyly of the species-rich crabronid subfamily Pemphredoninae as suggested by Peters et al. (2017), representing an artificial group of three lineages. One of these three lineages, the one comprising Stigmina, Pemphredonina, and Spilomenina, is inferred as the sister lineage of the crabronid wasp subfamily Philanthinae (Fig. 2; node **7**). The remaining two lineages (i.e.*,* Psenini + Odontosphecini and Ammoplanina) constitute a paraphyletic grade leading to Anthophila. Since the taxon sampling in the study by Peters et al. (2017) neither included Ammoplanina nor Odontosphecini, the authors inferred Psenini as the closest relatives of bees. With a more comprehensive taxon sampling, our study is thus the first to suggest that the closest relatives of Psenini are Odontosphecini and that Ammoplanina possibly represents the extant sister lineage of bees. Note, however, that our taxon sampling does not include representatives of the apoid wasp tribe Entomosericini, a lineage that could thus be even more closely related to bees than the Ammoplanina. In any case, the insight of a close phylogenetic relationship between Ammoplanina and bees allows us to further specify the age of the last common ancestor of bees. Specifically, we estimate that the lineage leading to extant bees began to diverge from the lineage leading to the Ammoplanina in the Early Cretaceous, ca. 128 Mya (CI: 148–108 Mya), thus at a time period during which angiosperms rapidly radiated [11, 14, 36, 37] (Fig. 2; node **9**).

The results of the Bowker’s matched-pairs test of symmetry indicate that the nucleotide dataset with all codon positions included (PF-NT-1,2,3) strongly violates the assumptions of global stationarity, reversibility, and homogeneity. In contrast, the amino acid dataset suffers significantly less from these model violations (Additional file 2: Figure S6). Since both inferred trees, from amino acid and nucleotide level (using ML and Bayesian approach) resulted virtually in the same major clades, we conclude that a possible GC bias of single species had most likely no impact on the obtained major results.

We applied Four-cluster Likelihood Mapping (FcLM) to assess whether or not confounding signal due to compositional heterogeneity across taxa and/or confounding signal due to non-random distribution of missing data in the amino acid and in the nucleotide supermatrices could have influence the phylogenetic tree inference. Specifically, we used FcLM to further assess the possible relationships of **a**) Ammoplanina, Psenini + Odontosphecini, Anthophila, and all remaining species in our dataset to each other (Hypothesis 1), and of **b**) Mellininae, Sphecidae, Crabroninae + Dinetinae, and all remaining species in our dataset to each other (Hypothesis 2) (Additional file 3: Table 1). When testing the phylogenetic position of Ammoplanina, we found a strong signal for Ammoplanina being the sister group of the bees when analyzing the nucleotide sequence data. When analyzing the amino acid sequence data, however, the results were inconclusive, as we found support for both a sister group relationship of Ammoplanina and bees and a sister group relationship of Ammoplanina and (Psenini + Odontosphecini). This phylogenetic ambiguity in respect of Ammoplanina is also reflected to some extend by the low bootstrap support for a sister group relationship of Ammoplanina and bees in the phylogenetic tree interred from analyzing the amino acid sequence data. Permutation tests cannot completely exclude confounding signal, more likely because of model violation due to among lineage heterogeneity than non-random distribution of missing data. However, when comparing the proportion of quartets without quartets showing confounding signal, we still have a higher support for Ammoplanina being the sister group of the bees than Ammoplanina being the sister group of Psenini + Odontosphecini (Additional file 1: Table S10, Hypothesis 1). In order to gain further confidence in hypothesis 1 describes the actual evolutionary history of the group, we suggest increasing the taxon sampling within Pemphredoninae (Fig. 2, former classification).

We also assessed the phylogenetic position of Mellininae via FcLM and found strong signal for Mellininae being the sister group of (Sphecidae + (Crabroninae + Dinetinae)) irrespective of whether or not we analyzed amino acid and nucleotide sequence data. This result is congruent with the outcome of the phylogenetic analysis of the amino acid sequence data, but it is incongruent with the phylogenetic tree inference results from analyzing the nucleotide sequence data. While we did not find confounding signal in amino acid sequence dataset when applying permutation tests, we found such signal when applying these tests on the nucleotide sequence data. The confounding signal is likely due to compositional heterogeneity among taxa, and the confounding signal could have caused a model violation in the phylogenetic tree inference, possibly having resulted in a misplacement of Mellininae as sister group to Sphecidae. This possibly erroneous phylogenetic relationship is seen in the ML tree that was inferred from the nucleotide supermatrix that included all three codon positions (Fig. 1a). We here consider the position of Mellininae as sister to (Sphecidae + (Crabroninae + Dinetinae)) to be the more credible hypothesis. For more information on the FcLM results, see Additional file 4: Figures 2, 3, 4 and 5 and Additional file 3: Table 1 in the Supplementary file and Additional file 1: Table S10.

### Sister group of the bees

Our analyses reveal Ammoplanina as possibly representing the extant sister group of bees (Fig. **2**). Ammoplanina comprise ca. 130 species in a total of ten genera [2]. All species of Ammoplanina are remarkably small in size. They have a body length of 2–4 mm and a strongly reduced wing venation. Ammoplanina occur in the Holarctic and in the Ethiopian region [38]. Assuming that Ammoplanina are the closest extant relatives of bees, it might be conceivable that the most recent common ancestor of Ammoplanina and bees was characterized by a small body size, an assumption that fits with the characteristics of a previously described bee fossil, *Melittosphex burmensis* [39]. This supposedly earliest bee fossil, described from 99-Mya-old Myanmar amber, also has a small body size (2.95 mm) and is thus similar in size to species of Ammoplanina. The small body size of *M. burmensis* has been interpreted as an adaptation to the small size of the flower in the Early to Late Cretaceous [39]. In general, it is assumed that the origin of bees and their diversification was strongly linked to the Cretaceous radiation of angiosperms [12, 27, 36, 39]. During the Cretaceous the majority of flowers in Myanmar amber show a size range of 0.5–3.0 mm [40]. It is likely that the small flowers of the Cretaceous were primarily pollinated by small flies, beetles, and thrips, as well as by small Hymenoptera [41], such as the predatory ancestors of bees and early bees.

In this regard, our analyses partly support an almost 50-year-old idea proposed by Sergei Ivanovitch Malyshev (1968) [42]. Malyshev hypothesized that bees are derived from pemphredonine wasps. He deduced his hypothesis from the hunting behavior of some Pemphredoninae on flowers: most pemphredonine and all Ammoplanina wasps hunt for flower-visiting thrips as food for their offspring [43, 58]. Our results allow the reconstruction of an evolutionary scenario for the transition from hunting Ammoplanina wasps to pollen-collecting bees. Species in the Ammoplanina have specialized on thrips, which have been shown to often aggregate on flowers and to feed on pollen [44, 59, 60]. It can be assumed that the visual and potentially olfactorial floral cues, which wasps have used to locate their prey (flower-visiting thrips) could similarly be employed for locating pollen resources by the proto-bee. Pollen-fed and pollen-covered thrips are transported to the nest by the female wasp, and this might have allowed for the switch from thrips consumption to obligatory pollen feeding by the wasp respectively early bee larvae. Malyshev (1968) also stated that the pemphredonines necessarily have to visit flowers and collect thrips repeatedly to provision a nest cell with the sufficient number of prey specimens due to the small prey size. This implies that progressive provisioning was already accomplished by the wasp-like ancestor of the bees. This specific behavior likely further facilitated the transition to pollen collecting, which is also progressive in bees. Thus, in the light of our inferred phylogeny of apoid wasps, an evolutionary shift from predation on flower visiting and pollen feeding thrips in the common ancestor of Ammoplanina and bees to pollen feeding in the stem species of bees appears plausible.

### Evolution of sociality in apoid wasps and bees

While most Apoidea are solitary, a social behavior, such as communal nesting and eusociality, evolved in some apoid wasp lineages. Communal nesting means that a group of females share a nest to lay their eggs and provision them. However, the females neither collaboratively care for the food nor do they form castes. In contrast, eusocial species live in colonies with castes that show a division of labor (“queens” and “workers”) and overlapping generations [18, 45].

The most widespread form of sociality (besides brood care) in Apoidea is communal nesting [17, 18]. In bees, communal nesting is known in Andrenidae, Apidae, Halictidae, Megachilidae, and Melittidae [17, 18], whereas in apoid wasps, it occurs exclusively in some pemphredonine and philanthine wasps. In pemphredonine wasps, communal nesting is known to occur in species of (**1**) Spilomenina (e.g.*, Spilomena socialis* [10], *Arpactophilus mimi* [46], and species of the genus *Xysma* [46]) and (**2**) Stigmina (e.g.*, Carinostigmus* [47]). In philantine wasps, communal nesting is known to occur in, for example, *Cerceris antipodes* [48], *Cerceris rubida* and *Trachypus petiolatus* [49]). In apoid wasps, *Microstigmus comes* (Pemphredoninae) is the only species considered eusocial, based on family groups inhabiting a nest, each with a single mated and size-structured reproductive female [50]. In contrast, ca. 10% of the known bee species are eusocial [17]. Traits, such as intraspecific communication or nesting biology might have triggered the manifold evolution of social behavior in apoid wasps and bees. Solitary species require, for example, intraspecific communication primarily for mate finding and recognition. Species that show communal nesting additionally need to exchange group information, for example, females that compete for reproductive dominance. Here, the relationship between cuticular hydrocarbon profile and ovarian activity informs both competitors and potential helpers about the reproductive potential of each female and can be used to establish dominance and induce helping behavior [45]. Finally, eusocial species need to communicate dominance hierarchies and coordinate division of labor [45]. The results of our phylogenetic study indicate that within Apoidea, social behavior (communal nesting and eusociality) exclusively occurs in representatives of a single clade, which comprises bees, pemphredonine and philanthine wasps (Fig. **2**; node **10**).

The phylogenetically restricted occurrence of species showing some form of social behavior within Apoidea raises the question of whether there might be a common physiological, ecological or morphological trait fostering the establishing of this intriguing trait. In this respect, the role of (chemical) communication in social and non-social species is highly relevant, particularly concerning (**1**) modes of communicatio*n* (e.g.*,* among/within sexes and among/within groups), (**2**) cuticular hydrocarbons (genetically, food-, nest-, and climate-driven [51, 52]), and (**3**) the origin of pheromones from different glands (e.g.*,* postpharyngeal gland and Dufour’s gland [53, 54]). Therefore, further studies on the above listed traits are needed to provide deeper insights in the evolutionary origin of sociality in Apoidea [45].

### Implications for the classification of Apoidea

Our phylogenetic analysis of Apoidea confirmed the polyphyletic status of “Crabronidae”, which comprise about 90% of all known extant apoid wasp species [4]. Two of the currently described four families of apoid wasps (i.e.*,* Heterogynaidae and Sphecidae *s. str.*) are nested deeply within crabronid wasps (Fig. **2****;** nodes **3** and **8**). We identified ten major clades that can be consistently distinguished within crabronid wasps across all inferred topologies. Regarding the still ambiguous sister group relationships of Astatinae, Bembicinae, and Mellininae, we suggest assigning nine of these major clades family rank (i.e.*,* Ammoplanidae, Astatidae, Bembecidae, Crabronidae, Mellinidae, Pemphredonidae, Philanthidae, Psenidae, Sphecidae; see Fig. **1**; nodes **1**–**9**). By raising the nine former subfamilies (one subtribe) to family rank, we establish a natural system of Apoidea. Given the contradicting phylogenetic placement of the Heterogynaidae in our study and that by Branstetter et al. (2017)), we conservatively refrain from altering the taxonomic rank of the subfamily Heterogynaidae (e.g.*,* by treating it as subordinated group of Bembicidae) until more species and DNA sequence data of *Heterogyna* have been included in a phylogenetic analysis.

Since a phylogenetic system does not necessarily correspond with the traditional categories of the Linnaean hierarchy, conventions are required in order to handle newly inferred topologies, for example, those proposed by Wiley and Lieberman (2011). Specifically, the Linnaean system of biological nomenclature is in use since the eighteenth century and provides methods of arranging and ranking taxa to reflect their relative hierarchical position [55]. However, applying the Linnaean system of categories to a phylogeny might result in numerous problems. Considering the sister group relation of bees and Ammoplanina, we have to keep in mind that the clade representing the bees has no formally ranked name in the current classification. Some authors refer to this group as Apiformes [56] other as Anthophila [34]. However, this clade comprises various subordinated lineages that were granted taxonomic names of family rank (i.e.*,* Andrenidae, Apidae, Colletidae, Halictidae, Megachilidae, Melittidae, Stenotritidae).

Here, we propose to raise Ammoplanina from subtribe to family level. Although a Linnaean system of nomenclature seems not to be applicable in this case, Wiley and Lieberman (2011) proposed a modern way of integrating Linnaean nomenclature with phylogenetic studies by minimizing taxonomic decisions and changes to existing classifications. Applied to the present study, this suggests that the huge group of bees, having no formal ranked name, should be referred to as Anthophila due to their well-known special status. However, rising Ammoplanina to family rank (Ammoplanidae) is needed to ensure that the system remains encaptic (i.e.*,* that the sister group of a clade comprising multiple families does not hold a rank lower than that of a family). Our new classification of apoid wasps is thus minimally redundant, but maximally informative in respect of all newly proposed families [57]. As mentioned in the introduction, however, we were unable to study samples of Entomosericini, which are part of the former subfamily Pemphredoninae. The phylogenetic position of this lineage of apoid wasps consequently remains unclear. Until samples of Entomosericini are included in a future phylogenetic analysis, we suggest referring to this enigmatic lineage as Entomosericini *incertae sedis*.

## Conclusion

Our phylogenetic result, based on analyzing a combined enriched and transcriptomic dataset, allowed to trace the evolutionary origin of bees and revealed the Ammoplanina as possibly the closest extant relatives of bees, with an estimated divergence time of ca. 128 Mya (Late Cretaceous). The inferred phylogenetic relationships of apoid wasps suggests granting eight lineages of the former family “Crabronidae” (including Ammoplanina) family status in order to establish a natural classification of the superfamily Apoidea. The fact that both Ammoplanidae and the oldest known fossil bee, *Melittosphex burmensis*, exhibit a small body size (typically less than three millimeters; [38, 39]) suggests that the most recent common ancestor of bees likely also had a small body size. Intriguingly, species of Ammoplanidae are known to hunt thrips, of which some have been shown to visit flowers and to feed on pollen [58–60]. The ammoplanids’ biology thus indicates how the transition from a predatory to herbivorous (pollen-collecting) life style could have taken place. This insight plus the fact that all species of Apoidea exhibiting (eu-)social behavior evolved within a single subordinated lineage of this superfamily comprising pemphredonine and philanthine wasps as well as bees opens the door for future comparative studies that investigate what specific traits could have fostered the evolution of (eu-)sociality and what traits (e.g., changes in the digestive system) enabled the most recent common ancestor of bees to feed on pollen.

## Methods

### Bait design for target DNA enrichment

We used an early beta-version of the software BaitFisher to design target DNA enrichment baits from multiple nucleotide sequence alignments [29]. We used 24 transcript libraries of apoid wasp species compiled in context of the 1KITE project and released by Petersen et al. (2017) and the gene model of *Nasonia vitripennis* to infer 57,650,120-bp-long baits for capturing 282 single-copy genes and that OrthoDB 5 [61] suggested to be single-copy across all Hymenoptera. Based on the hierarchical clustering information stored in OrthoDB 7 [62] we later decided to rely on a more conservative estimate of which genes are likely single-copy across Hymenoptera (and selected outgroups) outlined by Peters et al. (2017). For further information on the bait design, see Mayer et al. (2016).

### Taxon sampling and genomic DNA extraction for target DNA enrichment

We selected 93 apoid wasp species to be included in our target enrichment based analysis comprising two species of Ampulicidae, one species of Heterogynaidae, twelve species of Sphecidae sensu stricto (*s.str*.) and 78 species of “Crabronidae” (Additional file 1: Table S4 and S5). We additionally included a representative of the bee family Stenotritidae, *Ctenocolletes rufescens*, the only extant bee family not included in the study of Peters et al. (2017), as well as a sample of the honeybee *Apis mellifera*. Please note that we already processed eight of the above mentioned 93 apoid wasp species in the study published by Mayer et al. (2016). The sampling covers almost all major subfamilies and tribes (except for the subfamily Eremiaspheciinae and the six tribes Aphelotomini, Bothynostethini, Entomosericini, Laphyragogini, and Xenosphecini) currently described in the catalog of Sphecidae sensu *lato* by Pulawski (2016). The fixative and the preservation time differed among the investigated samples: while most long-term stored samples had been preserved in 70% ethanol, most short-term stored samples had been preserved in 96% ethanol. Genomic DNA (gDNA) was extracted using the BioSprint 96 DNA extraction robot (Qiagen GmbH, Hilden, Germany) and following the BioSprint 96 DNA Plant Handbook. Due to differences in the preservation quality, collection date and size of the samples, we either dissected thorax muscles or used whole individuals for DNA extraction. We assessed the quality and quantity of the extracted gDNA with a Fragment Analyzer (Advanced Analytical Technologies GmbH, Heidelberg, Germany) and a Qubit 2.0 Fluorometer (Thermo Fisher, Waltham, USA). All DNA samples were stored at − 20 °C until further processing.

### Library preparation, target DNA enrichment and sequencing

When preparing DNA libraries for sequencing on a MiSeq v2 NGS platform (Illumina, San Diego, CA, USA), we followed (with minor modifications as outlined below) the TruSeq DNA Sample Preparation LT Kit A and B (Illumina, San Diego, CA, USA; protocol published in 2012) for multiplexed samples. Extracted gDNA was enzymatically sheared to fragments of 150–500 bp in length using the Next dsDNase Fragmentase Kit (New England Biolabs, Ipswich, USA). The subsequent library preparation steps comprising blunt-end repair, A-tailing, ligation with single-indexed adaptors and amplification of each prepared library followed the protocols given by Mayer et al. (2016). We assessed the quality and quantity of the amplified libraries with a Fragment Analyzer and a Qubit 2.0 Fluorometer. We pooled the indexed gDNA libraries with equal proportions into pools of four samples. Hybridization reaction of baits and post-capture sampling processing followed Agilent’s SureSelect Target Enrichment protocol for Illumina Multiplexed Sequencing, as described by Mayer et al. (2016). We assessed the quality and quantity of the captured libraries with a Fragment Analyzer and a Qubit 2.0 Fluorometer before paired-end sequencing each indexed sample pool on an Illumina MiSeq platform. Single-indexed DNA library pools were paired-end sequenced with a read length of 250-bp on an Illumina MiSeq platform. Raw reads were quality checked, trimmed, and de novo-assembled as described by Mayer et al. (2016). All contigs assembled from raw reads, sequenced on the same Illumina lane, were compared with each other to identify and remove possible cross-contaminant contigs as described by Mayer et al. (2016). To estimate the enrichment success, we used custom Perl scripts that calculated the coverage of target DNA regions as outlined by Mayer et al. (2016).

### Transcriptome data sampling

Given that transcripts of the here investigated target genes had been sequenced by Peters et al. (2017) in 167 species of Hymenoptera, we decided to include the corresponding DNA sequences of 85 selected species of Apoidea and outgroups. The 85 species comprised: Ampulicidae (2), Andrenidae (3), Apidae (16), Colletidae (2), “Crabronidae” (29), Halictidae (7), Megachilidae (9), Melittidae (3), Mutillidae (1), Pompilidae (1), Sapygidae (1), Scoliidae (2), Sphecidae (8), and Tiphiidae (1) (Additional file 1: Table S6).

### Orthology assessment of target genes in the enriched gDNA sequence libraries and in the transcript libraries

We used the software package Orthograph version 0.5.6 [63] (https://github.com/mptrsen/Orthograph/) and applied the same settings as described by Peters et al. (2017) to search the sequenced and assembled genomic DNA sequences as well as the transcript libraries for contigs of the investigated target genes (see Mayer et al. (2016) for details). While we initially designed baits to enrich 282 target genes, which OrthoDB 5 [61] suggested to be single-copy across all Hymenoptera [29], we later decided to rely on a more conservative estimate of which genes are likely single-copy across Hymenoptera (and selected outgroups) outlined by Peters et al. (2017) and based on the hierarchical clustering information stored in OrthoDB 7 [62]. The total number of target genes consequently dropped from 282 to 195. Since Orthograph searched the assembled genomic DNA sequences and the transcript libraries for the 195 target genes by exploiting the official gene sets of Hymenoptera with sequenced genome and whose nucleotide and amino acid sequences, we included the following species in our datasets: *Acromyrmex echinatior* (Official Gene Set (OGS) version 3.8) [64], *Apis mellifera* (OGS version 3.2) [65], *Camponotus floridanus* and *Harpegnathos saltator* (OGS version 3.3) [66]*, Nasonia vitripennis* (OGS version 2.0) [67], and *Tribolium castaneum* (OGS version 3.0) [68] (Additional file 1: Table S7). The amino acid sequences of the red flour beetle *Tribolium castaneum,* the only non-hymenopteran reference species, was only considered during the search for orthologs in the transcript and target enrichment libraries and were subsequently removed. In all amino acid sequences of target genes of the five reference species (i.e., *A*. *echinatior*, *A. mellifera*, *C. floridanus*, *H. saltator*, and *N. vitripennis*) with sequenced genome, as well as in translated sequences of the enriched target DNA, we searched for terminal as well as internal stop codons and Selenocycteine (U) residues. In the vast majority of stop codons were terminal ones. However, we also found a same internal stop codons that likely represent sequencing errors and/or assembly and concatenation (since Orthograph concatenates non-overlapping DNA sequences referring to the same ortholog group) artifacts. We removed all terminal stop codons and masked internal stop codons with ‘X’ and ‘NNN’ respectively.

### Data alignment

All identified single-copy orthologs were aligned on amino acid level using the multiple sequence alignment (MSA) program MAFFT version 7.123 using the L-INS-I alignment algorithm [69] described by Misof et al. (2014). The amino acid MSAs were further checked for potential misaligned sequences and subsequently refined as described by Misof et al. (2014). Remaining outlier sequences were removed from the respective amino acid MSAs and corresponding nucleotide sequence files. Given that we considered amino sequences of the non-aculeate parasitoid wasp *N. vitripennis* only to improve the identification of outlier sequences, we removed all amino acid and corresponding nucleotide sequence of this reference species after the alignment refinement and outlier removal procedure. The removal of sequences from a MSA can result in gap-only columns that we removed in the amino acid MSAs. Finally, we generated nucleotide sequence alignments using the amino acid MSAs as blue prints by applying a modified version of PAL2NAL version 14.1 [33, 70]. All MSAs have been deposited in and are available from MENDELEY Data (10.17632/3dh9k97jp8.1).

### Identification of protein domains

To improve the fit of the evolutionary substitution models onto the sequence data via data partitioning by facilitate partitioning of the amino acid and of the nucleotide sequence data based on protein domains and protein domain clans (i.e., evolutionary related protein domains), we searched the amino acid sequences of each MSA for protein domains contained in the Pfam A (release 28) [71] database with the aid of the software PfamScan version 1.5 (released 2013–10-15) [72] and HMMER version 3.1b2 [73] as outlined by Misof et al. (2014). All data block information has been deposited in and is available from MENDELEY Data (10.17632/3dh9k97jp8.1).

### Alignment masking and supermatrix generation

To improve the signal to-noise-ratio in the amino acid and the nucleotide MSAs, we evoke a modified version of the program Aliscore version 1.2 [74] on the amino acid level. Aliscore was told to respect gene boundaries and run with a default sliding window size, demanding evaluation of all possible pairwise comparisons and specifying that the analyzed MSAs resemble those inferred when analyzing gappy amino acid EST data (option -e). We merged the results from Aliscore (ambiguously aligned sites) and from the protein domain identification to concatenate data blocks and generate supermatrices consisting of protein-domain based data blocks, on both the amino acid and the nucleotide level as described by Misof et al. (2014). The information content of each single data block and of the complete dataset on amino acid level was calculated applying the software MARE version 0.1.2-rc [75]. To further optimize the amino acid and nucleotide dataset, we (**1**) removed all data blocks exhibiting no phylogenetic information content and (**2**) only kept data blocks that had at least one representative taxon of defined taxonomic groups we aimed to address (Additional file 1: Table S8). Specifically, we demanded each data block to contain sequence information of at least one representative species from each of the following groups: (1) outgroup; (2) bees; (3) Ammoplanina; (4) Pemphredonina + Spilomenina + Stigmina; (5) Psenini + Odontosphecini; (6) Philanthinae and all remaining species (7 in total) given in Additional file 1: Table S8. Given that the most recent study on Hymenoptera by Peters et al. (2017) proposed *Psenulus* as potential sister group to the bees – a representative of the “Psenini” a subgroup of the pemphredonin wasps – we focused on the origin of the bees when defining the above groups. We split all currently proposed sister groups of bees according to their phylogenetic status proposed by Peters et al. (2017). Intermediate results of all supermatrix processing steps on the amino acid and nucleotide level which we described above, have been deposited in and are available from MENDELEY Data (10.17632/3dh9k97jp8.1).

### Data partitioning and model selection

To find the best partitioning scheme and substitution model with respect to the protein domain-based data blocks, we used PartitionFinder version 2.0.0pre14 [72, 76, 77] in combination with RAxML version 8.2.8 [78] applying the corrected Akaike Information Criterion (AICc) and providing the above inferred data blocks. Following the suggestion by Misof et al. (2014), we started PartitionFinder by providing the protein domain based data blocks as a starting point for the meta-partition analysis. We first searched for a suitable partition scheme on the amino acid level considering the following substitution models LG + G, LG + G + F, WAG+G, WAG+G + F, BLOSUM62 + G, BLOSUM62 + G + F, DCMUT+G, DCMUT+G + F, JTT + G, JTT + G + F, LG4X. We started PartitionFinder with the following command line options: “--raxml --ml-tree -p 20 --weights 1,1,0,1 --rcluster-max 10000 --rcluster-percent 100 --all-states -min-subset-size 50”. In the configuration file, we specified the following settings: search [rcluster] (described by Lanfear et al. 2014), branchlengths [linked], model-selection [aicc], which enforces PartitionFinder to use the corrected Akaike information criterion (AICc) to decide whether or not data blocks are combined and which partition-specific model to apply. When analyzing the dataset on the nucleotide level, we also used the above inferred data blocks with corresponding coordinates in the nucleotide alignments as starting point for the PartitionFinder analysis. Furthermore, we applied two different settings (**1**) modeling each codon position within a given domain data block separately; the resulting partition scheme and model specification are subsequently referred to as PF-NT-1,2,3 and (**2**) modeling the first and second codon positions separately within each domain data block and removing third codon positions (the resulting partition scheme and model specification are subsequently referred to as PF-NT-1,2). Both PartitionFinder runs were started applying the same command line options and configuration file settings as we used when analyzing the data blocks on the amino acid level, except that we considered only the GTR + G nucleotide substitution model: GTR + G only. The final datasets and inferred partitioning files and schemes have been deposited in and are available from MENDELEY Data (10.17632/3dh9k97jp8.1).

### Phylogenetic analyses

We inferred phylogenetic trees using the maximum likelihood optimality criterion as implemented in the software Exascale Maximum Likelihood (ExaML) version 3.0.17 [79]. We analyzed three supermatrices, namely (**1**) amino acid level, (**2**) nucleotide level with 3rd codon position excluded (PF-NT-1,2), and (**3**) nucleotide level with all codon positions included (PF-NT-1,2,3). For each supermatrix, we conducted 50 separate tree searches, 25 using completely random starting trees and 25 using randomized stepwise addition parsimony starting trees. Per supermatrix, we selected the inferred phylogenetic tree with the best log-likelihood score from the 50 obtained phylogenetic estimates. Statistical node support was assessed via non-parametric bootstrapping from a total of 150 bootstrap replicates using ExaML version 3.0.17 [79]. The sufficiency of the number of bootstrap replicates was assessed a posteriori with the bootstrap convergence criterion implemented in RAxML version 8.2.8 [78, 80] (Weighted Robinson Fould distance building an extended majority-rule (MRE) consensus tree (autoMRE, threshold [0.03], with 1000 permutations). We selected the GTR-G model (option –m GTRGAMMA) when analyzing the data on the nucleotide level and ProtGAMMA-LG on the amino acid level applied the partition-specific substitution models inferred by PartitionFinder when analyzing the data on the amino acid level. A rate heterogeneity approximated by a gamma distribution was specified with the option –m PROTGAMMA. All inferred trees were rooted by selecting all non-apoid wasps as outgroup. The results from the phylogenetic analyses as well as of bootstrap analyses have been deposited in and are available from MENDELEY Data (10.17632/3dh9k97jp8.1).

To assess the sensitivity of our results on the applied phylogenetic inference method, we also conducted phylogenetic inferences in a Bayesian framework by using the software ExaBayes [81]. We applied the Bayesian approach to all three datasets described above: (**1**) amino acid dataset, (**2**) nucleotide dataset with 3rd codon position excluded (PF-NT-1,2) and (**3**) nucleotide dataset with all codon positions included (PF-NT-1,2,3). All analyses in the Bayesian framework resulted in identical tree topologies (Additional file 2: Figures S2.1, S3.1 and S4.1). ExaBayes was run as outlined by Bank et al. (2017) [82]. Specifically, each dataset was analyzed with three independent runs with 3,000,000 generation each. All runs converged with an average standard deviation of split frequencies (ASDSF) of 1.93% when analyzing the amino acid dataset, of 1.90% when analyzing the nucleotide dataset PF-NT-1,2, and of 3.19% when analyzing the nucleotide dataset PF-NT-1,2,3. Each consensus tree was built from three times 3,000,000 generations, sampled every 500 generations, and the first 25% samples were discarded (burn-in). In total we obtained 13,500 sampled trees from which posterior probability values were calculated.

To evaluate the possible impact of species, whose amino acid or DNA sequence evolution violated the assumptions of global stationary, reversibility, and homogeneity (SRH) [83, 84] in our datasets, we furthermore conducted pairwise sequence comparisons using Bowker’s matched-pairs test of symmetry [85]. We generated heat maps based on inferred *p*-values using SymTest version 2.0.47 (https://github.com/ottmi/symtest) and described by Bank et al. (2017). Bowker’s test was applied on the following two datasets: (**1**) amino acid dataset and (**2**) nucleotide dataset with all codon positions included (PF-NT-1,2,3).

We applied Four-cluster Likelihood Mapping (FcLM), as described by Misof et al. (2014) and Bank et al. (2017), to assess whether or not conflicting and/or confounding signal influence the phylogenetic position of specific lineages (i.e., Ammoplanina and Mellininae) in our study. Specifcially, we tested the relationships of **a**) Ammoplanina (two species), Psenini (four species) + Odontosphecini (one species), Anthophila (42 species), and all remaining species in our dataset (including outgroup species) to each other (Hypothesis 1), and of **b**) Mellininae (one species), Sphecidae (19 species), Crabroninae (39 species) + Dinetinae (one species), and all remaining species in our dataset (including outgroup species) to each other (Hypothesis 2) (Additional file 3: Table 1). FcLM was done with ExaML version 3.0.17 [79] on the original amino acid supermatrix and on the nucleotide supermatrix with all codon positions included, using parsimony start trees and applying the partitioning scheme and substitution models as described above. For conducting the permutation tests, we used the same software and partitioning scheme as when analyzing the original supermatrices, but we replaced the original supermatrix with randomized data inferred with the aid of the LG substitution matrix (LG substitution model was applied across all partitions when analyzing the permutation matrices via FcLM). Results were visualized in simplex graphs using customized Perl scripts. The FcLM results have been deposited in and are available from MENDELEY Data (10.17632/3dh9k97jp8.1).

### Rogue taxon analysis

We identified species with variable positions among the trees reconstructed during bootstrap analyses of the amino acid and of the two nucleotide datasets with the software RogueNaRok version 1.0 [86]. The following set of parameters were chosen: (**1**) three different values of the consensus threshold for bipartitions in the bootstrap tree set (option -c 50 [*MR default*]; −c 75 and -c 100 [*strict*]); (**2**) identification of rogue taxa that affect the support in the (greedily) extended majority rule (option -c MRE); (**3**) mapping bipartition support of bootstrap trees onto the maximum likelihood estimate (MLE) tree; (**4**) pruning sets of two or more taxa (i.e., dropsets) at a time to investigate the improvement of the consensus tree support (option -s). Finally, the identified rogue taxa were pruned from the bootstrap trees to improve the bootstrap support. A reduced consensus tree was inferred using RAxML version 8.2.8 [78].

### Divergence time estimation

We estimated divergence times within a Bayesian framework [31] with the software MCMCtree, which is part of the PAML software package version 4.9 [30]. We first calculated maximum likelihood estimates (MLEs) of branch lengths and the Hessian matrix (option: usedata [3]). The matrix and the branch length were then used to calculate the posterior probability of divergence times using Markov Chain Monte Carlo (MCMC) sampling and applying a relaxed-clock model (usedata [2]; clock [2]; cleandata [0]; Bdpas [1;1;0]; kappa_gamma [6;2]; alpha_gamma [1;1]; rgene_gamma [2;2]; sigma2_gamma [1;10]; finetune [1;0.1;0.1;0.1;0.01;0.5]). The chosen time unit was 100 million years and the selected substitution model was REV (GTR). We ran MCMCtree two times independently for 100,000 iterations and discarded the first 10,000 iterations as burn-in. Since we lacked fossils to calibrate the root of the tree, we specified the maximum age of the root as being less than 179 Ma (RootAge [< 1.79]). This value was inferred by Peters et al. (2017) in the currently most comprehensive analysis of the evolutionary history of Hymenoptera as the age of the last common ancestor of extant “Vespoidea” (sensu) [35] and Apoidea. The Bayesian estimation of species divergence times was inferred from analyzing 284,607 nucleotide sites and applying the protein domain-based partition scheme considering all three codon positions (PF-NT-1,2,3). We considered ten fossils as calibration points and used them to specify minimal node ages with soft bounds (i.e., we allowed the node age to be younger with a 2.5% probability) in the rooted tree inferred from analyzing the nucleotide dataset PF-NT-1,2,3 (Additional file 1: Table S9). Chosen fossils were selected following the best-practice recommendations [87]. We ran the dating analysis four times with the same settings to verify that different runs produce similar results and we plotted the inferred estimates against each other to highlight possible differences (Additional file 2: Figure S5). The results from the four independent species divergence estimations have been deposited in and are available from MENDELEY Data (10.17632/3dh9k97jp8.1).

## Additional files

Additional file 1: **Table S1.** Assembly statistics and number of identified single-copy genes in the analyzed enriched data set. **Table S2.** Assembly statistics and number of identified single-copy genes in the analyzed transcriptomes. **Table S3.** Taxa with unstable phylogenetic position (a.k.a. rogue taxa) when analyzing the amino acid and the nucleotide supermatrices. **Table S4.** Families, subfamilies and tribes included in this study. **Table S5**. Detailed species list used for target DNA enrichment, including information on identity, sex and concentration of extracted genomic DNA. **Table S6.** Published transcriptomes of apoid wasps and bees included in the present investigation. **Table S7.** Official gene sets exploited to identify orthologous transcripts and enriched DNA of target single-copy protein-coding genes. **Table S8.** Information on required taxa when removing data blocks with poor taxonomic coverage. **Table S9**. Description, origin, phylogenetic position and age of fossils used to calibrate divergence times. **Table S10.** Four-cluster Likelihood (FcLM) results on amino acid and nucleotide level when testing the phylogenetic placement of Ammoplanina and Mellininae**.** Proportions of quartets, that map to specific areas in the 2D-simplex graph. (PDF 230 kb)

Additional file 2: **Figure S1.** Ultrametric and time-calibrated tree of Apoidea estimated using a relaxed molecular clock approach as implemented in MCMCtree. The estimates are based on the analysis of 284,607 nucleotide sites and applying a combination of protein domain – and codon-based partitioning scheme by modelling 1st, 2nd and 3rd codon positions separately. **Figure S2.** Phylogenetic relationships of Apoidea. The phylogenetic tree was inferred from analyzing 94,869 amino acid sites under the maximum likelihood (ML) optimality criterion. The data matrix was partitioned based on a protein domain-based partitioning scheme and analyzed with partition-specific substitution models. Node labels indicate bootstrap branch support values derived from 150 bootstrap replicates. **Figure S2.1.** Phylogenetic relationships of Apoidea. The phylogenetic tree was inferred with ExaBayes, by analyzing 94,869 amino acid sites. The data matrix was partitioned based on a protein domain-based partitioning scheme and analyzed with partition-specific substitution models automatically selected by ExaBayes. Posterior probability values were inferred from a total of 13,500 sampled trees. **Figure S3.** Phylogenetic relationships of Apoidea. The phylogenetic tree was inferred from analyzing 284.607 nucleotide sites under the maximum likelihood (ML) optimality criterion. The data matrix was partitioned based on applying a combination of protein domain – and codon-based partitioning scheme by modelling 1st and 2nd codon positions separately and excluding the 3rd codon positions. Each partition was analyzed with the partition-specific model parameters under the nucleotide substitution model GTR + G. Node labels indicate bootstrap branch support values derived from 150 bootstrap replicates. **Figure S3.1.** Phylogenetic relationships of Apoidea. The phylogenetic tree was inferred with ExaBayes, by analyzing 284.607 nucleotide sites. The data matrix was partitioned based on applying a combination of protein domain – and codon-based partitioning scheme by modelling 1st and 2nd codon positions separately and excluding the 3rd codon positions. Each partition was analyzed with the partition-specific model parameters under the nucleotide substitution model GTR + G. Posterior probability values were inferred from a total of 13,500 sampled trees. **Figure S4**. Phylogenetic relationships of Apoidea. The phylogenetic tree inferred from analyzing 284.607 nucleotide sites under the maximum likelihood (ML) optimality criterion. The data matrix was partitioned based on applying a combination of protein domain – and codon-based partitioning scheme by modelling the 1st, 2nd and 3rd codon position separately. Each partition was analyzed with partition-specific model parameters under the nucleotide substitution model GTR + G. Node labels indicate bootstrap branch support values derived from 100 bootstrap replicates. **Figure S4.1**. Phylogenetic relationships of Apoidea. The phylogenetic tree was inferred with ExaBayes, by analyzing 284.607 nucleotide sites. The data matrix was partitioned based on applying a combination of protein domain– and codon-based partitioning scheme by modelling the 1st, 2nd and 3rd codon position separately. Each partition was analyzed with partition-specific model parameters under the nucleotide substitution model GTR + G. Posterior probability values were inferred from a total of 13,500 sampled trees. **Figure S5.** Comparison of divergence times and confidence intervals from four independent dating analyses conducted from with MCMCtree. Differences in the results hint at differences in the convergence of the MCMC method. The closer the dots are to the angle bisector, the more similar the estimates are for the two runs that are compared. **Figure S6**. Results from Bowker’s matched-pairs test of symmetry. Heat maps showing the results from pairwise comparison of aligned a) amino acid dataset and b) nucleotide dataset with all three codon positions included (PF-NT-1,2,3). White cells specify *p*-values > 0.05, indicating that corresponding pairs of nucleotide or amino acid sequences do not violate the assumption of global stationary, reversibility, and homogeneity (SRH) conditions. (PDF 1011 kb)

Additional file 3: **Table 1.** Information on grouping of species in a given quartet when assessing the phylogenetic position of Ammoplanina (two species) and Mellininae (one species) from analyzing the amino acid supermatrix and nucleotide supermatrix including all three codon positions via Four-cluster Likelihood Mapping (FcLM). (PDF 652 kb)

Additional file 4: **Figure 2.** Results from the Four-cluster Likelihood Mapping showing the support for the possible relationship of Ammoplanina (two species), Psenini (four species) + Odontosphecini (one species), Anthophila (42 species) and all remaining species including outgroup species to each other (58050 quartets). Original amino acid supermatrix (94,869 amino acid sites) based on a protein domain-based partitioning scheme and analyzed with partition-specific substitution models (a), permutation scheme I with supermatrix as in a, but amino acids are permuted within partitions while retaining the specific distribution of missing data (b), permutation scheme II with supermatrix as in b, but replacing amino acids in each partition with randomly selected amino acids, using amino acid frequencies as given by the LG substitution matrix, while retaining the specific distribution of missing data (c) and permutation scheme III supermatrix as given in c, but with missing data being randomly permuted (d). **Figure 3.** Results from the Four-cluster Likelihood Mapping showing the support for the possible relationship of Mellininae (one species), Sphecidae (19 species), Crabroninae (39 species) + Dinetinae (one species), and all remaining species including bees and outgroup species to each other (103320 quartets). Original amino acid supermatrix (94,869 amino acid sites) based on a protein domain-based partitioning scheme and analyzed with partition-specific substitution models (a), permutation scheme I with supermatrix as in a, but amino acids are permuted within partitions while retaining the specific distribution of missing data (b), permutation scheme II with supermatrix as in b, but replacing amino acids in each partition with randomly selected amino acids, using amino acid frequencies as given by the LG substitution matrix, while retaining the specific distribution of missing data (c) and permutation scheme III supermatrix as given in c, but with missing data being randomly permuted (d). **Figure 4.** Results from the Four-cluster Likelihood Mapping showing the support for the possible relationship of Ammoplanina (two species), Psenini (four species) + Odontosphecini (one species), Anthophila (42 species) and all remaining species including outgroup species to each other (58050 quartets). Original nucleotide supermatrix (284.607 nucleotide sites) partitioned based on applying a combination of protein domain – and codon-based partitioning scheme by modelling the 1st, 2nd and 3rd codon position separately. Each partition was analyzed with partition-specific model parameters under the nucleotide substitution model GTR+G (a), permutation scheme I with supermatrix as in a, but nucleotides are permuted within partitions while retaining the specific distribution of missing data (b), permutation scheme II with supermatrix as in b, but replacing nucleotides in each partition with randomly selected nucleotides, while retaining the specific distribution of missing data (c) and permutation scheme III supermatrix as given in c, but with missing data being randomly permuted (d). **Figure 5.** Results from the Four-cluster Likelihood Mapping showing the support for the possible relationship of Mellininae (one species), Sphecidae (19 species), Crabroninae (39 species) + Dinetinae (one species), and all remaining species including bees and outgroup species to each other (103320 quartets). Original nucleotide supermatrix (284.607 nucleotide sites) partitioned based on applying a combination of protein domain – and codon-based partitioning scheme by modelling the 1st, 2nd and 3rd codon position separately. Each partition was analyzed with partition-specific model parameters under the nucleotide substitution model GTR+G (a), permutation scheme I with supermatrix as in a, but nucleotides are permuted within partitions while retaining the specific distribution of missing data (b), permutation scheme II with supermatrix as in b, but replacing nucleotides in each partition with randomly selected nucleotides, while retaining the specific distribution of missing data (c) and permutation scheme III supermatrix as given in c, but with missing data being randomly permuted (d). (DOC 1269 kb)

## Abbreviations

AICc: Akaike information criterion
BS: Bootstrap support
CI: Confidence interval
ExaML: Exascale maximum likelihood
FcLM: Four-cluster Likelihood Method
gDNA: Genomic DNA
ML: Maximum likelihood
MLE: Maximum likelihood estimate
MRE: Extended majority-rule
MSA: Multiple sequence alignment
Mya: Million years ago
OGS: Official gene set
*s.str*.: Sensu stricto
SRH: Global stationary, reversibility, and homogeneity
