# Supplementary material for: Phylogenomic analysis of Apoidea sheds new light on the sister group of bees
Source: BMC Evol Biol. 2018 May 18;18:71. doi: 10.1186/s12862-018-1155-8 (PMC5960199; doi:10.1186/s12862-018-1155-8)
Supplement: Supplementary file 1 — Table S1. Assembly statistics and number of identified single-copy genes in the analyzed enriched data set. Table S2. Assembly statistics and number of identified single-copy genes in the analyzed transcriptomes. Table S3. Taxa with unstable phylogenetic position (a.k.a. rogue taxa) when analyzing the amino acid and the nucleotide supermatrices. Table S4. Families, subfamilies and tribes included in this study. Table S5. Detailed species list used for target DNA enrichment, including information on identity, sex and concentration of extracted genomic DNA. Table S6. Published transcriptomes of apoid wasps and bees included in the present investigation. Table S7. Official gene sets exploited to identify orthologous transcripts and enriched DNA of target single-copy protein-coding genes. Table S8. Information on required taxa when removing data blocks with poor taxonomic coverage. Table S9. Description, origin, phylogenetic position and age of fossils used to calibrate divergence times. Table S10. Four-cluster Likelihood (FcLM) results on amino acid and nucleotide level when testing the phylogenetic placement of Ammoplanina and Mellininae. Proportions of quartets, that map to specific areas in the 2D-simplex graph. (PDF 230 kb) [file 12862_2018_1155_MOESM1_ESM.pdf]

Additional table S1: Assembly statistics and number of identified single-copy genes in the analyzed enriched data set. Asterisks indicate information inferred data after the cross contamination check. DB7/5 = OrthoDB 7/5 database; Ct = average base-coverage depth of those contigs that contain a 250-bp-long bait-binding sequence section[1]; bp = base pair.

		contigs														
**Species name**	**Number of raw reads**	**Number of contigs**	**Number of cleaned contigs**	**Assembly size [bp]***	**Size of smallest contig [bp]***	**Size of largest contig [bp]***	**N50 [bp]***	**GC content [%]***	**Number of identified orthologs from a total of 3260 (DB7)***	**Number of enriched identified orthologs* from a total of 195 (DB5)**	**Captured genes [%]**	**Number of on-target contigs**	**Number of off-target contigs**	**Length of contigs referring to target DNA [MIN-MDN-MAX]**	**Read count per contigs referring to target DNA [MIN-MDN-MAX]**	**average base-coverage depth of contigs referring to target genes (Ct)**
Ampulex compressa	298.491	7.926	7.733	3.879.827	200	7.646	501,7	47,2	362	167	85,6	253	7.480	329-821-2659	185-8392-50868	2546,1
Dolichurus sp.	226.633	11.985	11.655	4.740.862	200	5.505	406,4	44,0	282	94	48,2	452	11.213	204-398-1090	91-753-214003	472,6
Ammophila sp.	1.626.101	76.369	76.216	47.699.203	200	10.799	625,8	48,1	1,747	177	90,8	2.650	73.566	233-667-3958	48-908-617669	340,3
Chalybion sp. I	1.596.111	23.783	22.758	10.729.715	200	6.236	471,5	55,2	272	162	83,1	335	22.423	448-524-1522	246-3050-92899	1455,1
Chalybion sp. II	1.848.529	14.637	13.531	6.398.305	200	8.702	472,9	53,5	242	159	81,5	304	13.227	319-655-8501	43-5341-67661	2038,5
Chlorion sp.	190.484	6.001	6.001	2.050.839	200	2.775	341,7	53,4	191	114	58,5	276	5.725	234-312-2622	200-494-111205	395,8
Dynatus burmeisteri	689.653	14.814	7.261	3.171.194	200	7.177	436,7	48,2	316	156	80,0	270	6.991	328-588-1717	398-5712-28286	2429
Eremnophila melanaria	190.536	3.768	1.658	808.381	200	3.888	487,6	51,1	231	162	83,1	276	1.382	327-614-1686	369-3035-12435	1235,7
Palmodes sp.	601.200	64.787	61.677	32.255.187	200	5.794	523,0	46,7	1,461	177	90,8	2.187	59.490	252-504-4602	46-758-371108	375,9
Penepodium fumipenne	508.112	23.770	23.467	11.575.416	200	5.705	493,1	52,1	644	159	81,5	838	22.638	221-481-2605	145-760-208595	395,0
Podalonia sp.	532.385	16.704	10.800	5.535.012	200	2.580	504,9	46,5	387	137	70,3	464	10.893	237-494-2434	64-900-135648	455,5
Prionyx subatratus	1.029.603	2.521	2.020	950.004	202	21.974	470,3	47,9	157	146	74,9	183	1.837	282-505-21854	166-1783-1401236	860,4
Sphex funerarius	197.196	3.495	3.203	1.542.082	201	6.056	481,4	45,3	220	162	83,1	278	2.925	383-757-2456	1452-760-30,806	251
Stangeella cyaniventris	861.856	9.016	4.495	2.033.019	200	4.491	452,3	49,7	190	146	74,9	251	4.244	308-553-2674	377-2756-16,766	1245,9
Acanthostethus sp.	638.206	7.882	6.977	3.247.258	200	4.429	465,4	44,8	264	158	81,0	318	6.659	260-689-2429	116-7746-69932	2810,6
Ammatomus sp. II	444.329	9.454	8.821	4.128.556	200	4.596	468,0	49,9	232	158	81,0	343	8.478	243-617-2628	123-5586-79100	2263,4
Ammatomus sp. I	67.065	3.478	3.152	1.310.525	200	5.705	415,8	45,1	85	75	38,5	103	3.049	210-442-1220	179-1005-4503	568,4
Ammoplanellus sp.	624.709	9.150	8.718	4.122.280	200	4.463	472,8	49,8	251	128	65,6	301	8.417	217-494-1675	65-1501-41070	759,6
Ammoplanus sp.	1.408.454	33.434	32.761	16.958.833	200	6.018	517,7	48,8	720	161	82,6	1.068	31.693	235-477-2289	74-593-109705	310,8
Anacrabro sp.	814.513	19.888	19.341	10.221.369	200	8.678	528,5	47,9	646	166	85,1	752	18.589	232-575-3433	111-863-304933	375,2
Arigorytes sp.	247.230	7.357	7.042	1.998.403	120	8.616	283,8	44,7	114	87	44,6	233	6.809	122-390-8416	32-980-199415	628,2
Astata sp. I	1.565.172	8.119	7.242	4.207.389	203	11.772	581,0	47,1	183	119	61,0	230	7.012	206-544-2820	96-1968-467136	904,4
Astata sp. II	1.823.507	30.463	30.109	16.089.844	100	5.795	534,4	51,0	426	107	54,9	539	29570	252-544-3206	63-1129-140394	518,8
Belomicrus sp.	333.013	3.950	3.602	1.544.928	200	4.924	428,9	47,4	239	156	80,0	283	3.319	299-525-1647	122-2095-22509	997,6
Bembecinus sp.	602.360	41.237	40.991	20.814.921	200	5.968	507,8	44,5	872	166	85,1	1.267	39.727	201-526-3502	144-809-270300	384,5
Bicyrtes ventralis	560.776	24.117	23.159	10.401.406	200	8.442	449,1	48,9	468	168	86,2	558	22.601	308-596-3111	93-885-186545	371,2
Brachystegus sp.	1.637.919	29.332	28.880	15.139.206	200	5.845	524,2	44,3	857	171	87,7	1.282	27.598	209-495-3078	24-959-257225	484,3
Carinostigmus sp.	436.462	3.605	3.641	1.584.070	200	4.170	457,7	48,6	179	144	73,8	218	3.243	203-516-2014	179-2661-23028	1289,2
Cerceris acanthophila	707.573	11.849	10.800	5.452.604	200	5.280	504,9	46,5	268	160	82,1	319	10.481	231-495-2098	134-1754-35858	885,8
Cerceris arenaria	2.494.696	35.385	35.039	17.981.058	200	4.917	513,2	48,4	510	165	84,6	757	34.282	225-529-3015	119-1598-200660	755,2
Clypeadon sculleni	833.770	13.035	12.876	5.936.219	200	6.454	461,0	42,0	303	159	81,5	262	12.605	333-803-1967	207-7325-31294	2280,5
Clypeadon taurulus	779.083	17.179	16.782	8.486.180	200	6.261	505,7	46,1	305	155	79,5	437	16.345	319-579-2733	91-2695-209847	1163,6
Crabro scutellatus	1.796.274	54.479	52.878	26.497.746	200	9.315	501,1	51,1	1,14	172	88,2	1.666	51.212	222-486-3529	51-600-532011	308,6
Dasyproctus sp.	219.655	10.541	10.295	5.036.894	200	8.560	489,1	49,9	264	147	75,4	314	9.981	297-605-1743	130-3088-81245	1276,0
Didineis sp.	437.696	6.034	3.535	1.634.637	201	6.666	462,4	47,5	101	80	41,0	119	3.416	264-437-6466	91-1430-26103	818,1
Dryudella sp.	266.998	3.526	2.428	1.162.538	210	4.616	478,8	48,0	82	70	35,9	100	2.328	232-453-1550	78-772-5156	426,0
Ectemnius sonorensis	947.114	24.155	23.069	11.219.431	200	4.449	486,3	50,3	559	162	83,1	1.074	21.995	200-447-2299	68-562-166922	314,3
Entomognathus sp.	1.001.394	29.041	28.650	13.576.164	200	4.846	473,9	48,2	882	162	83,1	1.279	27.371	204-410-2892	107-633-138744	385,9
Eucerceris arenaria	507.621	7.601	7.339	3.352.956	204	5.879	456,9	48,5	222	149	76,4	252	7.087	299-562-2943	92-2518-54036	1120,1
Foxita bara ssp. *patei* I	380.720	3.451	2.537	1.296.325	200	5.150	511,0	46,4	159	92	47,2	213	2.324	205-528-2631	85-3161-65196	1496,7
Foxita bara ssp. *patei* II	1.198.060	20.716	18.964	10.209.311	200	3.918	538,4	53,8	168	41	21,0	202	18.762	299-516-2409	113-607-86422	294,1
Gorytes albidulus	795.653	5.124	4.288	2.290.756	200	8.856	534,2	46,1	198	124	63,6	290	3.998	204-561-8654	96-3324-159028	1481,3
Heliocausus sp.	575.718	932	539	269.260	207	21.915	499,6	48,2	22	11	5,6	22	517	234-385-21795	142-659-834935	427,9
Holotachysphex sp.	703.920	5.023	3.854	1.789.839	200	5.352	464,4	54,8	164	128	65,6	210	3.644	278-548-1740	38-2343-34751	1068,9
Hoplisoides sp.	357.234	29.725	29.343	16.574.552	200	8.257	564,9	49,6	974	172	88,2	1.262	28.081	230-555-4086	62-751-397480	338,3
Larra sp.	903.930	14.458	13.596	7.018.935	202	7.002	516,3	54,3	339	148	75,9	420	13.176	320-612-3312	51-1454-143531	593,9
Larropsis sp.	884.880	10.976	9.196	4.071.695	120	10.318	442,8	54,1	204	113	57,9	223	8.973	131-656-2205	63-2553-46574	972,9
Lestica alata	1.985.322	6.945	6.232	3.007.383	200	7.773	482,6	48,0	276	155	79,5	354	5.878	200-553-2424	49-3875-275262	1751,8
Lindenius panzeri	899.427	9.377	6528	2.319.373	120	8.630	355,3	47,6	151	36	18,5	171	6.357	140-452-8404	98-775-174796	428,7
Liogorytes sp.	212.391	2.151	1.827	801.962	100	10.529	439,0	48,8	167	151	77,4	195	1.632	284-580-2303	177-2496-24802	1075,9
Liris sp.	1.904.632	21.549	21.220	9.692.379	200	8.178	456,8	55,7	389	145	74,4	460	20.760	249-510-3343	83-2003-315804	981,9
Lyroda sp.	933.611	12.202	11.010	5.424.679	200	8.868	492,7	52,4	346	154	79,0	437	10.573	215-599-3073	100-1594-42395	665,3
Microbembex sp.	2.950.168	74.757	72.581	42.327.119	200	6.838	583,2	47,2	1,633	175	89,7	3.341	69.240	200-509-5248	57-542-321919	266,2
Microstictia hurdi	1.691.804	19.500	19.500	9.317.991	200	8.653	477,8	46,5	387	165	84,6	737	18.763	204-475-7128	50-956-199913	503,1
Mimesa lutaria	1.028.170	14.731	13.822	6.615.783	200	4.567	478,6	47,4	413	166	85,1	596	13.226	200-484-2450	55-1704-82828	880,2
Moniaecera sp.	1.325.523	22.073	15.890	7.553.284	200	8.179	475,3	48,7	444	130	66,7	712	15.178	201-463-3071	89-706-1901415	381,2
Neodasyproctus sp. I	2.111.231	25.335	21.326	10.178.710	200	10.610	477,3	44,1	231	72	36,9	330	20.996	204-504-10410	83-1046-336183	518,8
Neodasyproctus sp. II	1.339.059	1.049	510	345.009	216	10.512	676,5	47,3	51	18	9,2	56	454	329-566-10301	238-1028-3779446	454,1
Odontosphex paradoxus	1.027.010	19.801	18.948	9.736.304	200	7.418	513,8	48,4	367	155	79,5	433	18.515	320-608-3034	60-2632-312254	1082,2
Paranysson sp.	3.408.960	51.878	50.629	26.298.713	200	5.934	519,4	51,8	1,356	172	88,2	2.218	48.411	214-519-3314	36-670-283199	322,7
Philanthus gibbosus	560.608	7.597	7.161	3.416.237	201	6.494	477,1	42,2	200	162	83,1	234	6.927	332-714-3183	124-4545-54990	1591,4
Pison sp.	497.572	11.061	10.648	5.671.998	200	3.269	532,7	51,3	356	159	81,5	416	10.187	319-617-3069	109-2099-99404	850,5
Polemistus sp.	229.102	1.578	1.395	724.000	220	3.811	519,0	47,7	149	135	69,2	175	1.220	321-508-1712	136-1374-8767	676,2
Prosopigastra nearctica	514.774	8.725	6.460	3.220.358	207	14.699	498,5	55,9	156	72	36,9	172	6.288	319-556-2148	45-994-122067	446,9
Psammaecius versicolor	1.017.023	18.104	17.339	9.266.645	200	6.054	534,4	50,9	502	166	85,1	725	16.614	249-581-4472	74-1347-178595	579,6
Psenulus dilectus	541.998	5.132	1.493	679.492	212	5.440	455,1	49,6	112	90	46,2	130	1.363	320-412-1192	26-819-5398	496,9
Psenulus sp.	766.608	12.931	12.070	4.393.029	120	34.632	364,0	47,6	282	160	82,1	326	11.744	188-544-2173	44-3762-92718	1728,9
Pseudoplisus (Gorytes) willcoxi	885.170	5.397	3.611	1.848.493	204	22.063	511,9	45,9	188	105	53,8	209	3.402	235-622-21863	92-4112-131879	1512,1
Pseudoscolia sp.	2.035.494	35.162	34.449	19.455.571	200	6.311	564,8	45,7	846	169	86,7	1.622	32.827	200-504-3321	39-915-312689	453,9
Quexua sp.	2.738.682	52.461	50.418	27.582.662	200	6.807	547,1	52,0	1,085	166	85,1	1.686	48.732	202-513-2975	24-522-327343	254,4
Rhopalum clavipes	2.204.328	9.569	6.673	1.966.500	120	14.718	294,7	47,8	185	142	72,8	271	6.402	120-453-14518	60-3505-806304	1934,3
Rubrica nasuta	404.623	4.108	7.269	3.420.972	200	4.423	470,6	47,5	338	154	79,0	177	7.092	319-430-1156	130-498-33513	289,5
Selman notatus	357.725	6.658	6.328	3.067.205	200	3.232	484,7	48,5	209	157	80,5	263	6.065	323-688-3032	57-9345-113108	3395,7
Sphecius spectabilis	722.805	23.938	22.730	10.191.725	200	9.512	448,4	46,9	491	171	87,7	579	22.151	241-640-3360	85-1164-246353	454,7
Sphodrotes sp.	1.242.697	21.280	20.237	10.774.849	200	8.686	532,4	53,7	531	161	82,6	820	19.417	202-548-3869	65-1208-126721	551,1
Steniolia duplicata	109.486	5.800	5.448	2.507.101	200	8.624	460,2	44,9	232	159	81,5	278	5.170	256-616-1895	39-8771-65200	3559,7
Stictia heros	1.124.412	19.101	19.091	9.096.523	200	8.256	476,2	44,7	492	167	85,6	279	18.822	209-781-2953	224-260672-1452367	83441,7
Stictiella sp.	610.219	32.272	31.476	17.951.521	200	9.665	570,3	44,2	762	168	86,2	984	30.492	221-546-3263	59-821-362975	375,9
Stigmus sp.	2.143.963	22.843	21.966	11.121.968	200	4.985	506,3	50,1	563	160	82,1	873	21.093	232-488-2667	27-1078-165306	552,3
Stizoides foxi	1.611.511	16.405	15.855	8.722.656	203	6.073	550,2	49,0	475	164	84,1	639	15.216	263-581-3603	18-932-282908	401,0
Stizus aff marthae	883.142	14.822	13.749	6.517.334	200	8.041	474,0	46,9	271	161	82,6	314	13.435	308-784-3354	84-16288-221713	5193,9
Stizus vespoides	216.555	13.268	12.596	5.067.947	200	5.705	402,3	45,9	243	161	82,6	293	12.303	216-622-1930	210-6909-58595	2776,9
Synnevrus sp.	706.567	10.171	9.077	4.762.364	200	4.723	524,7	46,0	292	162	83,1	400	8.677	322-598-2628	96-3507-79984	1488,5
Tachysphex sp.	2.435.288	20.187	19.240	10.450.877	200	9.798	543,2	53,4	396	166	85,1	541	18.699	311-567-3319	1-1965-161699	754,6
Tachytes amazonus	482.169	10.730	10.528	5.198.737	200	10.104	493,8	54,0	263	146	74,9	327	10.201	253-622-2668	75-1876-132003	754,0
Tachytes sp.	2.435.288	33.788	33.126	16.903.874	200	6.638	510,3	55,9	585	160	82,1	776	32.350	311-569-3319	111-1214-231030	533,4
Tanyoprymnus moneduloides	1.160.206	4.869	2.327	888.921	200	7.183	382,0	47,0	15	13	6,7	15	2.312	319-418-753	55-583-2162	348,7
Tanyoprymnus sp.	481.848	19.067	18.757	9.331.326	200	8.673	497,5	48,5	368	157	80,5	418	18.339	319-686-3432	129-1661-169443	605,3
Tiguipa sp.	1.546.326	38.049	36.977	18.784.718	200	6.292	408,0	49,2	873	166	85,1	1.563	35.414	201-460-2755	40-464-141049	252,2
Trachypus sp.	541.478	2.843	2.445	1.228.151	219	10.942	502,3	45,1	184	161	82,6	203	2.242	323-593-3139	373-4059-47796	1711,2
Trichosticta sp.	1.458.316	25.805	24.821	11.638.172	200	8.851	468,9	43,8	593	170	87,2	978	23843	206-488-3066	43-337-185933	172,6
Trypoxylon (Trypargilum) lactitarse	591.922	7.823	7.360	3.794.955	204	4.461	515,6	50,2	252	155	79,5	294	7.066	256-656-3257	134-2012-59536	766,8
Heterogyna nocticola	426.766	1166	268	111.249	211	5.478	415,1	48,8	9	7	3,6	10	258	325-399-526	135-501-1057	313,9
Apis mellifera II	244.393	2.191	2.120	876.716	233	4.422	413,5	45,5	148	114	58,5	188	1.932	319-414-1070	307-2880-14881	1739,1
Ctenocolletes rufescens	1.051.714	29.196	28.827	14.059.979	200	4.463	487,7	53,7	359	131	67,2	487	28.340	200-492-2812	85-972-183510	493,9
**min**	**67.065**	**932**	**268**	**111.249**	**100**	**2.580**	**284**	**42,0**	**1,085**	**7**	**4**	**10**	**258**			**172,6**
**ave**	**980.955**	**17.365**	**16.287**	**8.287.459**	**195**	**7.652**	**484**	**48,6**	**314,8**	**139**	**71**	**567,6**	**15693,3**			**966,9**
**med**	**779.083**	**12.931**	**11.010**	**5.424.679**	**200**	**6.454**	**486**	**48,2**	**264**	**157**	**81**	**326,5**	**10733**			**616,75**
**max**	**3.408.960**	**76.369**	**76.216**	**47.699.203**	**233**	**34.632**	**677**	**55,9**	**974**	**177**	**91**	**3341**	**73566**			**5193,9**

Additional table S2: Assembly statistics and number of identified single-copy genes in the analyzed transcriptomes. Gene recovery for the transcriptomic data set when using Orthograph version 0.5.6.

Species name	Assembly size [bp]	Number of contigs	Size of smallest contig [bp]	Size of largest contig [bp]	N50 [bp]	GC content [%]	Number of identified orthologs (strict)	Number of identified orthologs (relaxed)	Number of identified orthologs used for target enrichment
Alysson spinosus	47.574.924	40.545	200	21.220	3.134	43,4	2.021	2.577	194
Ammobates syriacus	32.110.512	22.137	200	18.369	2.789	40,7	1.957	2.395	192
Ammophila sabulosa	27.191.551	21.852	200	19.285	2.216	46,5	1.988	2.373	188
Ampulex fasciata	21.476.963	24.883	200	16.932	1.274	41,1	1.300	1.542	176
Andrena vaga	29.428.733	25.569	200	15.777	2.133	43,4	2.003	2.496	192
Anthidium manicatum	27.880.352	22.793	200	37.472	2.044	40,8	2.091	2.538	190
Anthophora plumipes	30.778.652	29.661	200	30.310	1.812	39,2	2.010	2.445	191
Astata minor	36.656.229	31.832	200	23.055	2.146	44,3	2.090	2.574	194
Bembix rostrata	37.839.804	33.341	200	20.100	2.075	39,0	1.930	2.325	190
Bombus rupestris	36.654.470	30.548	200	44.845	2.183	38,3	2.164	2.713	195
Camptopoeum sacrum	32.757.317	23.988	200	29.013	2.474	39,9	2.041	2.524	191
Ceratina chalybea	27.820.342	17.439	200	18.982	2.825	42,9	1.977	2.421	193
Cerceris arenaria	34.252.864	24.719	200	27.246	2.623	40,3	2.074	2.573	165
Chalybion californicum	33.977.878	21.323	200	33.401	3.091	42,6	1.878	2.322	191
Chelostoma florisomne	34.076.628	21.800	200	38.028	3.000	38,1	2.117	2.639	193
Chlorion hirtum	36.650.475	22.147	200	32.363	3.302	43,1	1.942	2.473	194
Coelioxys conoidea	33.319.440	31.666	200	29.937	1.835	38,5	2.077	2.554	192
Colletes cunicularius	35.031.277	32.942	200	29.986	1.981	40,5	2.082	2.624	192
Colpa sexmaculata	34.941.997	26.456	200	31.332	2.484	40,7	1.933	2.345	189
Crabro peltarius	27.834.096	17.809	200	16.863	2.886	44,1	1.903	2.321	187
Crossocerus quadrimaculatus	27.666.906	16.344	200	19.322	3.058	45,0	1.968	2.412	191
Dasypoda hirtipes	29.908.505	22.722	200	28.960	2.342	39,4	2.101	2.562	191
Dinetus pictus	35.261.360	20.195	200	27.202	3.438	45,1	2.023	2.531	192
Diodontus minutus	39.373.028	22.820	200	27.275	3.432	43,3	2.157	2.723	195
Dioxys cincta	44.526.242	29.227	200	28.657	3.015	38,0	2.089	2.644	193
Dolichurus corniculus	27.844.668	19.309	200	14.238	2.745	39,3	1.961	2.367	186
Dryudella pinguis	30.800.832	19.086	200	17.147	3.089	44,4	1.994	2.449	190
Dufourea dentiventris	27.874.588	35.243	200	22.057	1.342	39,6	1.823	2.185	184
Epeolus variegatus	29.366.577	23.624	200	31.763	2.115	39,9	2.005	2.429	190
Eucera nigrescens	29.687.969	32.735	200	34.135	1.394	38,6	1.538	1.815	177
Eucera plumigera	26.851.933	25.706	200	20.428	1.882	39,9	1.144	1.456	102
Eucera syriaca	28.054.992	27.204	200	21.300	1.789	39,0	1.268	1.560	123
Euglossa dilemma	40.223.997	31.917	200	51.296	2.423	39,8	2.129	2.704	191
Gorytes laticinctus	30.119.789	20.336	200	27.853	2.781	44,1	1.992	2.432	193
Halictus quadricinctus	32.852.514	24.065	200	20.508	2.520	41,0	2.012	2.441	193
Harpactus elegans	35.499.888	22.245	200	27.045	3.310	45,7	1.975	2.428	193
Heriades truncorum	35.349.951	23.340	200	34.139	2.804	40,1	2.106	2.670	193
Hylaeus variegatus	27.792.085	22.760	200	16.898	2.082	39,9	1.839	2.212	188
Lasioglossum xanthopus	35.788.897	25.042	200	16.569	2.614	40,8	2.039	2.492	190
Lestica clypeata	19.306.384	15.702	200	19.154	2.007	41,2	1.618	1.923	187
Liris sp	28.840.977	17.615	200	17.729	2.991	43,9	1.880	2.269	189
Lithurgus chrysurus	32.731.326	24.312	200	33.878	2.397	39,1	2.079	2.514	191
Macropis fulvipes	27.205.906	21.091	200	30.318	2.262	40,7	1.960	2.346	191
Megachile willughbiella	36.153.965	29.038	200	33.703	2.289	39,3	2.122	2.636	192
Melitta haemorrhoidalis	20.788.457	26.035	200	12.759	1.197	41,3	1.493	1.750	185
Mellinus arvensis	30.450.447	21.642	200	17.911	2.575	44,8	1.997	2.423	192
Nitela sp	73.133.740	54.668	200	18.051	2.430	43,3	2.101	2.607	194
Nomada lathburiana	35.759.640	24.014	200	17.457	2.779	41,0	2.047	2.616	194
Nomia diversipes	29.177.935	21.953	200	19.744	2.299	39,8	2.066	2.473	192
Nomioides sp	28.076.986	31.377	200	15.473	1.575	40,8	1.814	2.161	185
Nysson niger	29.091.955	22.496	200	24.856	2.294	39,7	1.977	2.384	188
Osmia cornuta	32.383.868	30.007	200	29.116	1.916	40,4	2.066	2.591	194
Oxybelus bipunctatus	37.187.137	22.233	200	32.289	3.447	40,5	1.968	2.525	195
Palarus histrio	28.994.684	17.033	200	26.925	3.226	45,1	1.865	2.248	191
Panurgus dentipes	26.437.230	23.066	200	31.383	1.925	40,3	1.970	2.375	190
Passaloecus eremita	33.770.894	25.059	200	32.145	2.475	40,5	2.036	2.458	190
Pemphredon lugens	39.425.911	24.675	200	28.097	3.266	41,3	2.079	2.625	193
Philanthus triangulum	27.356.021	21.729	200	17.443	2.225	38,3	1.898	2.257	190
Pison atrum	26.691.364	24.840	200	20.599	1.865	44,5	1.860	2.236	187
Podalonia hirsuta	32.136.789	21.108	200	21.318	2.768	47,6	1.996	2.444	192
Pompilus cinereus	36.212.051	19.489	200	27.429	3.583	41,7	2.007	2.457	191
Prionyx kirbii	31.095.319	21.703	200	29.684	2.670	43,1	1.954	2.373	190
Psenulus fuscipennis	31.095.907	24.423	200	22.011	2.239	39,4	2.111	2.596	193
Pseudoscolia sinaitica	28.843.716	22.577	200	26.252	2.376	40,2	1.929	2.309	191
Sapyga quinquepunctata	33.296.307	22.097	200	27.542	2.745	46,0	2.049	2.572	191
Sceliphron curvatum	32.739.311	22.934	200	18.683	2.614	45,7	1.934	2.402	190
Scolia hirta	38.046.022	28.080	200	19.699	2.548	41,9	1.956	2.416	191
Smicromyrme rufipes	31.191.065	31.152	200	16.628	1.607	39,1	1.998	2.400	191
Sphecius convallis	25.618.375	19.967	200	21.226	2.247	42,4	1.799	2.155	188
Sphecodes albilabris	36.915.692	33.273	200	39.999	1.996	42,9	2.101	2.646	192
Sphex funerarius	37.503.328	26.189	200	32.973	2.921	43,3	1.962	2.477	192
Spilomena beata	31.845.961	24.104	200	24.678	2.386	41,4	2.040	2.466	192
Stelis punctulatissima	39.729.802	28.380	200	37.726	2.649	40,1	2.175	2.745	195
Stizoides tridentatus	34.689.230	27.724	200	22.267	2.487	42,5	1.907	2.285	189
Stizus continuus	24.816.142	21.045	200	18.531	1.970	40,6	1.806	2.144	192
Systropha curvicornis	34.780.634	34.917	200	20.911	1.819	39,9	1.967	2.383	195
Tachysphex fulvitarsis	32.940.922	17.308	200	28.002	3.712	44,7	1.860	2.329	192
Tetragonula carbonaria	48.214.225	88.297	200	18.096	707	41,0	1.801	2.218	188
Tetralonia malvae	36.479.504	31.360	200	42.751	2.051	39,7	1.975	2.403	188
Tetraloniella nigriceps	29.157.534	25.917	200	19.976	2.187	40,1	1.493	1.841	139
Tetraloniella sp	26.307.232	27.649	200	38.242	1.623	39,9	1.355	1.662	128
Thyreus orbatus	39.591.151	26.321	200	31.904	2.889	40,3	2.036	2.566	194
Tiphia femorata	36.053.834	24.784	200	18.719	2.814	40,4	2.002	2.509	194
Trypoxylon figulus	31.639.291	19.169	200	15.729	3.245	41,7	1.928	2.386	191
Xylocopa violacea	28.673.767	21.588	200	27.956	2.338	41,3	2.099	2.600	190
**min**	19.306.384	15.702	200	12.759	707	38,0	1.144	1.456	102
**ave**	32.772.673	25.853	200	25.521	2.437	41,4	1.940	2.382	187
**med**	32.136.789	24.065	200	24.856	2.423	40,8	1.988	2.429	191
**max**	73.133.740	88.297	200	51.296	3.712	47,6	2.175	2.745	195

Additional table S3: List of species that showed rogue behavior in the phylogenetic analyses. c: consensus threshold; s: dropset size. MRE: majority rule consensus tree.

supermatrix	c	s	Rogue taxa
amino acid	50	2	*Heterogyna nocticola, Lindenius panzeri*
		3	*Heterogyna nocticola, Lindenius panzeri*
		4	*Heterogyna nocticola, Lindenius panzeri*
	75	2	*Heterogyna nocticola, Lindenius panzeri, Neodasyproctus* sp. II
		3	*Heterogyna nocticola, Lindenius panzeri, Neodasyproctus* sp. II
		4	*Heterogyna nocticola, Lindenius panzeri, Neodasyproctus* sp. II
	100	2	*Heterogyna nocticola, Lindenius panzeri, Neodasyproctus* sp. II, *Tanyoprymnus moneduloides*
		3	*Heterogyna nocticola, Lindenius panzeri, Neodasyproctus* sp. II, *Tanyoprymnus moneduloides*
		4	*Heterogyna nocticola, Lindenius panzeri, Neodasyproctus* sp. II, *Tanyoprymnus moneduloides*
	MRE	2	*Heterogyna nocticola, Lindenius panzeri*
		3	*Heterogyna nocticola, Lindenius panzeri*
		4	*Heterogyna nocticola, Lindenius panzeri*
PF-NT-1,2 nucleotide (1st and 2nd codon position)	50	2	*Heterogyna nocticola, Microstictia hurdi*
		3	*Heterogyna nocticola, Microstictia hurdi*
		4	*Heterogyna nocticola, Microstictia hurdi*
	75	2	*Heterogyna nocticola, Lindenius panzeri, Quexua* sp., *Tanyoprymnus moneduloides*
		3	*Heterogyna nocticola, Lindenius panzeri, Quexua* sp., *Tanyoprymnus moneduloides*
		4	*Heterogyna nocticola, Lindenius panzeri, Quexua* sp., *Tanyoprymnus moneduloides*
	100	2	*Heterogyna nocticola, Tanyoprymnus moneduloides, Lindenius panzeri, Dryudella* sp., *Neodasyproctus* sp. II
		3	*Heterogyna nocticola, Tanyoprymnus moneduloides, Lindenius panzeri, Dryudella* sp., *Neodasyproctus* sp. II
		4	*Heterogyna nocticola, Tanyoprymnus moneduloides, Lindenius panzeri, Dryudella* sp., *Neodasyproctus* sp. II
	MRE	2	*Heterogyna nocticola*
		3	*Heterogyna nocticola*
		4	*Heterogyna nocticola*
PF-NT-1,2,3 nucleotide (1st, 2nd, and 3rd codon position)	50	2	*Heterogyna nocticola, Lindenius panzeri, Tetraloniella* sp., *Microstictia hurdi*
		3	*Heterogyna nocticola, Lindenius panzeri,Tetraloniella* sp., *Microstictia hurdi*
		4	*Heterogyna nocticola, Lindenius panzeri,Tetraloniella* sp., *Microstictia hurdi*
	75	2	*Heterogyna nocticola, Lindenius panzeri,Tetraloniella* sp., *Microstictia hurdi*, *Neodasyproctus* sp. II
		3	*Heterogyna nocticola, Lindenius panzeri,Tetraloniella* sp., *Microstictia hurdi*, *Neodasyproctus* sp. II
		4	*Heterogyna nocticola, Lindenius panzeri,Tetraloniella* sp., *Microstictia hurdi*, *Neodasyproctus* sp. II
	100	2	*Heterogyna nocticola, Lindenius panzeri, Pompilus cinereus, Tanyoprymnus moneduloides*
		3	*Heterogyna nocticola, Lindenius panzeri, Pompilus cinereus, Tanyoprymnus moneduloides*
		4	*Heterogyna nocticola, Lindenius panzeri, Pompilus cinereus, Tanyoprymnus moneduloides*
	MRE	2	*Heterogyna nocticola*
		3	*Heterogyna nocticola*
		4	*Heterogyna nocticola, Nomada lathburiana, Ammobates syriacus, Tetraloniella nigriceps, Tetraloniella* sp.

Additional table S4: List of families, subfamilies, and tribes of apoid wasps included in the present investigation. Shown is the traditional classification of apoid wasp as given in the Catalog of Sphecidae *senu lato* by Pulawski (2016). Bold numbers indicate the total number of described groups as listed by Pulawski (2016); numbers in parentheses indicate the number of species of a given taxonomic group included in this study. * samples of the subfamily Eremiaspheciinae were unavailable to us; ** samples of the tribe Aphelotomini were unavailable to us; *** samples of the tribes Bothynostethini, Entomosericini, Eremiaspheciini, Laphyragogini, and Xenosphecini were unavailable to us.

**Family**	**Subfamilies**	**Tribes**
Heterogynaidae	N/A	N/A
Ampulicidae	**2** (2)	**3** (2)**
Sphecidae *s.str.*	**4** (4)	**5** (5)
Crabronidae	**8** (7)*	**23** (18)***

Additional table S5: Detailed list of species included in the target DNA enrichment. Listed are information about the sampled data, sampling site and date as well as the quantity of extracted genomic DNA. Taxonomic classification according to W. J. Pulawski (2016).We included two bees and the following apoid wasps: two Ampulicidae, 12 Sphecidae *s.str.*,78 Crabronidae and one Heterogynaidae.

Family	Subfamily	Tribe	Subtribe	Species	preservation date	preservation material	collection place	male / female	Amount of extracted DNA [ng/µl]
Ampulicidae		Ampulicini		*Ampulex compressa*	August 2013	96 % ethanol	Düsseldorf, Germany	m	3,1
Ampulicidae	Dolichurinae			*Dolichurus* sp.	22.-29.10.2007	96 % ethanol	Madagaskar	m	0,8
Sphecidae *s.str.*	Ammophilinae			*Ammophila* sp.	August 2013	96 % ethanol	Arizona, SW-USA	m	8,1
Sphecidae *s.str.*	Ammophilinae			*Eremnophila melanaria*	January 2006	70 % ethanol	Argentina, South America	m	7,76
Sphecidae *s.str.*	Ammophilinae			*Podalonia* sp.	February 2013	96 % ethanol	Nicaragua, Central America	m	55,0
Sphecidae *s.str.*	Chloriontinae			*Chlorion* sp.	August 2013	96 % ethanol	Arizona, SW-USA	m	16,8
Sphecidae *s.str.*	Sceliphrinae	Podiini		*Dynatus burmeisteri*	January 2006	70 % ethanol	Argentina, South America	m	6,28
Sphecidae *s.str.*	Sceliphrinae	Podiini		*Penepodium fumipenne*	unknown date	70 % ethanol	unknown origin	f	5,2
Sphecidae *s.str.*	Sceliphrinae	Sceliphrini		*Chalybion* sp. I	February 2013	96 % ethanol	Nicaragua, Central America	m	26,9
Sphecidae *s.str.*	Sceliphrinae	Sceliphrini		*Chalybion* sp. II	February 2013	96 % ethanol	Nicaragua, Central America	m	28,2
Sphecidae *s.str.*	Sphecinae	Prionychini		*Palmodes* sp.	August 2012	96 % ethanol	Arizona, SW-USA	m	14,5
Sphecidae *s.str.*	Sphecinae	Prionychini		*Prionyx subatratus*	August 2013	96 % ethanol	Arizona, SW-USA	m	5,29
Sphecidae *s.str.*	Sphecinae	Sphecini		*Sphex funerarius* II	August 2003	70 % ethanol	Isl.of Rhodos, Greece	m	14,0
Sphecidae *s.str.*	Sphecinae	Stangeellini		*Stangeella cyaniventris*	January 2006	70 % ethanol	Argentina, South America	m	30,9
Crabronidae	Astatinae			*Astata* sp. I	23.04.2013	96 % ethanol	Israel ON_3882	m	0,42
Crabronidae	Astatinae			*Astata* sp. II	August 2013	96 % ethanol	Arizona, SW-USA	m	15,2
Crabronidae	Astatinae			*Dryudella* sp.	August 2012	96 % ethanol	Arizona, SW-USA	m	1,13
Crabronidae	Bembicinae	Alyssontini		*Didineis* sp.	01.07.2013	96 % ethanol	Israel ON_5374	m	5,56
Crabronidae	Bembicinae	Bembicini	Bembicina	*Bicyrtes ventralis*	August 2012	96 % ethanol	Arizona, SW-USA	m	11,5
Crabronidae	Bembicinae	Bembicini	Bembicina	*Microbembex* sp.	February 2013	96 % ethanol	Nicaragua, Central America	m	16,1
Crabronidae	Bembicinae	Bembicini	Bembicina	*Rubrica nasuta*	January 2006	70 % ethanol	Argentina, South America	m	9,7
Crabronidae	Bembicinae	Bembicini	Bembicina	*Selman notatus*	January 2006	70 % ethanol	Argentina, South America	m	24,5
Crabronidae	Bembicinae	Bembicini	Bembicina	*Stictia heros*	February 2013	96 % ethanol	Nicaragua, Central America	f	92,0
Crabronidae	Bembicinae	Bembicini	Bembicina	*Trichosticta* sp..	May 2011	70 % ethanol	Peru, South America	m	22,8
Crabronidae	Bembicinae	Bembicini	Gorytina	*Arigorytes* sp.	June 2003	70 % ethanol	California, USA	f	5,69
Crabronidae	Bembicinae	Bembicini	Gorytina	*Gorytes albidulus*	July 2013	96 % ethanol	Brandenburg, Germany	m	3,3
Crabronidae	Bembicinae	Bembicini	Gorytina	*Pseudoplisus (Gorytes) willcoxi*	August 2012	96 % ethanol	Arizona, SW-USA	m	4,91
Crabronidae	Bembicinae	Bembicini	Gorytina	*Hoplisoides* sp.	August 2013	96 % ethanol	Arizona, SW-USA	m	7,7
Crabronidae	Bembicinae	Bembicini	Gorytina	*Liogorytes* sp.	January 2006	70 % ethanol	Argentina, South America	m	10,9
Crabronidae	Bembicinae	Bembicini	Gorytina	*Psammaecius versicolor*	26.05.2012	96 % ethanol	Israel ON_2531	m	24,4
Crabronidae	Bembicinae	Bembicini	Spheciina	*Ammatomus* sp. I	May 2009	70 % ethanol	Thailand T4828	m	4,0
Crabronidae	Bembicinae	Bembicini	Spheciina	*Ammatomus* sp. II	May 2009	70 % ethanol	Thailand T4828	m	26,9
Crabronidae	Bembicinae	Bembicini	Spheciina	*Sphecius spectabilis*	January 2006	70 % ethanol	Argentina, South America	m	13,6
Crabronidae	Bembicinae	Bembicini	Spheciina	*Tanyoprymnus moneduloides*	August 2001	70 % ethanol	Arizona, SW-USA	m	22,8
Crabronidae	Bembicinae	Bembicini	Spheciina	*Tanyoprymnus* sp.	August 2013	96 % ethanol	Arizona, SW-USA	m	1,2
Crabronidae	Bembicinae	Bembicini	Stictiellina	*Microstictia hurdi*	August 2007	70 % ethanol	Arizona, SW-USA	m	28,2
Crabronidae	Bembicinae	Bembicini	Stictiellina	*Steniolia duplicata*	August 2012	96 % ethanol	Arizona, SW-USA	m	14,8
Crabronidae	Bembicinae	Bembicini	Stictiellina	*Stictiella* sp.	August 2013	96 % ethanol	Arizona, SW-USA	m	11,5
Crabronidae	Bembicinae	Bembicini	Stizina	*Bembecinus* sp.	February 2013	96 % ethanol	Nicaragua, Central America	f	10,6
Crabronidae	Bembicinae	Bembicini	Stizina	*Stizoides foxi*	August 2012	96 % ethanol	Arizona, SW-USA	m	11,5
Crabronidae	Bembicinae	Bembicini	Stizina	*Stizus aff marthae*	May 2012	96 % ethanol	Israel ON_2530	m	10,9
Crabronidae	Bembicinae	Bembicini	Stizina	*Stizus vespoides*	May 2012	96 % ethanol	Israel ON_2528	m	32,4
Crabronidae	Bembicinae	Heliocausina	Heliocausina	*Heliocausus* sp.	January 2006	70 % ethanol	Argentina, South America	m	1,21
Crabronidae	Bembicinae	Heliocausina	Heliocausina	*Tiguipa* sp.	January 2006	70 % ethanol	Argentina, South America	f	16,1
Crabronidae	Bembicinae	Nyssonini	Nyssonina	*Acanthostethus* sp.	15.10.2014	96 % ethanol	QL, Australien	m	21,9
Crabronidae	Bembicinae	Nyssonini	Nyssonina	*Brachystegus* sp.	October 2008	96 % ethanol	Madagascar	m	7,87
Crabronidae	Bembicinae	Nyssonini	Nyssonina	*Synnevrus* sp.	March 2009	70 % ethanol	Thailand	m	17,4
Crabronidae	Crabroninae	Crabronini	Anacrabronina	*Anacrabro* sp.	January 2006	70 % ethanol	Argentina, South America	m	9,73
Crabronidae	Crabroninae	Crabronini	Anacrabronina	*Entomognathus* sp.	01.07.2013	96 % ethanol	Israel ON_5421	m	0,38
Crabronidae	Crabroninae	Crabronini	Crabronina	*Crabro scutellatus*	June 2004	70 % ethanol	Schwedt, Germany	m	16,3
Crabronidae	Crabroninae	Crabronini	Crabronina	*Dasyproctus* sp.	May 2008	96 % ethanol	Madagascar	m	9,4
Crabronidae	Crabroninae	Crabronini	Crabronina	*Ectemnius sonorensis*	August 2012	96 % ethanol	Arizona, SW-USA	f	19,7
Crabronidae	Crabroninae	Crabronini	Crabronina	*Foxita bara* ssp. *patei* I	September 2012	96 % ethanol	Arizona, SW-USA	f	8,02
Crabronidae	Crabroninae	Crabronini	Crabronina	*Foxita bara* ssp. *patei* II	August 2012	96 % ethanol	Arizona, SW-USA	f	14,7
Crabronidae	Crabroninae	Crabronini	Crabronina	*Lestica alata*	July 2013	96 % ethanol	Brandenburg; Germany	m	3,97
Crabronidae	Crabroninae	Crabronini	Crabronina	*Lindenius panzeri*	July 2013	96 % ethanol	Brandenburg; Germany	m	3,99
Crabronidae	Crabroninae	Crabronini	Crabronina	*Moniaecera* sp.	August 2013	96 % ethanol	Arizona, SW-USA	f	2,2
Crabronidae	Crabroninae	Crabronini	Crabronina	*Neodasyproctus* sp. I	October 2008	96 % ethanol	Madagascar	m	1,54
Crabronidae	Crabroninae	Crabronini	Crabronina	*Neodasyproctus* sp. II	October 2008	96 % ethanol	Madagascar	m	1,51
Crabronidae	Crabroninae	Crabronini	Crabronina	*Quexua* sp.	August 2012	96 % ethanol	Ecuador, South America	f	17,3
Crabronidae	Crabroninae	Crabronini	Crabronina	*Rhopalum clavipes*	August 2012	96 % ethanol	Arizona, SW-USA	f	2,14
Crabronidae	Crabroninae	Larrini	Gastrosericina	*Holotachysphex* sp.	January 2009	96 % ethanol	Madagaskar	f	1,54
Crabronidae	Crabroninae	Larrini	Gastrosericina	*Larropsis* sp.	August 2013	96 % ethanol	Arizona, SW-USA	m	1,16
Crabronidae	Crabroninae	Larrini	Gastrosericina	*Prosopigastra nearctica*	24.08.2007	70 % ethanol	Arizona, SW-USA	f	2,71
Crabronidae	Crabroninae	Larrini	Gastrosericina	*Tachysphex* sp.	12.10.2014	96 % ethanol	NSW, Australien	m	0,86
Crabronidae	Crabroninae	Larrini	Gastrosericina	*Tachytes amazonus*	January 2006	96 % ethanol	Argentina, South America	m	1,87
Crabronidae	Crabroninae	Larrini	Gastrosericina	*Tachytes* sp.	12.08.2013	96 % ethanol	Arizona, SW-USA	m	7,77
Crabronidae	Crabroninae	Larrini	Larrina	*Larra* sp.	February 2013	96 % ethanol	Nicaragua, Central America	m	1,41
Crabronidae	Crabroninae	Larrini	Larrina	*Liris* sp. II	January 2009	70 % ethanol	Thailand (T4288)	f	4,41
Crabronidae	Crabroninae	Miscophini		*Lyroda* sp.	11.10.2014	96 % ethanol	NSW, Australia	f	0,15
Crabronidae	Crabroninae	Miscophini		*Paranysson* sp.	01.09.2012	96 % ethanol	Gabon	f	0,33
Crabronidae	Crabroninae	Miscophini		*Sphodrotes* sp.	13.10.2014	96 % ethanol	NSW, Australia	f	2,98
Crabronidae	Crabroninae	Oxybelini		*Belomicrus* sp.	August 2013	96 % ethanol	Arizona, SW-USA	m	0,38
Crabronidae	Crabroninae	Trypoxylini		*Pison* sp.	15.10.2014	96 % ethanol	QL, Australien	m	0,97
Crabronidae	Crabroninae	Trypoxylini		*Trypoxylon (Trypargilum) lactitarse*	February 2013	96 % ethanol	Nicaragua, Central America	f	1,35
Crabronidae	Pemphredoninae	Odontosphecini		*Odontosphex paradoxus*	January 2006	70 % ethanol	Argentina, South America	m	1,18
Crabronidae	Pemphredoninae	Pemphredonini	Ammoplanina	*Ammoplanellus* sp.	November 2008	96 % ethanol	Madagaskar MG 54B 03	m	0,3
Crabronidae	Pemphredoninae	Pemphredonini	Ammoplanina	*Ammoplanus* sp.	October 2008	96 % ethanol	Madagaskar	m	0,47
Crabronidae	Pemphredoninae	Pemphredonini	Pemphredonina	*Polemistus* sp.	August 2012	96 % ethanol	Arizona, SW-USA	m	0,12
Crabronidae	Pemphredoninae	Pemphredonini	Stigmina	*Stigmus* sp.	June 2012	70 % ethanol	Ecuador	m	0,42
Crabronidae	Pemphredoninae	Pemphredonini	Stigmina	*Carinostigmus* sp.	5.-12.3.2009	70 % ethanol	Thailand Khuean Srinagarindra NP (T4778)	f	0,67
Crabronidae	Pemphredoninae	Psenini		*Mimesa lutaria*	July 2013	96 % ethanol	Brandenburg, Germany	f	0,51
Crabronidae	Pemphredoninae	Psenini		*Psenulus dilectus*	24.-31.12.2007	96 % ethanol	Madagaskar	m	0,13
Crabronidae	Pemphredoninae	Psenini		*Psenulus* sp.	August 2013	96 % ethanol	California, SW-USA	m	0,56
Crabronidae	Philanthinae	Aphilanthopini		*Clypeadon sculleni*	August 2012	96 % ethanol	Arizona, SW-USA	m	29,8
Crabronidae	Philanthinae	Aphilanthopini		*Clypeadon taurulus*	August 2012	96 % ethanol	Arizona, SW-USA	m	10,9
Crabronidae	Philanthinae	Cercerini		*Cerceris acanthophila*	August 2012	96 % ethanol	Arizona, SW-USA	m	0,26
Crabronidae	Philanthinae	Cercerini		*Cerceris arenaria*	July 2013	96 % ethanol	Brandenburg, Germany	m	2,69
Crabronidae	Philanthinae	Cercerini		*Eucerceris arenaria*	August 2007	70 % ethanol	Arizona, SW-USA	m	3,86
Crabronidae	Philanthinae	Philanthini		*Philanthus gibbosus*	August 2012	96 % ethanol	Arizona, SW-USA	m	1,82
Crabronidae	Philanthinae	Philanthini		*Trachypus* sp.	February 2013	96 % ethanol	Nicaragua, Central America	f	5,24
Crabronidae	Philanthinae	Pseudoscoliini		*Pseudoscolia* sp.	May 2012	96 % ethanol	Israel	m	2,5
Heterogynaidae				*Heterogyna nocticola*	December 2003	70 % ethanol	Oman, Arabian Peninsula	m	0,57
Apidae (*sensu lato*)	Apidae			*Apis mellifera* II	August 2013	96 % ethanol	Halle, Germany	m	142
Apidae (*sensu lato*)	Stenotritidae			*Ctenocolletes rufescens*	09.03.2011	96 % ethanol	Australia, Credo Station	m	17,4

Additional table S6: List of species whose transcriptomic data (1KITE) was embedded in the enriched dataset. Published transcriptomes of apoid wasps and bees included in the study of Peters *et al.* (2017). Taxonomic classification of apoid wasps according to W. J. Pulawski (2016).

Assembly file (e3; TSA accepted)					species name
130314_I269_FCC1KFEACXX_L7_INSobdTCVRAAPEI-41.tsa.fas	Ampulicidae	Ampulicinae	Ampulicini		*Ampulex fasciata*
130125_I266_FCC1MY6ACXX_L3_INSntgTAFRAAPEI-221.tsa.fas	Ampulicidae	Dolichurinae			*Dolichurus corniculus*
110817_I809_FCD05CDACXX_L3_INSbusTBNRABPEI-121.tsa.fas	Andrenidae				*Andrena vaga*
130919_I247_FCC2V7VACXX_L1_INSofmTCNRAAPEI-41.tsa.fas	Andrenidae				*Camptopoeum sacrum*
130206_I238_FCC1LVUACXX_L1_INSlupTAMRAAPEI-74.tsa.fas	Andrenidae				*Panurgus dentipes*
110817_I809_FCD05CDACXX_L3_INSbusTBGRABPEI-127.tsa.fas	Apidae				*Anthophora plumipes*
120429_I266_FCC0HG0ACXX_L7_INSytvTATRAAPEI-46.tsa.fas	Apidae				*Bombus rupestris*
120707_I249_FCD111GACXX_L4_INSswpTARRAAPEI-11.tsa.fas	Apidae				*Ceratina chalybea*
120707_I249_FCD111GACXX_L3_INSswpTBBRAAPEI-21.tsa.fas	Apidae				*Epeolus variegatus*
130728_I263_FCD23HKACXX_L2_INSofmTASRAAPEI-84.tsa.fas	Apidae				*Eucera nigrescens*
130919_I247_FCC2V7VACXX_L6_INSinlTAORAAPEI-32.tsa.fas	Apidae				*Eucera plumigera*
130919_I247_FCC2V7VACXX_L6_INSinlTAXRAAPEI-55.tsa.fas	Apidae				*Eucera syriaca*
120316_I251_FCC0HJ1ACXX_L8_INStmbTANRAAPEI-89.tsa.fas	Apidae				*Euglossa dilemma*
110817_I809_FCD05CDACXX_L3_INSbusTBQRAAPEI-82.tsa.fas	Apidae				*Nomada lathburiana*
121030_I251_FCC19KWACXX_L1_INSeqtTDERAAPEI-75.tsa.fas	Apidae				*Tetragonula carbonaria*
120707_I249_FCD111GACXX_L4_INSswpTAYRAAPEI-18.tsa.fas	Apidae				*Tetralonia malvae*
131012_I246_FCC2J5BACXX_L8_INSinlTAFRAAPEI-61.tsa.fas	Apidae				*Tetraloniella nigriceps*
131012_I246_FCC2J5BACXX_L8_RINSinlTDKRAAPEI-118.tsa.fas	Apidae				*Tetraloniella sp*
120215_I277_FCD0KP1ACXX_L7_INSjdsTAORAAPEI-44.tsa.fas	Apidae				*Thyreus orbatus*
110817_I809_FCD05CDACXX_L3_INSbusTBORAAPEI-80.tsa.fas	Apidae				*Xylocopa violacea*
130728_I263_FCD23HKACXX_L1_INSofmTACRAAPEI-35.tsa.fas	Apidae				*Ammobates syriacus*
110817_I809_FCD05CDACXX_L3_INSbusTBARABPEI-119.tsa.fas	Colletidae				*Colletes cunicularius*
120707_I249_FCD111GACXX_L2_INSswpTAMRAAPEI-30.tsa.fas	Colletidae				*Hylaeus variegatus*
121010_I249_FCD1C4BACXX_L6_INSeqtTAQRAAPEI-12.tsa.fas	Crabronidae	Astatinae			*Astata minor*
130919_I247_FCC2V7VACXX_L7_RINSinlTBHRAAPEI-136.tsa.fas	Crabronidae	Astatinae			*Dryudella pinguis*
120429_I266_FCC0HG0ACXX_L8_INSytvTBDRAAPEI-9.tsa.fas	Crabronidae	Bembicinae	Alyssontini		*Alysson spinosus*
120521_I249_FCC0U4RACXX_L7_INSytvTBERAAPEI-11.tsa.fas	Crabronidae	Bembicinae	Bembicini	Gorytina	*Gorytes laticinctus*
120707_I249_FCD111GACXX_L3_INSswpTAFRAAPEI-16.tsa.fas	Crabronidae	Bembicinae	Bembicini	Gorytina	*Harpactus elegans*
120126_I283_FCD0L80ACXX_L2_INSnfrTBORAAPEI-14.tsa.fas	Crabronidae	Bembicinae	Bembecini	Spheciina	*Sphecius convallis*
120429_I266_FCC0HG0ACXX_L8_INSytvTARRAAPEI-44.tsa.fas	Crabronidae	Bembicinae	Bembecini	Stizina	*Stizoides tridentatus*
130112_I269_FCC1M19ACXX_L2_INSqiqTAGRAAPEI-55.tsa.fas	Crabronidae	Bembicinae	Bembecini	Stizina	*Stizus continuus*
120707_I249_FCD111GACXX_L4_INSswpTBNRAAPEI-44.tsa.fas	Crabronidae	Bembicinae	Bembecini	Bembicina	*Bembix rostrata*
120707_I249_FCD111GACXX_L3_INSswpTBGRAAPEI-34.tsa.fas	Crabronidae	Bembicinae	Nyssonini	Nyssonina	*Nysson niger*
120707_I249_FCD111GACXX_L4_INSswpTBJRAAPEI-37.tsa.fas	Crabronidae	Crabroninae	Crabronini	Crabronina	*Crabro peltarius*
120707_I249_FCD111GACXX_L4_INSswpTBPRAAPEI-46.tsa.fas	Crabronidae	Crabroninae	Crabronini	Crabronina	*Crossocerus quadrimaculatus*
121221_I260_FCC1GFFACXX_L3_INSlupTBBRAAPEI-15.tsa.fas	Crabronidae	Crabroninae	Crabronini	Crabronina	*Lestica clypeata*
130728_I263_FCD23HKACXX_L8_INSofmTBTRAAPEI-94.tsa.fas	Crabronidae	Crabroninae	Larrini	Larrina	*Liris sp*
120707_I249_FCD111GACXX_L1_INSswpTAKRAAPEI-21.tsa.fas	Crabronidae	Crabroninae	Larrini	Gastrosericina	*Tachysphex fulvitarsis*
130928_I232_FCC2UV4ACXX_L1_RINSinlTDERAAPEI-83.tsa.fas	Crabronidae	Crabroninae	Miscophini		*Nitela sp*
120215_I277_FCD0KP1ACXX_L8_INSjdsTBIRAAPEI-75.tsa.fas	Crabronidae	Crabroninae	Oxybelini		*Oxybelus bipunctatus*
130728_I263_FCD23HKACXX_L2_INSofmTAURAAPEI-93.tsa.fas	Crabronidae	Crabroninae	Palarini		*Palarus histrio*
130206_I238_FCC1LVUACXX_L3_INSqiqTCLRAAPEI-44.tsa.fas	Crabronidae	Crabroninae	Trypoxylini		*Pison atrum*
120429_I266_FCC0HG0ACXX_L8_INSytvTAWRAAPEI-88.tsa.fas	Crabronidae	Crabroninae	Trypoxylini		*Trypoxylon figulus*
120215_I277_FCD0KP1ACXX_L8_INSjdsTAYRAAPEI-43.tsa.fas	Crabronidae	Dinetinae			*Dinetus pictus*
121221_I260_FCC1GFFACXX_L3_INSlupTBDRAAPEI-17.tsa.fas	Crabronidae	Mellininae			*Mellinus arvensis*
120215_I277_FCD0KP1ACXX_L7_INSjdsTBMRAAPEI-88.tsa.fas	Crabronidae	Pemphredoninae	Pemphredonini	Pemphredonina	*Diodontus minutus*
121221_I260_FCC1GFFACXX_L3_INSlupTBHRAAPEI-21.tsa.fas	Crabronidae	Pemphredoninae	Pemphredonini	Pemphredonina	*Passaloecus eremita*
120521_I249_FCC0U4RACXX_L7_INSytvTBBRAAPEI-95.tsa.fas	Crabronidae	Pemphredoninae	Pemphredonini	Pemphredonina	*Pemphredon lugens*
121221_I260_FCC1GFFACXX_L3_INSlupTBJRAAPEI-30.tsa.fas	Crabronidae	Pemphredoninae	Pemphredonini	Spilomenina	*Spilomena beata*
120707_I249_FCD111GACXX_L4_INSswpTATRAAPEI-13.tsa.fas	Crabronidae	Pemphredoninae	Psenini		*Psenulus fuscipennis*
120725_I247_FCC0WHRACXX_L4_INSswpTBTRABPEI-62.tsa.fas	Crabronidae	Philanthinae	Philanthini	Philanthina	*Philanthus triangulum*
130728_I263_FCD23HKACXX_L8_INSofmTBQRAAPEI-84.tsa.fas	Crabronidae	Philanthinae	Pseudoscoliini		*Pseudoscolia sinaitica*
130206_I238_FCC1LVUACXX_L4_INSqiqTDARAAPEI-109.tsa.fas	Halictidae				*Dufourea dentiventris*
120521_I249_FCC0U4RACXX_L7_INSytvTBIRAAPEI-15.tsa.fas	Halictidae				*Halictus quadricinctus*
120707_I249_FCD111GACXX_L1_INSswpTAORAAPEI-33.tsa.fas	Halictidae				*Lasioglossum xanthopus*
120521_I249_FCC0U4RACXX_L7_INSytvTBJRAAPEI-41.tsa.fas	Halictidae				*Nomia diversipes*
130314_I269_FCC1KFEACXX_L7_INSobdTCZRAAPEI-56.tsa.fas	Halictidae				*Nomioides sp.*
110817_I809_FCD05CDACXX_L3_INSbusTBPRAAPEI-81.tsa.fas	Halictidae				*Sphecodes albilabris*
120707_I249_FCD111GACXX_L3_INSswpTAXRAAPEI-17.tsa.fas	Halictidae				*Systropha curvicornis*
120707_I249_FCD111GACXX_L4_INSswpTAVRAAPEI-15.tsa.fas	Megachilidae				*Anthidium manicatum*
120707_I249_FCD111GACXX_L2_INSswpTAHRAAPEI-18.tsa.fas	Megachilidae				*Chelostoma florisomne*
120707_I249_FCD111GACXX_L4_INSswpTBLRAAPEI-41.tsa.fas	Megachilidae				*Coelioxys conoidea*
120707_I249_FCD111GACXX_L1_INSswpTADRAAPEI-14.tsa.fas	Megachilidae				*Dioxys cincta*
120521_I249_FCC0U4RACXX_L7_INSytvTBLRAAPEI-44.tsa.fas	Megachilidae				*Heriades truncorum*
130112_I269_FCC1M19ACXX_L8_INSlupTAWRAAPEI-9.tsa.fas	Megachilidae				*Lithurgus chrysurus*
120429_I266_FCC0HG0ACXX_L7_INSytvTAORAAPEI-39.tsa.fas	Megachilidae				*Megachile willughbiella*
110817_I809_FCD05CDACXX_L3_INSbusTBFRABPEI-126.tsa.fas	Megachilidae				*Osmia cornuta*
120521_I249_FCC0U4RACXX_L7_INSytvTBMRAAPEI-45.tsa.fas	Megachilidae				*Stelis punctulatissima*
121221_I260_FCC1GFFACXX_L3_INSlupTAYRAAPEI-12.tsa.fas	Melittidae				*Dasypoda hirtipes*
120521_I249_FCC0U4RACXX_L7_INSytvTBORAAPEI-47.tsa.fas	Melittidae				*Macropis fulvipes*
121221_I260_FCC1GFFACXX_L8_INSlupTBFRAAPEI-19.tsa.fas	Melittidae				*Melitta haemorrhoidalis*
121221_I260_FCC1GFFACXX_L3_INSlupTBLRAAPEI-33.tsa.fas	Sphecidae s.str.	Ammophilinae			*Ammophila sabulosa*
120707_I249_FCD111GACXX_L4_INSswpTBRRAAPEI-56.tsa.fas	Sphecidae s.str.	Ammophilinae			*Podalonia hirsuta*
130728_I263_FCD23HKACXX_L3_INSofmTBMRAAPEI-72.tsa.fas	Sphecidae s.str.	Chloriontinae			*Chlorion hirtum*
120521_I249_FCC0U4RACXX_L7_INSytvTBQRAAPEI-57.tsa.fas	Sphecidae s.str.	Sceliphrinae	Sceliphrini		*Chalybion californicum*
120707_I249_FCD111GACXX_L3_INSswpTAZRAAPEI-19.tsa.fas	Sphecidae s.str.	Sceliphrinae	Sceliphrini		*Sceliphron curvatum*
120521_I249_FCC0U4RACXX_L7_INSytvTBSRAAPEI-74.tsa.fas	Sphecidae s.str.	Sphecinae	Prionychini		*Prionyx kirbii*
120707_I249_FCD111GACXX_L3_INSswpTBDRAAPEI-30.tsa.fas	Sphecidae s.str.	Sphecinae	Sphecini		*Isodontia mexicana*
120429_I266_FCC0HG0ACXX_L7_INSytvTAIRAAPEI-18.tsa.fas	Sphecidae s.str.	Sphecinae	Sphecini		*Sphex funerarius*
**Outgroups**					
120429_I266_FCC0HG0ACXX_L7_INSytvTABRAAPEI-11.tsa.fas	Mutillidae				*Smicromyrme rufipes*
120707_I249_FCD111GACXX_L1_INSswpTABRAAPEI-12.tsa.fas	Pompilidae				*Pompilus cinereus*
120521_I249_FCC0U4RACXX_L8_INSytvTBVRAAPEI-84.tsa.fas	Sapygidae				*Sapyga quinquepunctata*
120521_I249_FCC0U4RACXX_L8_INSytvTBXRAAPEI-21.tsa.fas	Scoliidae				*Colpa sexmaculata*
120429_I266_FCC0HG0ACXX_L7_INSytvTADRAAPEI-13.tsa.fas	Scoliidae				*Scolia hirta*
120429_I266_FCC0HG0ACXX_L7_INSytvTAMRAAPEI-36.tsa.fas	Tiphiidae				*Tiphia femorata*

Additional table S7: Official gene sets used to assign assembled contigs from enriched DNA libraries and transcriptomes to 3,260 single-copy protein-coding genes.

Species	URL	Files	Date of Download
Acromyrmex echinatior	http://hymenopteragenome.org/ant_genomes	aech_OGSv3.8_pep.fa<br>aech_OGSv3.8_transcript.fa	August 10, 2014
Apis mellifera	http://hymenopteragenome.org/beebase/	amel_OGSv3.2_pep.fa<br>amel_OGSv3.2_cds.fa	August 7, 2014
Camponotus floridanus	http://hymenopteragenome.org/ant_genomes/	cflo_OGSv3.3_pep.fa.gz<br>cflo_OGSv3.3_transcript.fa.gz	August 10, 2014
Harpegnathos saltator	http://hymenopteragenome.org/ant_genomes/	hsal_OGSv3.3_pep.fa<br>hsal_OGSv3.3_transcript.fa	August 10, 2014
Tribolium castaneum	http://beetlebase.org	T_castaneum_3_CDS.fa<br>T_castaneum_3_peptide.fa	September 24, 2012
Nasonia vitripennis	http://arthropods.eugenes.org/EvidentialGene/nasonia/genes/	nvit2_evigenes_pub11u.good.aa.gz<br>nvit2_evigenes_pub11u.good.cds.gz	August 7, 2014

Additional table S8: Information on required taxa in the phylogenetic analysis. Each data block was demanded to contain sequence information of at least one representative species from each of the following groups. Otherwise a data block was removed due to poor taxonomic coverage. Each group in frame line - Group 7 additionally subdivided by several frame lines.

	Taxon
**Group 1**	*Acromyrmex echinatior*
	Camponotus floridanus
	Harpegnathos saltator
	Smicromyrme rufipes
	Pompilus cinereus
	Sapyga quinquepunctata
	Colpa sexmaculat
	Scolia hirta
	Tiphia femorata
**Group 2**	*Ammobates syriacus*
	Andrena vaga
	Anthidium manicatum
	Anthophora plumipes
	Apis mellifera II
	Apis mellifera
	Bombus rupestris
	Camptopoeum sacrum
	Ceratina chalybea
	Chelostoma florisomne
	Coelioxys conoidea
	Colletes cunicularius
	Dasypoda hirtipes
	Dioxys cincta
	Dufourea dentiventris
	Epeolus variegatus
	Eucera nigrescens
	Eucera plumigera
	Eucera syriaca
	Euglossa dilemma
	Halictus quadricinctus
	Heriades truncorum
	Hylaeus variegatus
	Lasioglossum xanthopus
	Lithurgus chrysurus
	Macropis fulvipes
	Megachile willughbiella
	Melitta haemorrhoidalis
	Nomada lathburiana
	Nomia diversipes
	Nomioides sp.
	Osmia cornuta
	Panurgus dentipes
	Sphecodes albilabris
	Stelis punctulatissima
	Stenotritidae
	Systropha curvicornis
	Tetragonula carbonaria
	Tetralonia malvae
	Tetraloniella nigriceps
	Tetraloniella sp.
	Thyreus orbatus
	Xylocopa violacea
**Group 3**	*Ammoplanellus* sp.
	Ammoplanus sp.
**Group 4**	*Diodontus minutus*
	Passaloecus eremita
	Pemphredon lugens
	Polemistus sp.
	Spilomena beata
	Carinostigmus sp.
	Stigmus sp.
**Group 5**	*Mimesa lutaria*
	Psenulus dilectus
	Psenulus fuscipennis
	Psenulus sp.
	Odontosphex paradoxus
**Group 6**	*Clypeadon sculleni*
	Clypeadon taurulus
	Cerceris acanthophila
	Cerceris arenaria
	Eucerceris arenaria
	Philanthus gibbosus
	Philanthus triangulum
	Trachypus sp.
	Pseudoscolia sinaitica
	Pseudoscolia sp.
**Group 7**	*Ampulex compressa*
	Ampulex fasciata
	Dolichurus corniculus
	Dolichurus sp.
	Astata minor
	Astata sp. I
	Astata sp. II
	Dryudella pinguis
	Dryudella sp.
	Dynatus burmeisteri
	Penepodium fumipenne
	Alysson spinosus
	Didineis sp.
	Acanthostethus sp.
	Brachystegus sp.
	Nysson niger
	Synnevrus sp.
	Heterogyna nocticula
	Heliocausus sp.
	Tiguipa sp.
	Arigorytes sp.
	Gorytes albidulus
	Gorytes laticinctus
	Harpactus elegans
	Hoplisoides sp.
	Liogorytes sp.
	Psammaecius versicolor
	Pseudoplisus willcoxi
	Ammatomus sp. I
	Ammatomus sp. II
	Tanyoprymnus moneduloides
	Tanyoprymnus sp.
	Bembecinus sp.
	Stizoides foxi
	Stizoides tridentatus
	Stizus aff marthae
	Stizus continuus
	Stizus vespoides
	Chlorion hirtum
	Chlorion sp.
	Chalybion californicum
	Chalybion sp. I
	Chalybion sp. II
	Sceliphron curvatum
	Ammophila sabulosa
	Ammophila sp. II
	Eremnophila melanaria
	Podalonia hirsuta
	Podalonia sp.
	Palmodes sp.
	Prionyx kirbii
	Prionyx subatratus
	Isodontia mexicana
	Sphex funerarius I
	Sphex funerarius II
	Stangeella cyaniventris
	Pison atrum
	Pison sp.
	Trypoxylon figulus
	Trypoxylon lactitarse
	Lyroda sp.
	Nitela sp.
	Paranysson sp.
	Sphodrotes sp.
	Holotachysphex sp.
	Larropsis sp.
	Prosopigastra nearctica
	Tachysphex fulvitarsis
	Tachysphex sp.
	Tachytes amazonus
	Tachytes sp.
	Larra sp.
	Liris sp.
	Liris sp.
	Belomicrus sp.
	Oxybelus bipunctatus
	Anacrabro sp.
	Entomognathus sp.
	Crabro peltarius
	Crabro scutellatus
	Crossocerus quadrimaculatus
	Dasyproctus sp.
	Ectemnius sonorensis
	Foxita barassp. patei I
	Foxita bara ssp. *patei* II
	Lestica alata
	Lestica clypeata
	Lestica sp.
	Lindenius panzeri
	Moniaecera sp.
	Neodasyproctus sp. I
	Neodasyproctus sp. II
	Quexua sp.
	Rhopalum clavipes
	Sphecius convallis
	Sphecius spectabilis
	Bembix rostrata
	Bicyrtes ventralis
	Microbembex sp.
	Rubrica nasuta
	Selman notatus
	Stictia heros
	Trichostictia sp.
	Microstictia hurdi
	Steniolia duplicata
	Stictiella sp.
	Palarus histrio
	Dinetus pictus
	Mellinus arvensis

Additional table S9: Fossils used for time divergence time estimations. Besides the calibration time we also give the prior settings. Mode of preservation: CF - compression fossil; AM - amber. Information about type locality obtained from the database PaleoDB. Ma = million years. Time scale = 100 Ma for lower bound setting.

Fossil	PaleoDB taxon No.	placing node	fossil calibration node shown in Additional figure S1	age range [Ma]	lower bound (tL)	left tail probability [p]	Basis of Age	Lithology	Environment	Location	Phylogenetic / taxonomic justification	Reference
Pison antiquum	219890	Trypoxylini: *Pison* / *Trypoxylon*	**4**	20.43 to 13.65	0.1365	0,025	Stratigraphy based	Dominican amber	terrestrial	OSU H-10-15	*Pison antiquum* has not been included in any phylogenetic analysis, nor has any other fossil species in this genus. However, in the original description, Antropov and Pulawski (1996) clearly mentioned diagnostic characters and character combinations, which potentially qualify as apomorphies of *Pison* in the context of the well-founded placement of the genus in the Trypoxylini and the Larrinae.	[1]
Lindenius paleomystax	224829	Crabronina: *Lindenius* / *Crabro* / *Lestica* / *Crossocerus*	**3**	20.43 to 13.65	0.1365	0,025	Stratigraphy based	Dominican amber	terrestrial	AMNH DR-14-1091	*Lindenius paleomystax* has not been included in any published phylogenetic analysis, nor has any other fossil species in this genus. However, in the original description, Bennett and Engel (2006) clearly mentioned diagnostic characters and character combinations, which potentially qualify as apomorphies of *Lindenius* in the context of the well-founded placement of the genus in the Crabronini and the Crabroninae. Additionally, the species has been compared with the result of a cladistic analysis of the Crabronini and its generic placement has been confirmed (unpublished PhD. thesis D. Bennett, 2010).	[2]
Passaloecus microceras	255469	Pemphredonina: *Polemistus* / *Passaloecus*	**7**	58 to 34 (age of Baltic amber problematic) [10,11]	0.34	0,025	Stratigraphy based and K-Ar dates	Baltic amber	terrestrial	Kaliningrad	*Passaloecus microceras*has not been included in any phylogenetic analysis, nor has any other fossil species in this genus. However, in the original description, Sorg (1986) clearly mentioned diagnostic characters and character combinations, which potentially qualify as apomorphies of *Passaloecus* in the context of the well-founded placement of the genus in the Pemphredonii and the Pemphredoninae. These characters haven been used in a cladistic analysis of the Pemphredoninae by Antropov (2011) and their phylogenetic significance has beenconfirmed.	[3]
Psammaecius sepultus	320781	Speciina / Gorytina	**5**	37.2 to 33.9	0.339	0,025	Stratigraphy based	Chadronian lacustrine	terrestrial	Florissant MCZ 2019	The original description of *Psammaecius sepultus* (in *Hoplisus*) left many open questions. The specimen was redescribed by Pulawski and Rasnitsyn (1980) in great detail. They also presented a detailed discussion of the placement in the family, tribe and genus. Although fossil bembicines have been neglected in all cladistic analyses of the Bembecinae, the significance of the characters to support placement of the fossil in the Gorytini can be confirmed.	[4]
Hoplisidea kohliana	179708	Chloriontinae / Sceliphrini / Podiini	**2**	37.2 to 33.9	0.339	0,025	Stratigraphy based	Chadronian lacustrine	terrestrial	Florissant MCZ 2018	Menke and Rasnitsyn (1987) restudied the holotype of *Hoplisidea kohliana*. They discussed potential affinities within apoid wasps, although without rigorous cladistic methods. They concluded that a placement within Sceliphrinae is most likely based on significant similarities in the wing venation.	[4]
Psolimena electra	227250	Spilomenina / Stigmina / Pemphredonina	**6**	94.3 to 89.3	0.893	0,025	Stratigraphy based	White Oaks Pit (Old Crossman's Clay Pits)	terrestrial	AMNH NJ-268	*Psilomena electra* has been included in phylogenetic analysis of the Pemphredoninae by Antropov (2011) and its placement in the Spilomenini has been confirmed.	[5]
Cretampulex gracilis	255374	Apoidea	**1**	98.79 ± 0.62	0,982	0,025	206Pb/238U [12]	Burmese amber	terrestrial	NHM In. 19123(5)	The position of *Cretampulex gracilis* has been discussed in detail by the original author (Antropov 2000). Later, the species was included in an overview discussion on the fossil Ampulicidae, and the relavant characters were used in a cladistic analysis of the Ampulicidae (Ohl and Spahn 2010).	[6]
Melittosphex burmensis	213427	Ammoplanina / Anthophila	**8**	98.79 ± 0.62	0.982	0,025	206Pb/238U [12]	Burmese amber	terrestrial	Poinar collection	*Melittosphex burmensis* is usually seen as the oldest known representative of the bees (Anthophila). This has been emphasized by the original authors, who particularly emphasized the presence of plumose hairs as one of the unique apomorphies of the bees within Apoidea. The species has also been used in an intuitive calibration of a cladogram of the relationships within Apoidea by Ohl and Engel (2007).	[7]
Palaeomacropis eocenicus	227437	Bombini / Meliponini	**9**	53 to 50	0.5	0,025	Sparnacian, level MP7 of the mammalian fauna of Dormaal [13,14]	Oise amber	terrestrial	MNHN PA 3190	In the description of this species, the authors list a number of characters that allow placement of the fossil in Macropidinae (some of them are listed here in the "apomorphies" column). They, however, also list some characters that this species shares with other melittid genera. For details, refer to Michez et al. 2007	[8]
Paleoepeolus micheneri	355783	Ammobatini / Epeolini	**10**	61 to 60	0.6	0,025	Pollen analysis and radiometric K/Ar analysis [15]	Thanetian crater lake diatomite in the Menat Formation of France	terrestrial	Menat (MNHN coll)		[9]

References:

[1] - Antropov AV and Pulawski WJ (1996). Pison antiquum, a new species from Dominican amber (Hymenoptera: Sphecidae). Journal of Hymenoptera Research 5, 16–21.
[2] - Bennett DJ and Engel MS (2006). A new moustache wasp in Dominican amber, with an account of apoid wasp evolution emphasizing Crabroninae (Hymenoptera: Crabronidae). American Museum Novitates 3529, 1–10.
[3] - Sorg M (1986). Grabwespen der Gattung Passaloecus aus fossilen Harzen (Hymenoptera, Sphecoidea, Pemphredoninae). Paläontologische Zeitschrift 60, 277–284.
[4] - Rohwer SA, Brues CT, Cockerell TDA, Expeditions F, Cockerell WP, Wheeler WM (1906). Fossil parasitic and phytophagous Hymenoptera from Florissant, Colorado. American Museum of Natural History 22, 491–498.
[5] - Antropov AV (2000). A new digger wasp (Hymenoptera, Sphecidae, Pemphredoninae) from New Jersey amber. In: Studies on fossils in amber, with particular reference to the Cretaceous of New Jersey (D. Grimaldi, ed.) pp. 339–343. Back: Leiden.
[6] - Antropov AV (2000). Digger wasps (Hymenoptera, Sphecidae) in Burmese amber. Bulletin of the Natural History Museum, Geology Series 56, 59–77.
[7] - Poinar GO and Danforth BN (2006). A fossil bee from Early Cretaceous Burmese amber. Science 314, 614–614.
[8] - Michez D, Nel A, Menier JJ, Rasmont P (2007). The oldest fossil of a melittid bee (Hymenoptera: Apiformes) from the early Eocene of Oise (France). Zool. J. Linn. Soc. 250, 701–709.
[9] - Dehon M, Engel MS, Michez D (2017). Systematics, in Antiquity of cleptoparasitism among bees revealed by morphometric and phylogenetic analysis of a Paleocene fossil nomadine (Hymenoptera: Apidae). *Systematic Entomology* **42**:543-554.
[10] - Dlussky GM, Rasnitsyn AP (2009). Ants (Insecta: Vespida: Formicidae) in the Upper Eocene Amber of Central and Eastern Europe. Paleontol. J. 43, 1024–1042.
[11] - Standke G (2008). Bitterfelder Bernstein gleich Baltischer Bernstein? – Eine geologische Raum- Zeit- Betrachtung und genetische Schlußfolgerungen. Exkurs.f. und Veröfftl. DGG 236, 11–33.
[12] - Shi Guanghai *et al*. (2012). Age constraint on Burmese amber based on U–Pb dating of zircons. Cretaceous research, 37, 155-163.
[13] - Feugueur L (1963). L'Yprésien du Bassin de Paris. Essai de Monographie Stratigraphique (Paris: Imprimerie nationale).
[14] - Nel A, de Plöeg G, Dejax J, Dutheil D, de Franceschi D, Gheerbrant E, Godinot M, Hervet S, Menier JJ, Augé M, et al. (1999). Un gisement sparnacien exceptionnel à plantes, arthropodes et vertébrés (Éocène basal, MP7): Le Quesnoy (Oise, France). C. R. Acad. Sci., Ser. IIa: Sci. Terre Planets 329, 65–72.
[15] - Wappler T, Currano ED, Wilf P, Rust J, Labandeira CC (2009). No post-Cretaceous ecosystem depression in European forests? Rich insect-feeding damage on diverse middle Palaeocene plants, Menat, France. *Proceedings of the Royal Society of London B: Biological Sciences*, *276*(1677), 4271-4277.

Additional table S10. Four-cluster Likelihood (FcLM) results on amino acid and nucleotide level when testing the phylogenetic placement of Ammoplanina and Mellininae. Proportions of quartets that map into areas respective areas in the 2D-simplex graph. T1: unambiguous support for G1,G2 – G3,G4. T2: unambiguous support for G1,G3 – G2,G4. T3: unambiguous support for G1,G4 – G2,G3. Quartets falling into the areas T1, T2 and T3 are informative. Areas T12, T23 and T13 are partly informative, and area T* is not informative.

Hypothesis 1: Does FcLM support a sister group relationship between Ammoplanina and Anthophila (bees)?
possible unambiguous topologies:
T1: Anthophila,Ammoplanina | Psenini + Odontosphecini,rem_apoid_wasps_outgroups
T2: Anthophila,Psenini + Odontosphecini | Ammoplanina,rem_apoid_wasps_outgroups
T3: Anthophila,rem_apoid_wasps_outgroups | Ammoplanina,Psenini + Odontosphecini

**Result on amino-acid level**	**T1: G1,G2|G3,G4 (area 1)**	**T2: G1,G3|G2,G4**	**T3: G1,G4|G2,G3**	**T1T2**	**T1T3**	**T2T3**	**T***
original	38,0%	15,0%	40,0%	2,0%	3,0%	1,0%	1,0%
permutation I	7,0%	5,0%	9,0%	8,0%	9,0%	10,0%	53,0%
permutation II	2,0%	1,0%	3,0%	4,0%	6,0%	5,0%	78,0%
permutation III	0,0%	0,0%	0,0%	0,0%	0,0%	0,0%	99,0%

**Result on nucleotide level**	**T1: G1,G2|G3,G4 (area 1)**	**T2: G1,G3|G2,G4**	**T3: G1,G4|G2,G3**	**T1T2**	**T1T3**	**T2T3**	**T***
original	54,0%	9,0%	34,0%	1,0%	2,0%	0,0%	0,0%
permutation I	20,0%	6,0%	14,0%	10,0%	9,0%	12,0%	28,0%
permutation II	4,0%	2,0%	2,0%	5,0%	5,0%	6,0%	76,0%
permutation III	2,0%	1,0%	1,0%	4,0%	5,0%	5,0%	82,0%

Hypothesis 2: Does FcLM support the position of Mellininae as sister to Sphecidae?
possible unambiguous topologies:
T1: Mellininae,Sphecidae | Crabroninae+Dinetinae, rem_apoid_wasps_bees_outgroups
T2: Mellininae,Crabroninae+Dinetinae | Sphecidae, rem_apoid_wasps_bees_outgroups
T3: Mellininae, rem_apoid_wasps_bees_outgroups | Sphecidae, Crabroninae+Dinetinae

**Result on amino-acid level**	**T1: G1,G2|G3,G4 (area 1)**	**T2: G1,G3|G2,G4**	**T3: G1,G4|G2,G3**	**T1T2**	**T1T3**	**T2T3**	**T***
original	21,0%	21,0%	52,0%	1,0%	2,0%	2,0%	1,0%
permutation I	8,0%	8,0%	7,0%	8,0%	9,0%	12,0%	49,0%
permutation II	1,0%	1,0%	2,0%	2,0%	2,0%	3,0%	89,0%
permutation III	0,0%	0,0%	0,0%	0,0%	0,0%	0,0%	99,0%

**Result on nucleotide level**	**T1: G1,G2|G3,G4 (area 1)**	**T2: G1,G3|G2,G4**	**T3: G1,G4|G2,G3**	**T1T2**	**T1T3**	**T2T3**	**T***
original	24,0%	20,0%	53,0%	0,0%	1,0%	1,0%	0,0%
permutation I	28,0%	8,0%	10,0%	7,0%	6,0%	24,0%	18,0%
permutation II	1,0%	3,0%	2,0%	4,0%	4,0%	8,0%	78,0%
permutation III	2,0%	1,0%	2,0%	4,0%	4,0%	6,0%	82,0%
