# Supplementary material for: Phylogenomic analysis of Apoidea sheds new light on the sister group of bees
Source: BMC Evol Biol. 2018 May 18;18:71. doi: 10.1186/s12862-018-1155-8 (PMC5960199; doi:10.1186/s12862-018-1155-8)
Supplement: Supplementary file 2 — Figure S1. Ultrametric and time-calibrated tree of Apoidea estimated using a relaxed molecular clock approach as implemented in MCMCtree. The estimates are based on the analysis of 284,607 nucleotide sites and applying a combination of protein domain – and codon-based partitioning scheme by modelling 1st, 2nd and 3rd codon positions separately. Figure S2. Phylogenetic relationships of Apoidea. The phylogenetic tree was inferred from analyzing 94,869 amino acid sites under the maximum likelihood (ML) optimality criterion. The data matrix was partitioned based on a protein domain-based partitioning scheme and analyzed with partition-specific substitution models. Node labels indicate bootstrap branch support values derived from 150 bootstrap replicates. Figure S2.1. Phylogenetic relationships of Apoidea. The phylogenetic tree was inferred with ExaBayes, by analyzing 94,869 amino acid sites. The data matrix was partitioned based on a protein domain-based partitioning scheme and analyzed with partition-specific substitution models automatically selected by ExaBayes. Posterior probability values were inferred from a total of 13,500 sampled trees. Figure S3. Phylogenetic relationships of Apoidea. The phylogenetic tree was inferred from analyzing 284.607 nucleotide sites under the maximum likelihood (ML) optimality criterion. The data matrix was partitioned based on applying a combination of protein domain – and codon-based partitioning scheme by modelling 1st and 2nd codon positions separately and excluding the 3rd codon positions. Each partition was analyzed with the partition-specific model parameters under the nucleotide substitution model GTR + G. Node labels indicate bootstrap branch support values derived from 150 bootstrap replicates. Figure S3.1. Phylogenetic relationships of Apoidea. The phylogenetic tree was inferred with ExaBayes, by analyzing 284.607 nucleotide sites. The data matrix was partitioned based on applying a combination of protein domain – and codon- [file 12862_2018_1155_MOESM2_ESM.pdf]

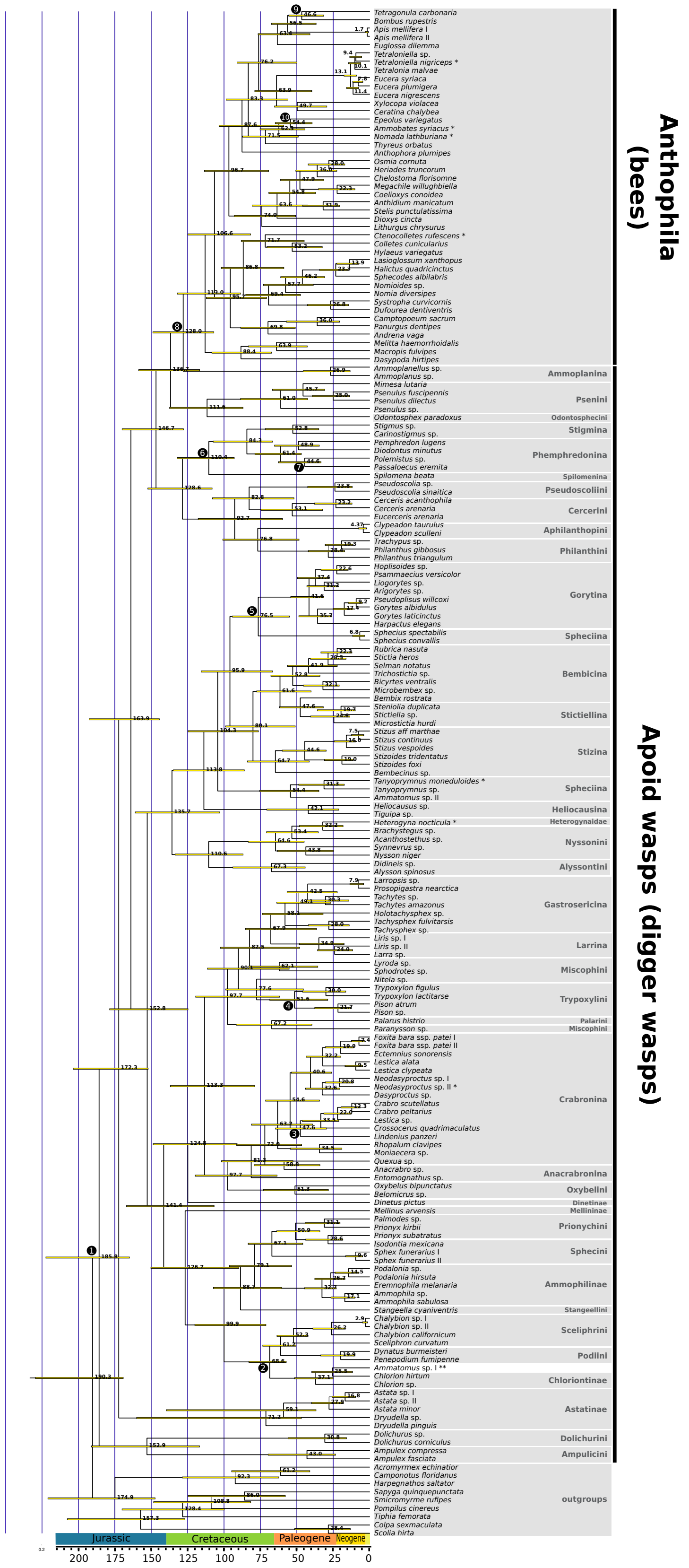

Additional figure S1: Ultrametric and time-calibrated tree of Apoidea estimated using a relaxed molecular clock approach as implemented in MCMCtree. The node ages, the mean (numbers) and the 95 % confidence intervals (bars) are shown. The estimates are based on the analysis of 284,607 nucleotide sites and applying a combination of protein domain – and codon-based partitioning scheme by modeling 1st, 2nd and 3rd codon positions separately. We considered nine calibration points and specified them as lower (minimal age) soft bounds. Numbers on nodes indicate fossil calibration nodes (Supplementary table S9). Species marked by an asterisk (*) indicate rogue taxa. Two asterisks (**) point to the misplaced species *Ammatomus sp*. I and (***) to the position of the Stenotritidae. Names according to the "Catalog of Sphecidae *senu lato*" by Pulawski (2016).

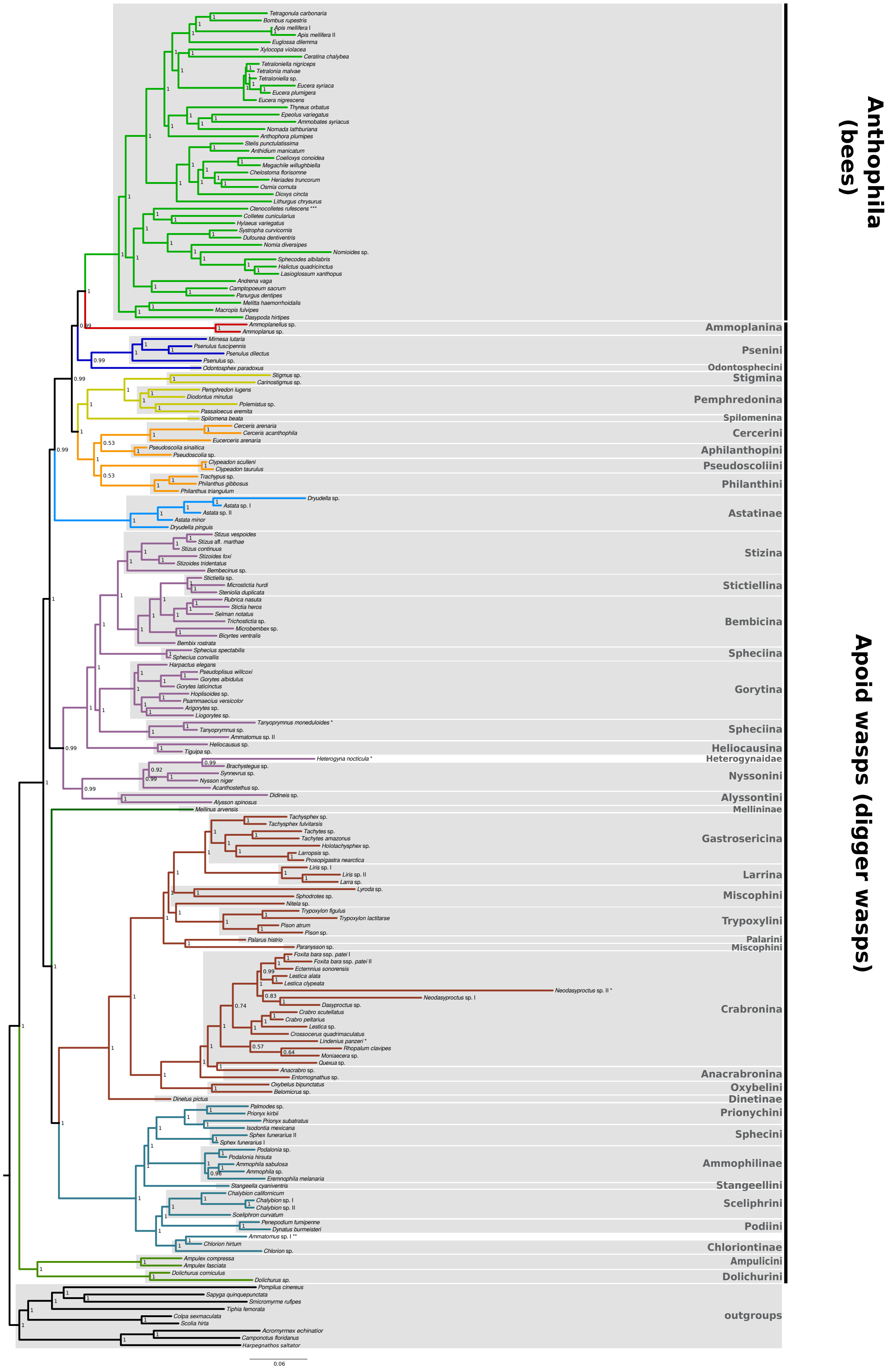

Additional figure S2.1: Phylogenetic relationships of Apoidea. The phylogenetic tree was inferred from analyzing 94,869 amino acid sites with ExaBayes (Aberer *et al.*, 2014). The data matrix was partitioned based on a protein domain-based partitioning scheme and analyzed with partition-specific substitution models automatically selected by ExaBayes. Posterior probability values were inferred from a total of 13,500 sampled trees. Species marked by an asterisk (*) indicate rogue taxa. Two asterisks (**) point to the misplaced species Ammatomus sp. I and (***) to the position of the Stenotritidae. Names according to W. J. Pulawski's "Catalog of Sphecidae sensu lato".

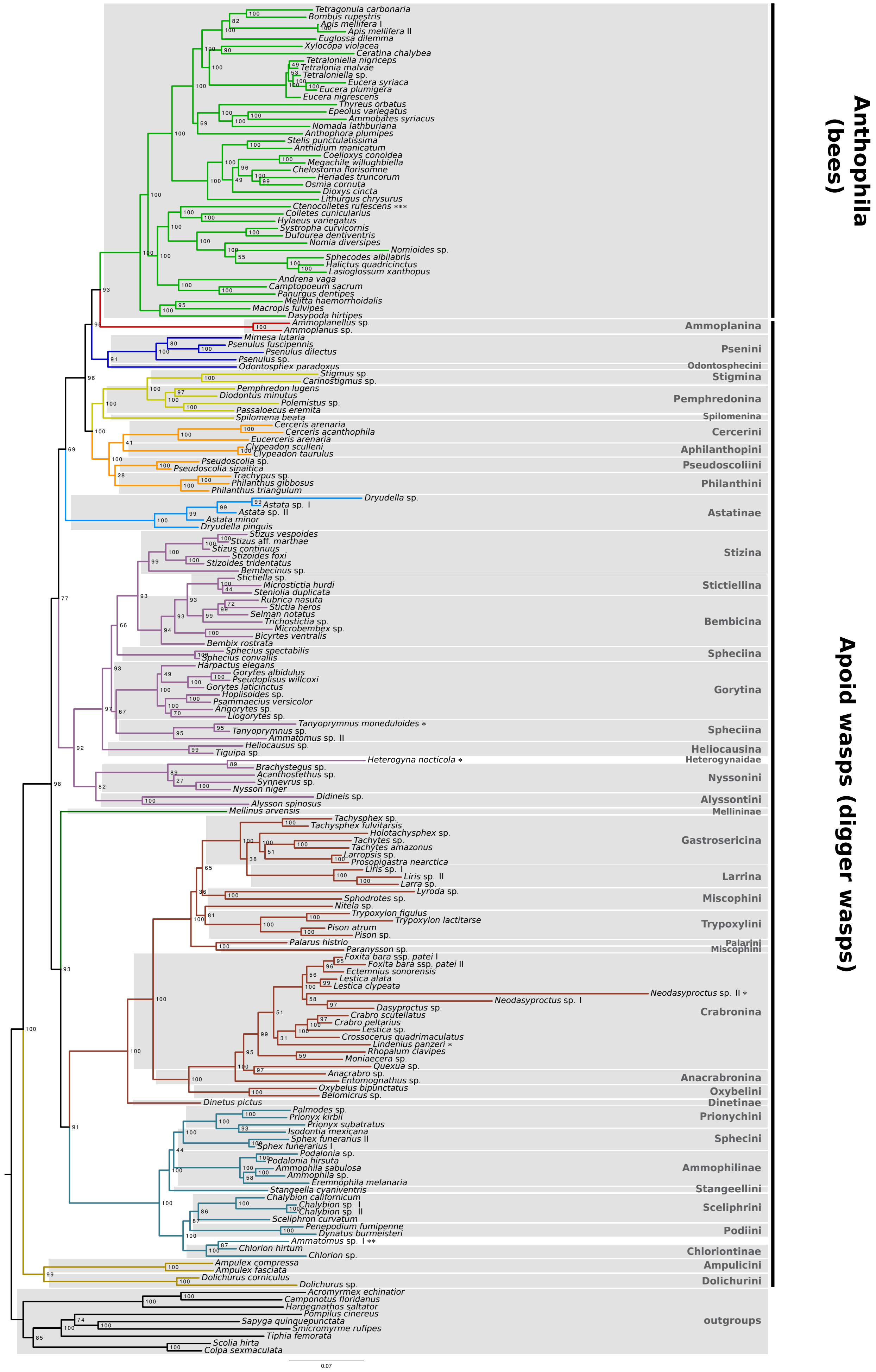

Additional figure S2: Phylogenetic relationships of Apoidea. The phylogenetic tree was inferred from analyzing 94,869 amino acid sites under the maximum likelihood (ML) optimality criterion. The data matrix was partitioned based on a protein domain-based partitioning scheme and analyzed with partition-specific substitution models. Node labels indicate bootstrap branch support values derived from 150 bootstrap replicates. Species marked by an asterisk (*) indicate rogue taxa. Two asterisks (**) point to the misplaced species *Ammatomus* sp. I and (***) to the position of the Stenotritidae. Names according to W. J. Pulawski's "Catalog of Sphecidae *sensu lato*".

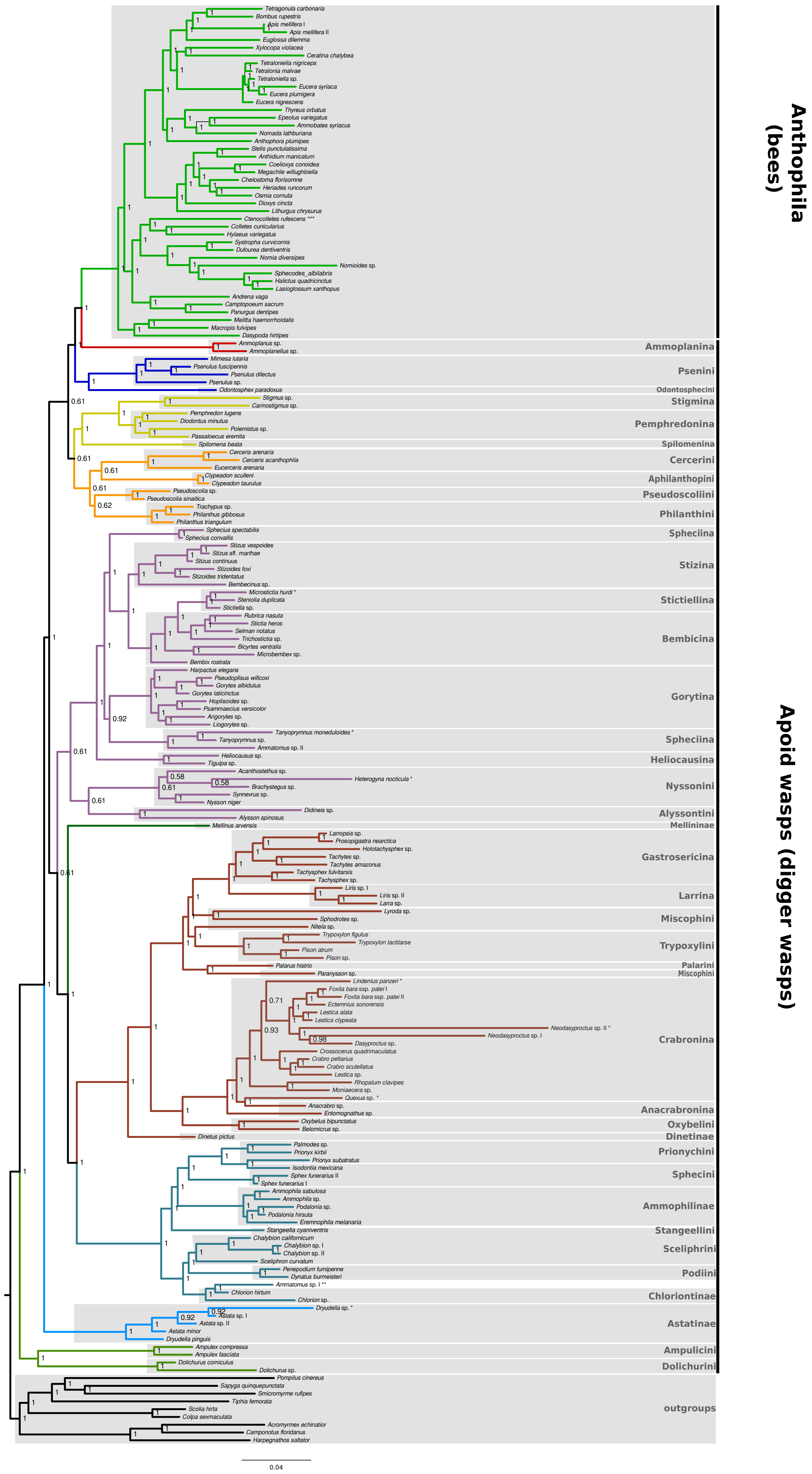

Additional figure S3.1: Phylogenetic relationships of Apoidea. The phylogenetic tree was inferred from analyzing 284.607 nucleotide sites with ExaBayes (Aberer *et al.*, 2014). The data matrix was partitioned based on applying a combination of protein domain – and codon-based partitioning scheme by modeling 1st and 2nd codon positions separately and excluding the 3rd codon positions. Each partition was analyzed with the partition-specific model parameters under the nucleotide substitution model GTR+G. Posterior probability values were inferred from a total of 13,500 sampled trees. Species marked by an asterisk (*) indicate rogue taxa. Two asterisks (**) point to the misplaced species *Ammatomus sp.* I and (***) to the position of the Stenotritidae. Branch colours correspond to level of support of monophyletic lineages. Names according to W. J. Pulawski's "Catalog of Sphecidae *sensu lato*".

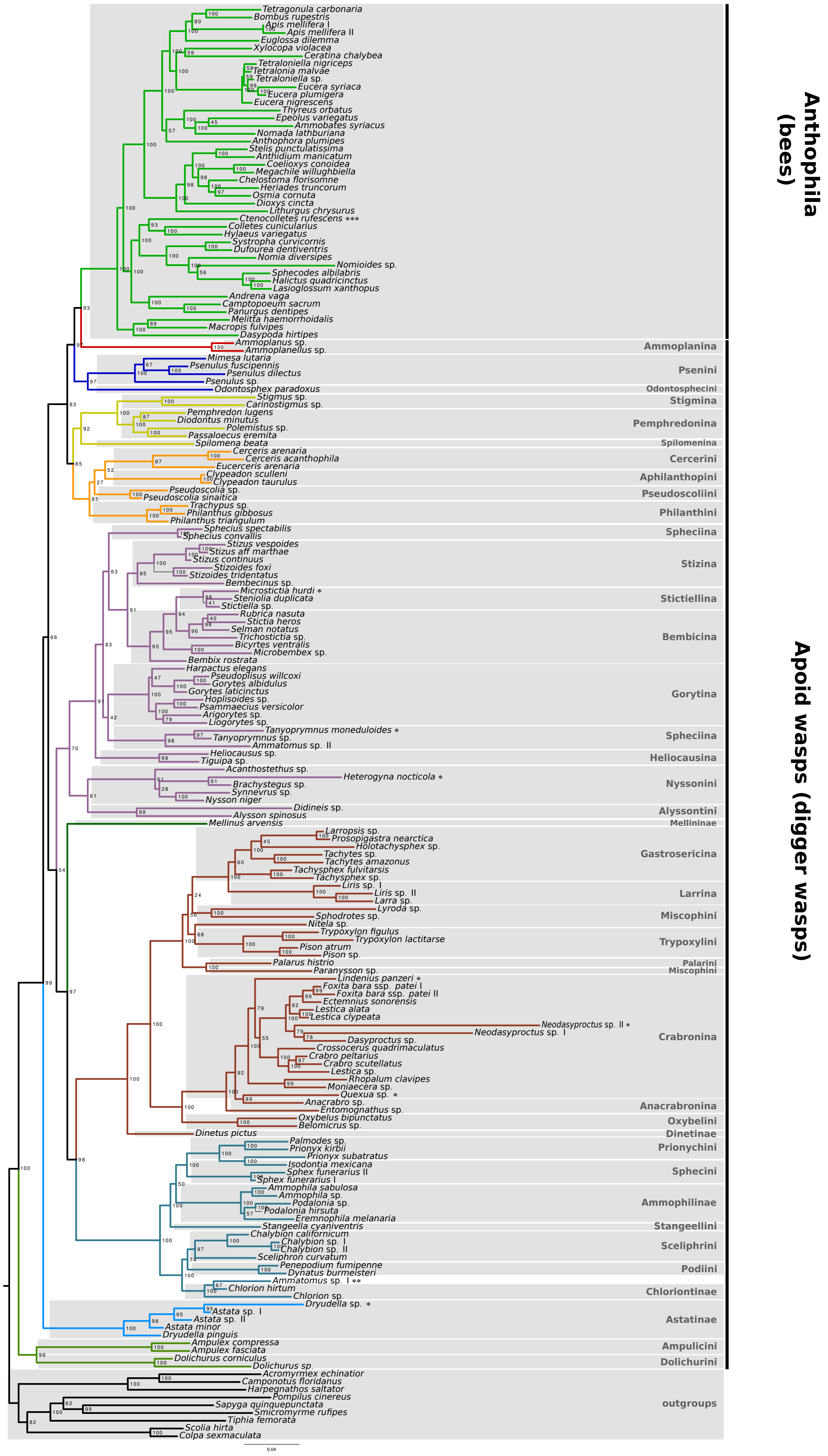

Additional figure S3: Phylogenetic relationships of Apoidea. The phylogenetic tree was inferred from analyzing 284.607 nucleotide sites under the maximum likelihood (ML) optimality criterion. The data matrix was partitioned based on applying a combination of protein domain – and codon-based partitioning scheme by modeling 1st and 2nd codon positions separately and excluding the 3rd codon positions. each partition was analyzed with the partition-specific model parameters under the nucleotide substitution model GTR +G. Node labels indicate bootstrap branch support values derived from 150 bootstrap replicates. Species marked by an asterisk (*) indicate rogue taxa. Two asterisks (**) point to the misplaced species *Ammatomus sp.* I and (***) to the position of the Stenotritidae. Branch colours correspond to level of support of monophyletic lineages. Names according to W. J. Pulawski's "Catalog of Sphecidae *sensu lato*".

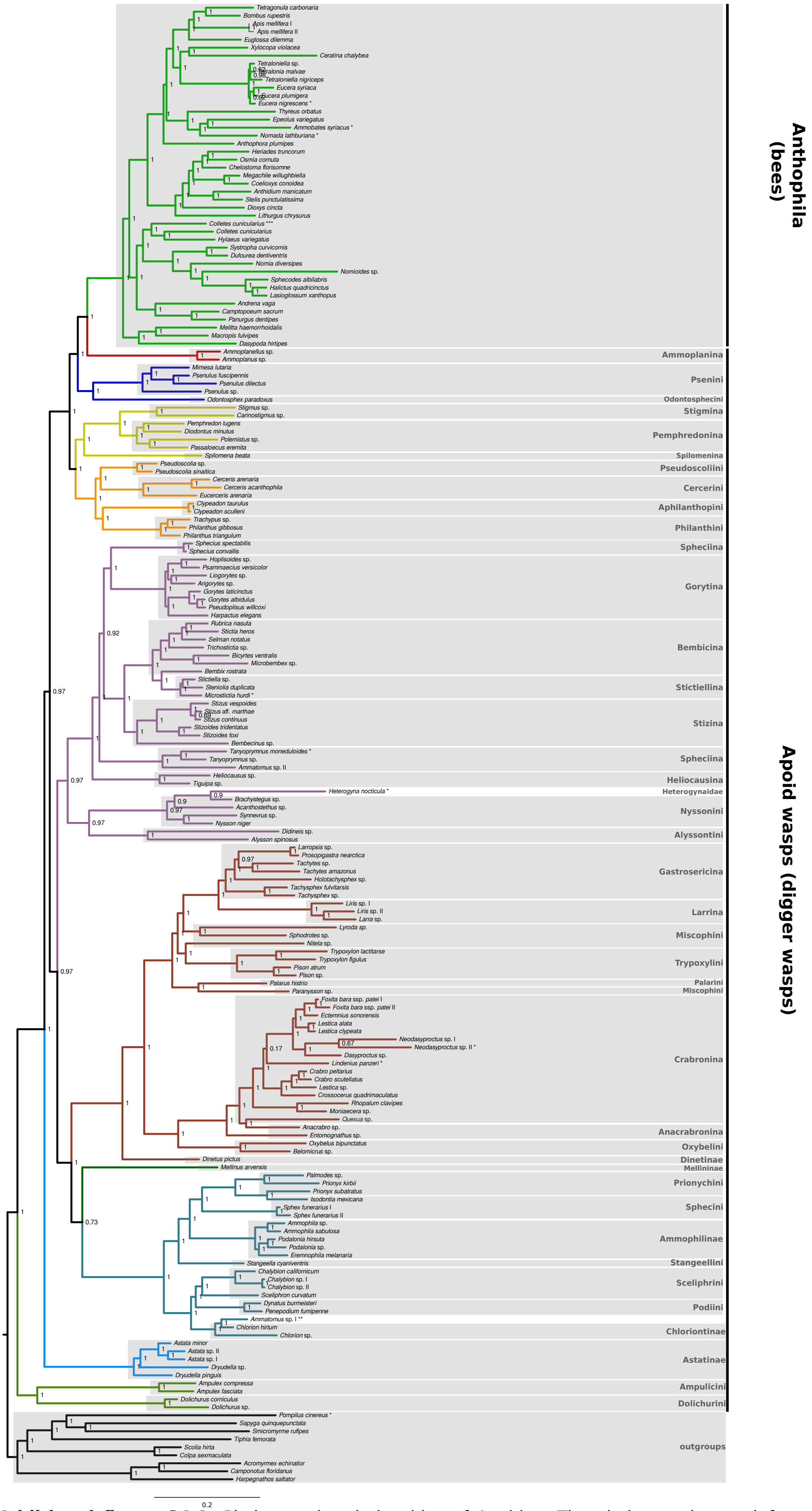

Additional figure S4.1: Phylogenetic relationships of Apoidea. The phylogenetic tree inferred from analyzing 284.607 nucleotide sites with ExaBayes (Aberer *et al*., 2014). The data matrix was partitioned based on applying a combination of protein domain – and codon-based partitioning scheme by modeling the 1st, 2nd and 3rd codon position separately. Each partition was analyzed with the partition-specific model parameters under the nucleotide substitution model GTR+G. Posterior probability values were inferred from a total of 13,500 sampled trees. Species marked by an asterisk (*) indicate rogue taxa. Two asterisks (**) point to the misplaced species *Ammatomus* sp. I and (***) to the position of the Stenotritidae. Branch colors correspond to level of support of monophyletic lineages. Names according to W. J. Pulawski's "Catalog of Sphecidae *sensu lato*".

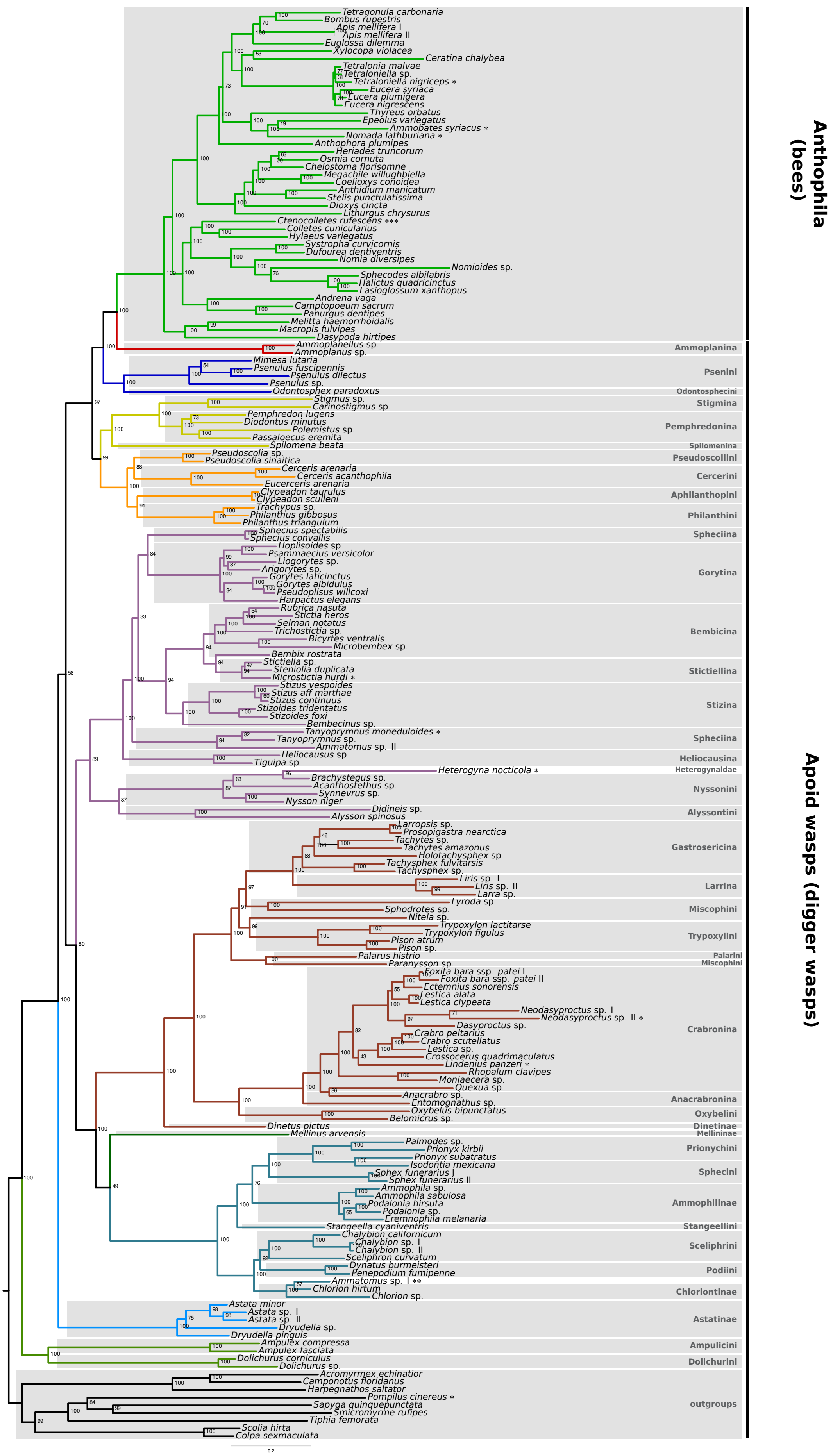

Additional figure S4: Phylogenetic relationships of Apoidea. The phylogenetic tree inferred from analyzing 284.607 nucleotide sites under the maximum likelihood (ML) optimality criterion. The data matrix was partitioned based on applying a combination of protein domain – and codon-based partitioning scheme by modeling the 1st, 2nd and 3rd codon position separately. Each partition was analyzed with the partition-specific model parameters under the nucleotide substitution model GTR+G. Node labels indicate bootstrap branch support values derived from 100 bootstrap replicates. Species marked by an asterisk (*) indicate rogue taxa. Two asterisks (**) point to the misplaced species *Ammatomus* sp. I and (***) to the position of the Stenotritidae. Branch colors correspond to level of support of monophyletic lineages. Names according to W. J. Pulawski's "Catalog of Sphecidae *sensu lato*".

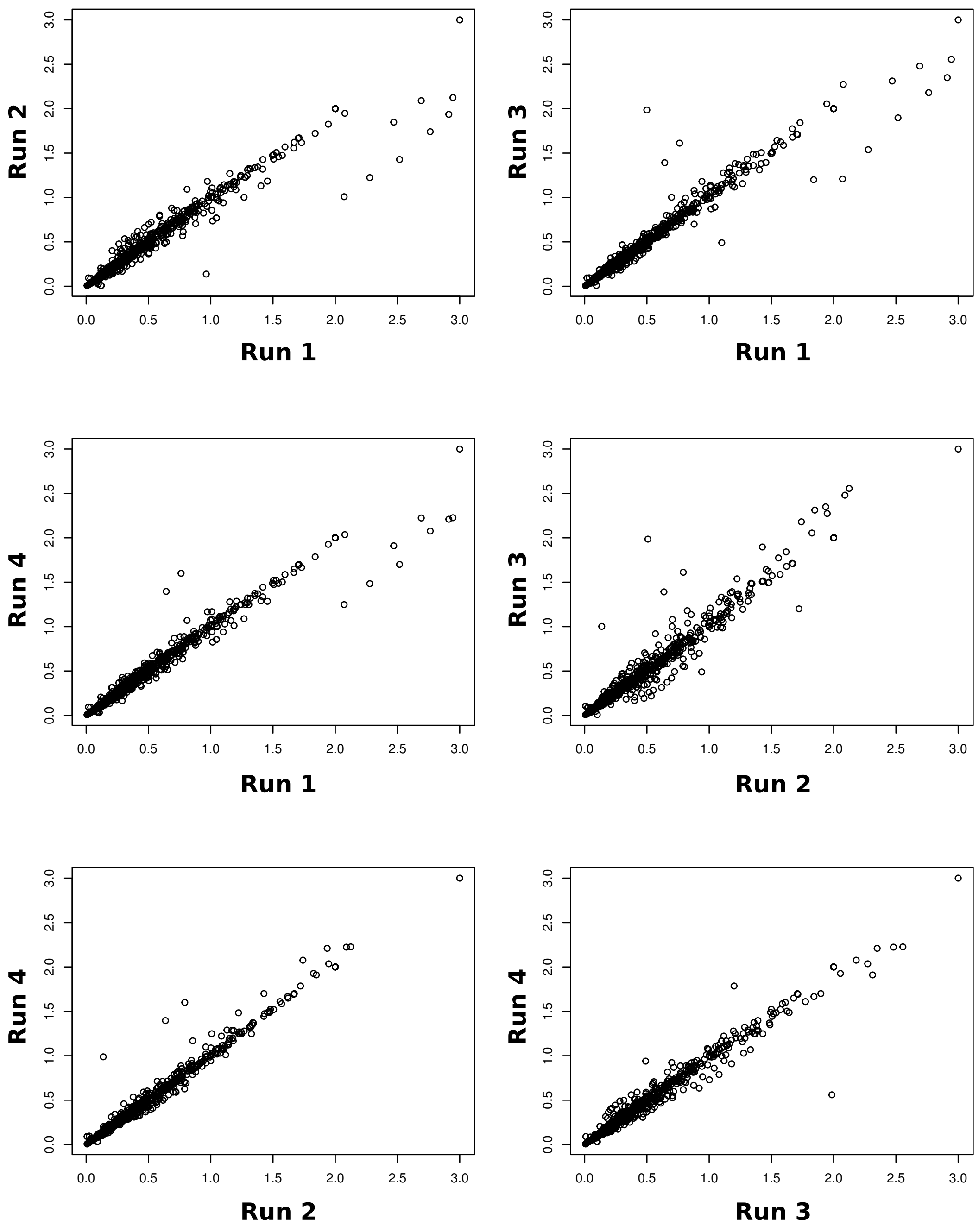

Additional figure S5: MCMCtree convergence comparison of four independent analyses. Pairwise comparison of the posterior means of divergence times obtained from the approximate likelihood calculation. Node ages and 95 % confidence intervals of four MCMCtree analysis replicates are plotted against each other.

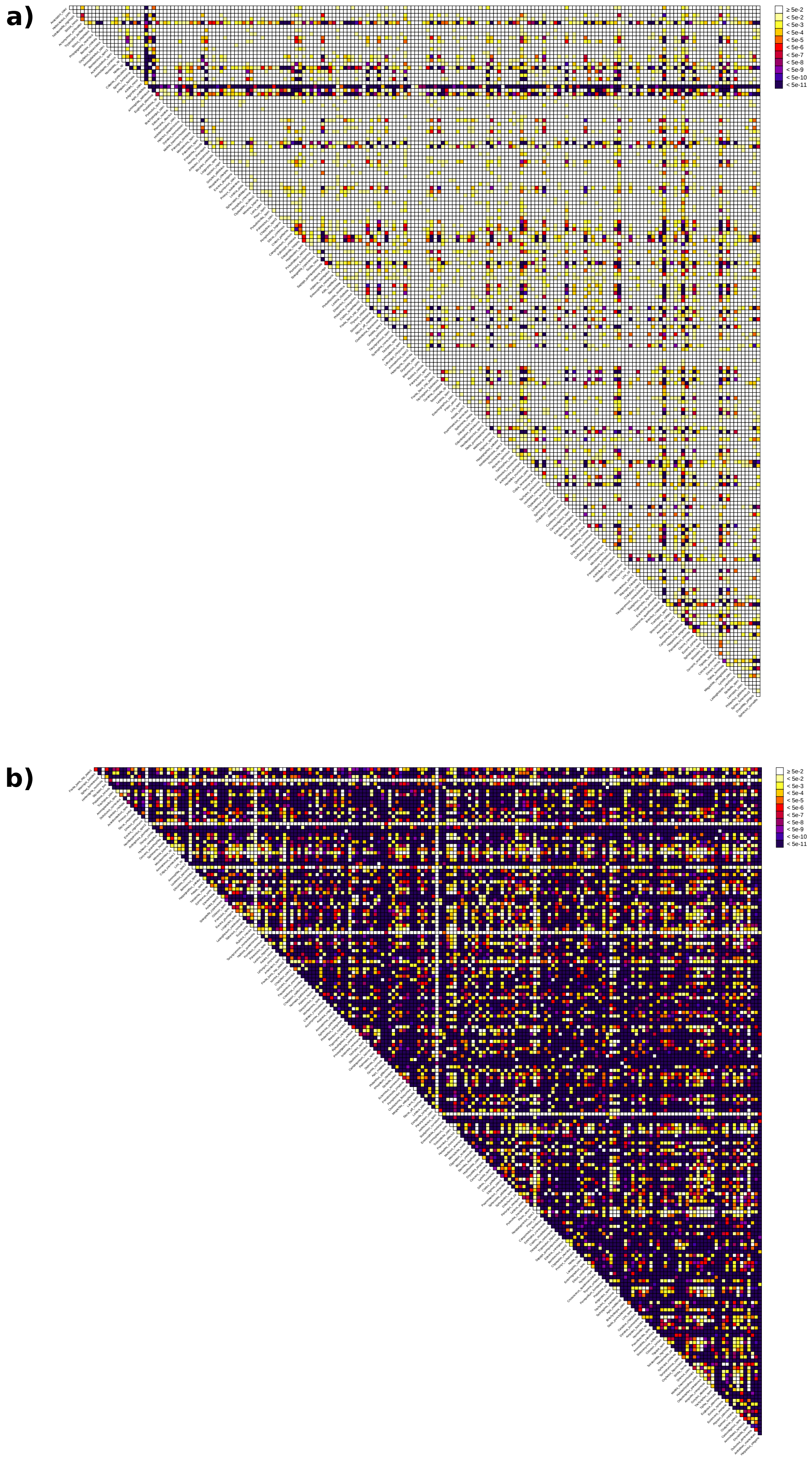

Additional figure S6: Bowker' matched-pairs test of symmetry. Heat maps showing the results from pairwise comparison of aligned **a**) amino acid dataset and **b**) nucleotide dataset with all three codon positions included (PF-NT-1,2,3). White cells specify *p*-values > 0.05, indicating that corresponding pairs of nucleotide sequences do not violate the assumption of global stationary, reversibility, and homogeneity (SRH) conditions.
