# Supplementary material for: Phylogenomic analysis of Apoidea sheds new light on the sister group of bees
Source: BMC Evol Biol. 2018 May 18;18:71. doi: 10.1186/s12862-018-1155-8 (PMC5960199; doi:10.1186/s12862-018-1155-8)
Supplement: Supplementary file 3 — Table 1. Information on grouping of species in a given quartet when assessing the phylogenetic position of Ammoplanina (two species) and Mellininae (one species) from analyzing the amino acid supermatrix and nucleotide supermatrix including all three codon positions via Four-cluster Likelihood Mapping (FcLM). (PDF 652 kb) [file 12862_2018_1155_MOESM3_ESM.pdf]

Additional table 1: Information on grouping of species in a given quartet when assessing the phylogenetic position of Ammoplanina (two species) and Mellininae (one species) from analyzing the amino acid supermatrix and nucleotide supermatrix including all three codon positions via Four-cluster Likelihood Mapping (FcLM).

Hypothesis 1 - Position Ammoplanina

Group	Taxonomic category (according to Pulawski's Catalog of Sphecidae 2016)	Included species
1	Anthophila (bees)	Ammobates_syriacus
		Andrena_vaga
		Anthidium_manicatum
		Anthophora_plumipes
		Apis_mellifera
		Apis_mellifera2
		Bombus_rupestris
		Camptopoeum_sacrum
		Ceratina_chalybea
		Chelostoma_florisomne
		Coelioxys_conoidea
		Colletes_cunicularius
		Stenotritidae
		Dasypoda_hirtipes
		Dioxys_cincta
		Dufourea_dentiventris
		Epeolus_variegatus
		Eucera_nigrescens
		Eucera_plumigera
		Eucera_syriaca
		Euglossa_dilemma
		Halictus_quadricinctus
		Heriades_truncorum
		Hylaeus_variegatus
		Lasioglossum_xanthopus
		Lithurgus_chrysurus
		Macropis_fulvipes
		Megachile_willughbiella
		Melitta_haemorrhoidalis
		Nomada_lathburiana
		Nomia_diversipes
		Nomioides_sp
		Osmia_cornuta
		Panurgus_dentipes
		Sphecodes_albilabris
		Stelis_punctulatissima
		Systropha_curvicornis
		Tetragonula_carbonaria
		Tetralonia_malvae
		Tetraloniella_nigriceps
		Tetraloniella_sp
		Thyreus_orbatus
		Xylocopa_violacea
2	Ammoplanina	Ammoplanellus_spec
		Ammoplanus_spec
3	Psenini + Odontosphecini	Mimesa_lutaria
		Odontosphex_paradoxus
		Psenulus_dilectus
		Psenulus_fuscipennis
		Psenulus_spec
4	Remaining apoid wasps and outgroups	Acanthostethus_spec
		Acromyrmex_echinatior
		Alysson_spinosus
		Ammatomus_spec1
		Ammatomus_spec2
		Ammophila_sabulosa
		Ammophila_spec2
		Ampulex_compressa
		Ampulex_fasciata
		Anacrabro_spec
		Arigorytes_spec
		Astata_minor
		Astata_spec1
		Astata_spec2
		Belomicrus_spec
		Bembecinus_spec2
		Bembix_rostrata
		Bicyrtes_ventralis
		Brachystegus_spec
		Camponotus_floridanus
		Carinostigmus_spec
		Cerceris_acanthophila
		Cerceris_arenaria
		Chalybion_californicum
		Chalybion_spec1
		Chalybion_spec2
		Chlorion_hirtum
		Chlorion_spec
		Clypeadon_sculleni2
		Clypeadon_taurulus
		Colpa_sexmaculata
		Crabro_peltarius
		Crabro_scutellatus2
		Crossocerus_quadrimaculatus
		Dasyproctus_spec
		Didineis_spec
		Dinetus_pictus
		Diodontus_minutus
		Dolichurus_corniculus
		Dolichurus_sp
		Dryudella_pinguis
		Dryudella_spec
		Dynatus_burmeisteri
		Ectemnius_sonorensis
		Entomognathus_spec
		Eremnophila_melanaria
		Eucerceris_arenaria
		Foxita_bara_ssp_patei1
		Foxita_bara_ssp_patei2
		Gorytes_albidulus
		Gorytes_laticinctus
		Harpactus_elegans
		Harpegnathos_saltator
		Heliocausus_spec
		Heterogyna_nocticola
		Holotachysphex_spec
		Hoplisoides_spec
		Isodontia_mexicana
		Larra_spec
		Larropsis_spec
		Lestica_alata
		Lestica_clypeata
		Lestica_spec
		Lindenius_panzeri2
		Liogorytes_spec
		Liris_sp
		Liris_spec
		Lyroda_spec
		Mellinus_arvensis
		Microbembex_sp
		Microstictia_hurdi
		Moniaecera_spec
		Neodasyproctus_spec1
		Neodasyproctus_spec2
		Nitela_sp
		Nysson_niger
		Oxybelus_bipunctatus
		Palarus_histrio
		Palmodes_spec
		Paranysson_spec
		Passaloecus_eremita
		Pemphredon_lugens
		Penepodium_fumipenne
		Philanthus_gibbosus
		Philanthus_triangulum
		Pison_atrum
		Pison_spec
		Podalonia_hirsuta
		Podalonia_sp
		Polemistus_spec
		Pompilus_cinereus
		Prionyx_kirbii
		Prionyx_subatratus
		Prosopigastra_nearctica
		Psammaecius_versicolor
		Pseudoplisus_willcoxi
		Pseudoscolia_sinaitica
		Pseudoscolia_spec
		Quexua_spec
		Rhopalum_clavipes
		Rubrica_nasuta
		Sapyga_quinquepunctata
		Sceliphron_curvatum
		Scolia_hirta
		Selman_notatus
		Smicromyrme_rufipes
		Sphecius_convallis
		Sphecius_spectabilis
		Sphex_funerarius
		Sphex_funerarius3
		Sphodrotes_spec
		Spilomena_beata
		Stangeella_cyaniventris
		Steniolia_duplicata
		Stictia_heros
		Stictiella_spec
		Stigmus_spec1
		Stizoides_foxi
		Stizoides_tridentatus
		Stizus_aff_marthae
		Stizus_continuus
		Stizus_vespoides
		Synnevrus_spec
		Tachysphex_fulvitarsis
		Tachysphex_spec
		Tachytes_amazonus
		Tachytes_spec
		Tanyoprymnus_moneduloides
		Tanyoprymnus_spec
		Tiguipa_spec
		Tiphia_femorata
		Trachypus_spec
		Trichostictia_spec
		Trypoxylon_figulus
		Trypoxylon_lactitarse

Hypothesis 2 - Position Mellininae

Group	Taxonomic category (according to Pulawski's Catalog of Sphecidae 2016)	Included species
1	Mellininae	Mellinus_arvensis
2	Sphecidae	Ammophila_sabulosa
		Ammatomus_spec1
		Ammophila_spec2
		Chalybion_californicum
		Chalybion_spec1
		Chalybion_spec2
		Chlorion_hirtum
		Chlorion_spec
		Dynatus_burmeisteri
		Eremnophila_melanaria
		Isodontia_mexicana
		Palmodes_spec
		Penepodium_fumipenne
		Podalonia_hirsuta
		Podalonia_sp
		Prionyx_kirbii
		Prionyx_subatratus
		Sceliphron_curvatum
		Sphex_funerarius
		Sphex_funerarius3
		Stangeella_cyaniventris
3	Crabroninae + Dinetinae	Anacrabro_spec
		Belomicrus_spec
		Crabro_peltarius
		Crabro_scutellatus2
		Crossocerus_quadrimaculatus
		Dasyproctus_spec
		Dinetus_pictus
		Ectemnius_sonorensis
		Entomognathus_spec
		Foxita_bara_ssp_patei1
		Foxita_bara_ssp_patei2
		Holotachysphex_spec
		Larra_spec
		Larropsis_spec
		Lestica_alata
		Lestica_clypeata
		Lestica_spec
		Lindenius_panzeri2
		Liris_sp
		Liris_spec
		Lyroda_spec
		Moniaecera_spec
		Neodasyproctus_spec1
		Neodasyproctus_spec2
		Nitela_sp
		Oxybelus_bipunctatus
		Palarus_histrio
		Paranysson_spec
		Pison_atrum
		Pison_spec
		Prosopigastra_nearctica
		Quexua_spec
		Rhopalum_clavipes
		Sphodrotes_spec
		Tachysphex_fulvitarsis
		Tachysphex_spec
		Tachytes_amazonus
		Tachytes_spec
		Trypoxylon_figulus
		Trypoxylon_lactitarse
4	Remaining apoid wasps bees and outgroups	Ammoplanellus_spec
		Acanthostethus_spec
		Acromyrmex_echinatior
		Alysson_spinosus
		Ammatomus_spec2
		Ammobates_syriacus
		Ammoplanus_spec
		Ampulex_compressa
		Ampulex_fasciata
		Andrena_vaga
		Anthidium_manicatum
		Anthophora_plumipes
		Apis_mellifera
		Apis_mellifera2
		Arigorytes_spec
		Astata_minor
		Astata_spec1
		Astata_spec2
		Bembecinus_spec2
		Bembix_rostrata
		Bicyrtes_ventralis
		Bombus_rupestris
		Brachystegus_spec
		Camponotus_floridanus
		Camptopoeum_sacrum
		Carinostigmus_spec
		Ceratina_chalybea
		Cerceris_acanthophila
		Cerceris_arenaria
		Chelostoma_florisomne
		Clypeadon_sculleni2
		Clypeadon_taurulus
		Coelioxys_conoidea
		Colletes_cunicularius
		Colpa_sexmaculata
		Dasypoda_hirtipes
		Didineis_spec
		Diodontus_minutus
		Dioxys_cincta
		Dolichurus_corniculus
		Dolichurus_sp
		Dryudella_pinguis
		Dryudella_spec
		Dufourea_dentiventris
		Epeolus_variegatus
		Eucera_nigrescens
		Eucera_plumigera
		Eucera_syriaca
		Eucerceris_arenaria
		Euglossa_dilemma
		Gorytes_albidulus
		Gorytes_laticinctus
		Halictus_quadricinctus
		Harpactus_elegans
		Harpegnathos_saltator
		Heliocausus_spec
		Heriades_truncorum
		Heterogyna_nocticola
		Hoplisoides_spec
		Hylaeus_variegatus
		Lasioglossum_xanthopus
		Liogorytes_spec
		Lithurgus_chrysurus
		Macropis_fulvipes
		Megachile_willughbiella
		Melitta_haemorrhoidalis
		Microbembex_sp
		Microstictia_hurdi
		Mimesa_lutaria
		Nomada_lathburiana
		Nomia_diversipes
		Nomioides_sp
		Nysson_niger
		Odontosphex_paradoxus
		Osmia_cornuta
		Panurgus_dentipes
		Passaloecus_eremita
		Pemphredon_lugens
		Philanthus_gibbosus
		Philanthus_triangulum
		Polemistus_spec
		Pompilus_cinereus
		Psammaecius_versicolor
		Psenulus_dilectus
		Psenulus_fuscipennis
		Psenulus_spec
		Pseudoplisus_willcoxi
		Pseudoscolia_sinaitica
		Pseudoscolia_spec
		Rubrica_nasuta
		Sapyga_quinquepunctata
		Scolia_hirta
		Selman_notatus
		Smicromyrme_rufipes
		Sphecius_convallis
		Sphecius_spectabilis
		Sphecodes_albilabris
		Spilomena_beata
		Stelis_punctulatissima
		Steniolia_duplicata
		Stenotritidae
		Stictia_heros
		Stictiella_spec
		Stigmus_spec1
		Stizoides_foxi
		Stizoides_tridentatus
		Stizus_aff_marthae
		Stizus_continuus
		Stizus_vespoides
		Synnevrus_spec
		Systropha_curvicornis
		Tanyoprymnus_moneduloides
		Tanyoprymnus_spec
		Tetragonula_carbonaria
		Tetralonia_malvae
		Tetraloniella_nigriceps
		Tetraloniella_sp
		Thyreus_orbatus
		Tiguipa_spec
		Tiphia_femorata
		Trachypus_spec
		Trichostictia_spec
		Xylocopa_violacea
