# Supplementary material for: Phylogenomic analysis of Apoidea sheds new light on the sister group of bees
Source: BMC Evol Biol. 2018 May 18;18:71. doi: 10.1186/s12862-018-1155-8 (PMC5960199; doi:10.1186/s12862-018-1155-8)
Supplement: Supplementary file 4 — Figure 2. Results from the Four-cluster Likelihood Mapping showing the support for the possible relationship of Ammoplanina (two species), Psenini (four species) + Odontosphecini (one species), Anthophila (42 species) and all remaining species including outgroup species to each other (58050 quartets). Original amino acid supermatrix (94,869 amino acid sites) based on a protein domain-based partitioning scheme and analyzed with partition-specific substitution models (a), permutation scheme I with supermatrix as in a, but amino acids are permuted within partitions while retaining the specific distribution of missing data (b), permutation scheme II with supermatrix as in b, but replacing amino acids in each partition with randomly selected amino acids, using amino acid frequencies as given by the LG substitution matrix, while retaining the specific distribution of missing data (c) and permutation scheme III supermatrix as given in c, but with missing data being randomly permuted (d). Figure 3. Results from the Four-cluster Likelihood Mapping showing the support for the possible relationship of Mellininae (one species), Sphecidae (19 species), Crabroninae (39 species) + Dinetinae (one species), and all remaining species including bees and outgroup species to each other (103320 quartets). Original amino acid supermatrix (94,869 amino acid sites) based on a protein domain-based partitioning scheme and analyzed with partition-specific substitution models (a), permutation scheme I with supermatrix as in a, but amino acids are permuted within partitions while retaining the specific distribution of missing data (b), permutation scheme II with supermatrix as in b, but replacing amino acids in each partition with randomly selected amino acids, using amino acid frequencies as given by the LG substitution matrix, while retaining the specific distribution of missing data (c) and permutation scheme III supermatrix as given in c, but with missing data being randomly permuted (d). Fi [file 12862_2018_1155_MOESM4_ESM.doc]

**Phylogenomic analysis of Apoidea sheds new light on the sister group of bees**

Manuela Sann*^1,2,3^ (manuela.sann@biologie.uni-freiburg.de), Oliver Niehuis^2,3^ (oliver.niehuis@biologie.uni-freiburg.de), Ralph S. Peters^4^ (r.peters@leibniz-zfmk.de), Christoph Mayer^2^ (C.Mayer@leibniz-zfmk.de), Alexey Kozlov^5^ (alexey.kozlov@h-its.org), Lars Podsiadlowski^2^ (lars@cgae.de), Sarah Bank^2^ (sarah.bank89@gmail.com)**,** Karen Meusemann^2,3^ (mail@karen-meusemann.de), Bernhard Misof^2^ (bmisof@uni-bonn.de), Christoph Bleidorn^6,7^ (christoph.bleidorn@gmail.com), Michael Ohl*^1^ (Michael.Ohl@mfn-berlin.de)

**AFFILIATIONS**

^1^ Museum für Naturkunde, Leibniz Institute for Evolution and Biodiversity Science, Invalidenstraße 43, 10115 Berlin, Germany

^2^ Center for Molecular Biodiversity Research, Zoological Research Museum Alexander Koenig, Adenauerallee 160, 53113 Bonn, Germany

^3^ University of Freiburg, Institute of Biology I (Zoology), Evolutionary Biology and Animal Ecology, Hauptstr. 1, 79104 Freiburg, Germany

^4^ Center of Taxonomy and Evolutionary Research, Arthropoda Department, Zoological Research Museum Alexander Koenig, Adenauerallee 160, 53113 Bonn, Germany

^5^ HITS gGmbH, Heidelberg Institute for Theoretical Studies, Schloss-Wolfsbrunnenweg 35, 69118 Heidelberg, Germany

^6^ Georg-August-Universität Göttingen, Animal Evolution and Biodiversity, Untere Karspüle 2, 37073 Göttingen, Germany.

^7^ German Centre for Integrative Biodiversity Research (iDiv) Halle-Jena-Leipzig, Deutscher Platz 5e, 04103 Leipzig, Germany

**SUPPLEMENTARY INVENTORY**

**Supplementary results**

**Appendix**

**A)** New hierarchical classification of apoid wasps (Apoidea excl. Anthophila). (**Appendix**)

**B)** Schematic overview, over all processing steps that were implemented in the data analysis workflow.

**Supplementary Figures**

**Supplementary figure 1**

Information content (IC) of the protein-domain based data blocks of the amino acid supermatrix before and after removing data blocks showing an information content of zero.

**Supplementary figure 2**

Results from the Four-cluster Likelihood Mapping showing the support for the possible relationship of Ammoplanina (two species), Psenini (four species) + Odontosphecini (one species), Anthophila (42 species) and all remaining species including outgroup species to each other (58050 quartets). Original amino acid supermatrix (94,869 amino acid sites) based on a protein domain-based partitioning scheme and analyzed with partition-specific substitution models (**a**), permutation scheme I with supermatrix as in a, but amino acids are permuted within partitions while retaining the specific distribution of missing data (**b**), permutation scheme II with supermatrix as in b, but replacing amino acids in each partition with randomly selected amino acids, using amino acid frequencies as given by the LG substitution matrix, while retaining the specific distribution of missing data (**c**) and permutation scheme III supermatrix as given in c, but with missing data being randomly permuted (**d**).

**Supplementary figure 3**

Results from the Four-cluster Likelihood Mapping showing the support for the possible relationship of Mellininae (one species), Sphecidae (19 species), Crabroninae (39 species) + Dinetinae (one species), and all remaining species including bees and outgroup species to each other (103320 quartets). Original amino acid supermatrix (94,869 amino acid sites) based on a protein domain-based partitioning scheme and analyzed with partition-specific substitution models (**a**), permutation scheme I with supermatrix as in a, but amino acids are permuted within partitions while retaining the specific distribution of missing data (**b**), permutation scheme II with supermatrix as in b, but replacing amino acids in each partition with randomly selected amino acids, using amino acid frequencies as given by the LG substitution matrix, while retaining the specific distribution of missing data (**c**) and permutation scheme III supermatrix as given in c, but with missing data being randomly permuted (**d**).

**Supplementary figure 4**

Results from the Four-cluster Likelihood Mapping showing the support for the possible relationship of Ammoplanina (two species), Psenini (four species) + Odontosphecini (one species), Anthophila (42 species) and all remaining species including outgroup species to each other (58050 quartets). Original nucleotide supermatrix (284.607 nucleotide sites) partitioned based on applying a combination of protein domain – and codon-based partitioning scheme by modelling the 1^st^, 2^nd^ and 3^rd^ codon position separately. Each partition was analyzed with partition-specific model parameters under the nucleotide substitution model GTR+G (**a**), permutation scheme I with supermatrix as in a, but nucleotides are permuted within partitions while retaining the specific distribution of missing data (**b**), permutation scheme II with supermatrix as in b, but replacing nucleotides in each partition with randomly selected nucleotides, while retaining the specific distribution of missing data (**c**) and permutation scheme III supermatrix as given in c, but with missing data being randomly permuted (**d**).

**Supplementary figure 5**

Results from the Four-cluster Likelihood Mapping showing the support for the possible relationship of Mellininae (one species), Sphecidae (19 species), Crabroninae (39 species) + Dinetinae (one species), and all remaining species including bees and outgroup species to each other (103320 quartets). Original nucleotide supermatrix (284.607 nucleotide sites) partitioned based on applying a combination of protein domain – and codon-based partitioning scheme by modelling the 1^st^, 2^nd^ and 3^rd^ codon position separately. Each partition was analyzed with partition-specific model parameters under the nucleotide substitution model GTR+G (**a**), permutation scheme I with supermatrix as in a, but nucleotides are permuted within partitions while retaining the specific distribution of missing data (**b**), permutation scheme II with supermatrix as in b, but replacing nucleotides in each partition with randomly selected nucleotides, while retaining the specific distribution of missing data (**c**) and permutation scheme III supermatrix as given in c, but with missing data being randomly permuted (**d**).

**Supplementary results**

**1. Sequencing of enriched target DNA libraries**

We obtained on average 980,955 paired-end raw reads per species (min: 67,065; mdn: 779,083; max: 3,408,960; Supplementary table S7). *De novo* assembly of these reads resulted on average in 17,551 contigs (min: 932; mdn: 12,931; max: 76,369; Supplementary table S7). After having compared all contigs sequenced on the same lane against each other, we removed on average 7.1 % potential cross-contamination contigs per species (Supplementary table S7). The calculated base-coverage depth of on-target contigs (*C_t_*) referring to target genes was on average 966,9 (min: 172,6; mdn: 616,75; max: 5,193) across all species (Supplementary table S7). The overall enrichment success was 71 % (min: 4 %; mdn: 81 %; max: 91 %).

**2. Identification of target gene contigs**

We found per species on average 139 of the 195 target genes in the sequenced and assembled DNA libraries (min: 7; mdn: 157; max: 177) and 187 of the 195 target genes in the available transcript libraries (min: 102; mdn: 191; max: 195; Supplementary table S7 and S8). The assemblies of 60 species comprised more than 150 target genes, those of 22 species comprised 101–150, and those of nine species comprised 51–100 target genes. Less than 50 target genes were found in the following six species: *Heterogyna nocticola* (seven target genes), *Heliocausus* sp*.* (eleven target genes), *Tanyoprymnus moneduloides* (13 target genes), *Neodasyproctus* sp. II (18 target genes), and *Lindenius panzeri* (36 target genes).

**3. Removal of outlier sequences**

The search for outlier amino acid sequences in the 195 multiple sequence alignments revealed 68 putatively misaligned sequences. After applying the outlier alignment refinement procedure, 62 remaining outlier sequences were removed from the amino acid alignments and the corresponding nucleotide files.

**3.1 Identification of protein domains**

In the 195 multiple sequence gene alignments, we annotated 465 Pfam A domains (41.2 % of the sites) and 624 void data blocks (58.8 % of the sites). Determined domain or void regions were pooled into 406 different data blocks, referring to 94 Pfam A domains which belong to the same clan, 117 Pfam A domains (no clan association) and 195 void regions belonging to the corresponding gene.

**3.2 Multiple sequence alignment masking**

We identified on average 26.9 % of the sites in the 195 multiple amino acid sequence alignments as ambiguously or randomly aligned (min: 0.3 %; mdn: 24.5 %; max: 77.3 %) and removed these sites from the alignments at both the amino acid and nucleotide level.

**3.3 Supermatrix inference**

After removing all ambiguously aligned sites identified by Aliscore, we split the multiple sequence alignments and rearranged the resulting data blocks according to the annotation of protein domains and void regions according to genes. The rearrangement of the 406 data blocks, consisting of pooled Pfam-A domains of the same clan, Pfam-A domains without clan annotation and void regions pooled according to genes, resulted in 397 data blocks. Nine of the 406 data blocks were entirely removed by Aliscore since their amino acids could not be aligned unambiguously. The resulting data blocks were concatenated on both the amino acid and the nucleotide level. The resulting supermatrices comprised 96,616 amino acid sites and 289,848 nucleotide sites, respectively.

**3.4 Removal of uninformative data blocks and of data blocks with poor taxonomic coverage**

After removing all data blocks with an estimated information content of zero (13.4 % of the data blocks; Supplementary figure 1) with the software MARE, the dataset comprised 344 data blocks with 94,869 (amino acid supermatrix) and 284,607 (nucleotide supermatrix) sites, respectively. In this step we also checked the taxonomic coverage of all data blocks. Only data blocks with at least one representative of pre-defined taxonomic groups were retained (section 3.4). Since all 344 data blocks contained all required taxa no additional data blocks were removed.

**3.5 Inferred partition scheme and substitution models**

PartitionFinder suggested arranging the 344 data blocks in the amino acid supermatrix into 133 partitions (94,869 sites, InL: -2813190.02; AICc: 5629230.24). Of the 133 partitions, PartitionFinder suggested 33 % to be analyzed with the JTT+G substitution model, 33 % with the LG4X model, 27 % with the JTT+G+F model, 4 % with the LG+G model, 2 % with the BLOSSOM62+G+F model, and 1 % with the BLOSSOM62+G model. PartitionFinder suggested arranging the 344 original data blocks in the nucleotide supermatrix in: (**a**) Set PF-NT-1,2: (i.e., 1^st^ and 2^nd^ codon position modeled separately in each domain data block; 3^rd^ codon positions excluded) starting with 688 pre-defined data blocks, resulting in 402 partitions (189,738 sites; lnL: -3136902,94; AICc: 6282785,51) and (**b**) Set PF-NT-1,2,3: (i.e., all three codon positions modeled separately) starting with 1032 pre-defined data blocks, resulting in 657 partitions (284,607 sites; lnL: -10028691,68; AICc: 20071601,93).

**4. Phylogenetic analyses**

The bootstrap convergence criterion MRE implemented in RAxML indicated that 150 bootstrap replicates are sufficient to assess statistically reliable support values when analyzing both the amino acid supermatrix and the nucleotide supermatrix that included the 1^st^ and 2^nd^ codon position only. The same approach suggested 100 bootstrap replicates to be sufficient to estimate statistically reliable node support values when analyzing the nucleotide supermatrix that contained all three codon positions (Supplementary figure S1–S3).

**5. Rogue taxon analysis**

We identified ten species that showed rogue taxon behavior in the phylogenetic analyses (Supplementary table S9). Specifically, when analyzing the amino acid supermatrix and the nucleotide supermatrix, the following species of apoid wasps exhibited rogue taxon behavior: *Heterogyna* *nocticola*, *Lindenius* *panzeri*, *Microstictia* *hurdi*, *Neodasyproctus* sp. II, *Dryudella* sp*.* and *Tanyoprymnus* *moneduloides.* Except for *M. hurdi* (represented in the supermatrices by the amino acid and nucleotide sequences of 165 different genes), these species are characterized in our analyses by the small number of genes whose sequences were exploited to place the species in the phylogenetic tree: seven (*H.* *nocticola*), 36 (*L.* *panzeri*), 18 (*N.* sp. II), 70 (*D.* sp.), and 13 (*T.* *moneduloides*). We also found four species of bees to exhibit rogue taxon behavior: *Ammobates* *syriacus* (192 genes), *Nomada* *lathburiana* (194 genes), *Tetraloniella* *nigriceps* (139 genes), and *Tetraloniella* sp. (128 genes). Finally, we identified one outgroup species as rogue taxon: *Pompilus* *cinereus* (191 genes). However, the rogue behavior of the above listed species did not affect the resolution and/or the support in the consensus tree of the major lineages that we identified for the here proposed new classification or for our major inferences on the evolutionary history of Apoidea.

**Appendix**

**A)** New hierarchical classification of apoid wasps (Apoidea excl. Anthophila) as outlined in the main text.

Superfamily: Apoidea Latreille, 1802

Family: Heterogynaidae Nagy, 1969

Family: Ampulicidae Shuckard, 1840

Subfamily: Ampulicinae Shuckard, 1840

Subfamily: Dolichurinae Lepeletier, 1845

Family: Astatidae Lepeletier, 1845

Family: Pemphredonidae Dahlbom, 1835

Subfamily: Pemphredoninae Dahlbom, 1835

Subfamily: Spilomeninae Menke, 1989

Subfamily: Stigminae Bohart und Menke, 1976

Family: Philanthidae Latreille, 1802

Family: Psenidae Costa, 1858

Subfamily: Pseninae Costa, 1858

Subfamily: Odontosphecinae Menke, 1967

Family: Ammoplanidae Evans 1959

Family: Bembicidae Latreille, 1802

Subfamily: Bembicinae Latreille, 1802

Subfamily: Nyssoninae Latreille, 1804

Family: Mellinidae Latreille, 1802

Family: Sphecidae Latreille, 1802

Subfamily: Ammophilinae André, 1886

Subfamily: Chlorontinae Fernald, 1905

Subfamily: Sceliphrinae Ashmead, 1899

Subfamily: Sphecinae Latreille, 1802

Family: Crabronidae Latreille, 1802

Subfamily: Crabroninae Latreille, 1802

Subfamily: Larrinae Latreille, 1810

Subfamily: Dinetinae Fox, 1895

**Supplementary figures:**
